# Insulin/IGF-Regulated Size Scaling of Neuroendocrine Cells Expressing the bHLH Transcription Factor *Dimmed* in *Drosophila*

**DOI:** 10.1371/journal.pgen.1004052

**Published:** 2013-12-26

**Authors:** Jiangnan Luo, Yiting Liu, Dick R. Nässel

**Affiliations:** Department of Zoology, Stockholm University, Stockholm, Sweden; Washington University Medical School, United States of America

## Abstract

Neurons and other cells display a large variation in size in an organism. Thus, a fundamental question is how growth of individual cells and their organelles is regulated. Is size scaling of individual neurons regulated post-mitotically, independent of growth of the entire CNS? Although the role of insulin/IGF-signaling (IIS) in growth of tissues and whole organisms is well established, it is not known whether it regulates the size of individual neurons. We therefore studied the role of IIS in the size scaling of neurons in the *Drosophila* CNS. By targeted genetic manipulations of insulin receptor (dInR) expression in a variety of neuron types we demonstrate that the cell size is affected only in neuroendocrine cells specified by the bHLH transcription factor DIMMED (DIMM). Several populations of DIMM-positive neurons tested displayed enlarged cell bodies after overexpression of the dInR, as well as PI3 kinase and Akt1 (protein kinase B), whereas DIMM-negative neurons did not respond to dInR manipulations. Knockdown of these components produce the opposite phenotype. Increased growth can also be induced by targeted overexpression of nutrient-dependent TOR (target of rapamycin) signaling components, such as Rheb (small GTPase), TOR and S6K (S6 kinase). After *Dimm*-knockdown in neuroendocrine cells manipulations of dInR expression have significantly less effects on cell size. We also show that dInR expression in neuroendocrine cells can be altered by up or down-regulation of *Dimm*. This novel dInR-regulated size scaling is seen during postembryonic development, continues in the aging adult and is diet dependent. The increase in cell size includes cell body, axon terminations, nucleus and Golgi apparatus. We suggest that the dInR-mediated scaling of neuroendocrine cells is part of a plasticity that adapts the secretory capacity to changing physiological conditions and nutrient-dependent organismal growth.

## Author Summary

Nerve cells display a large variation in size in an organism. Thus, a fundamental question is how growth of individual cells and their organelles is regulated. We ask if there is a regulatory mechanism for scaling the size of individual nerve cells, independent of the growth of the entire central nervous system (CNS). Growth of tissues and whole organisms depends on insulin/insulin-like growth factor signaling (IIS), but it is not known whether IIS regulates the size of individual nerve cells. We therefore studied the role of IIS in the size scaling of neurons in the CNS of the fruitfly *Drosophila*. By targeted genetic manipulations of insulin receptor (dInR) expression in a variety of neuron types we demonstrate that the cell size is affected only in neuroendocrine cells specified by the transcription factor DIMMED (DIMM). DIMM-positive neurons displayed enlarged cell bodies after overexpression of the dInR and downstream signaling components, whereas DIMM-negative neurons did not. Knockdown of these components results in smaller neurons. This novel dInR-regulated size scaling is seen during postembryonic development, continues in the aging adult and is diet dependent. We suggest that the dInR-mediated scaling of neuroendocrine cells is part of a plasticity that adapts the secretory capacity (neurohormone production) to changing physiological conditions and nutrient-dependent organismal growth.

## Introduction

Neurons and other cells display substantial variation in size in an organism. Thus, some neuron types in the brain have large cell bodies and extensive axonal processes, whereas others are minute with restricted branches. An important question is to what extent neuron size is genetically programmed and how much growth regulation is dependent on extrinsic factors, including systemic signaling. Although there appears to be an optimal size for most cell types there is room for plasticity, for instance to accommodate for availability of nutrients during development [1]–[4]. We ask here whether there is a dynamic growth-regulation of individual neurons by systemic or paracrine factors and how allometric size scaling of cells is controlled in the CNS. These fundamental questions have not been addressed in detail previously, and available data primarily deal with growth of entire tissues or whole organisms [2]–[6].

In general growth of cells, tissues and whole organisms depends on a combination of cell autonomous nutrient sensing via the TOR (target of rapamycin) pathway and insulin/IGF signaling (IIS), both of which are evolutionarily conserved pathways [3], [7]–[15]. During organ growth both cell size and cell numbers can be regulated by components of the IIS and TOR pathways (see [3], [16], [17]). Growth of ensembles of neuroblasts and post-mitotic neurons is regulated by similar mechanisms to ensure appropriate cell proliferation and subsequent differentiation of mature properties, including size of the cell body. Neuroblasts and other stem cells remain part of their time in a quiescent state, a reversible arrest of growth and cell division [18]–[20]. Exit from this arrested state involves induction of cell growth and is nutrition dependent [21]–[23]. In feeding larvae of *Drosophila* the postembryonic neuroblasts (progenitors of imaginal neurons) are protected against malnutrition. Thus, whereas many tissues display reduced growth after restricted nutrition, neuroblast growth is maintained by anaplastic lymphoma kinase (ALK) signaling, and therefore the CNS as a whole grows almost normally [16], [17], [24].

What mechanisms regulate size scaling of neurons and during what part of organismal development does it occur? Neuron growth is a likely part of the post-mitotic cell differentiation process where for instance neurons destined to become peptidergic neuroendocrine cells develop specific properties, including relatively large cell bodies [25]–[27]. Thus, in snails it was shown that cell bodies of large efferent peptidergic neurons and neuroendocrine cells continue to grow in a nutrient dependent manner as the organism grows [27]. In *Drosophila* a class of about 300 dedicated neuroendocrine cells have been identified that are specified by the bHLH transcription factor Dimmed (DIMM) and develop a capacity for production, packaging and releasing large amounts of neuropeptide or peptide hormone [28]–[30]. Part of this specification seems to involve an enlargement of cell body size relative to many surrounding interneurons. An important question is to what extent individual neuron size is regulated cell autonomously, or by factors in its immediate niche, and how much depends on additional systemic signals. Allometric size regulation by means of systemic factors would be a suitable mechanism to regulate growth of post-mitotic neurons that need to adapt their secretory capacity to changes in body volume or physiological requirements to function optimally.

We ask here whether the size of individual post-mitotic neurons can be regulated by systemic IIS, and whether growth can be induced in specific neuron types without their neighbors growing. To address these questions, we analyzed neuron size scaling by exploring the effect of manipulating expression of the insulin receptor (dInR) and signaling components of the IIS and TOR pathways in specific sets of neurons in the CNS of *Drosophila* during development and in the mature organism. Initial experiments revealed a marked difference in growth of DIMM-positive and DIMM-negative neurons, with targeted IIS manipulations affecting growth in the former only. Select populations of DIMM-expressing neurons were therefore chosen for in-depth analysis. Overexpression of the dInR and some of the downstream signaling components (PI3K and Akt), as well as TOR components such as TOR, the small GTPase Rheb (Ras homolog enriched in brain) and S6 kinase (S6K) all caused increased cell body size in these neurons. Conversely, diminishing activity of these components leads to decreased neuron size. We detected no size effects of IIS manipulations in motor neurons, various interneurons or DIMM-negative neuroendocrine cells tested. Selective dInR-mediated growth control could, thus, provide plastic scaling and protection of secretory activity in neuroendocrine cells, during development or in the adult life, as a means to ensure hormone production appropriate for body volume.

## Results

### Manipulation of dInR expression in specific neurons affects their size

**Figure 1:**
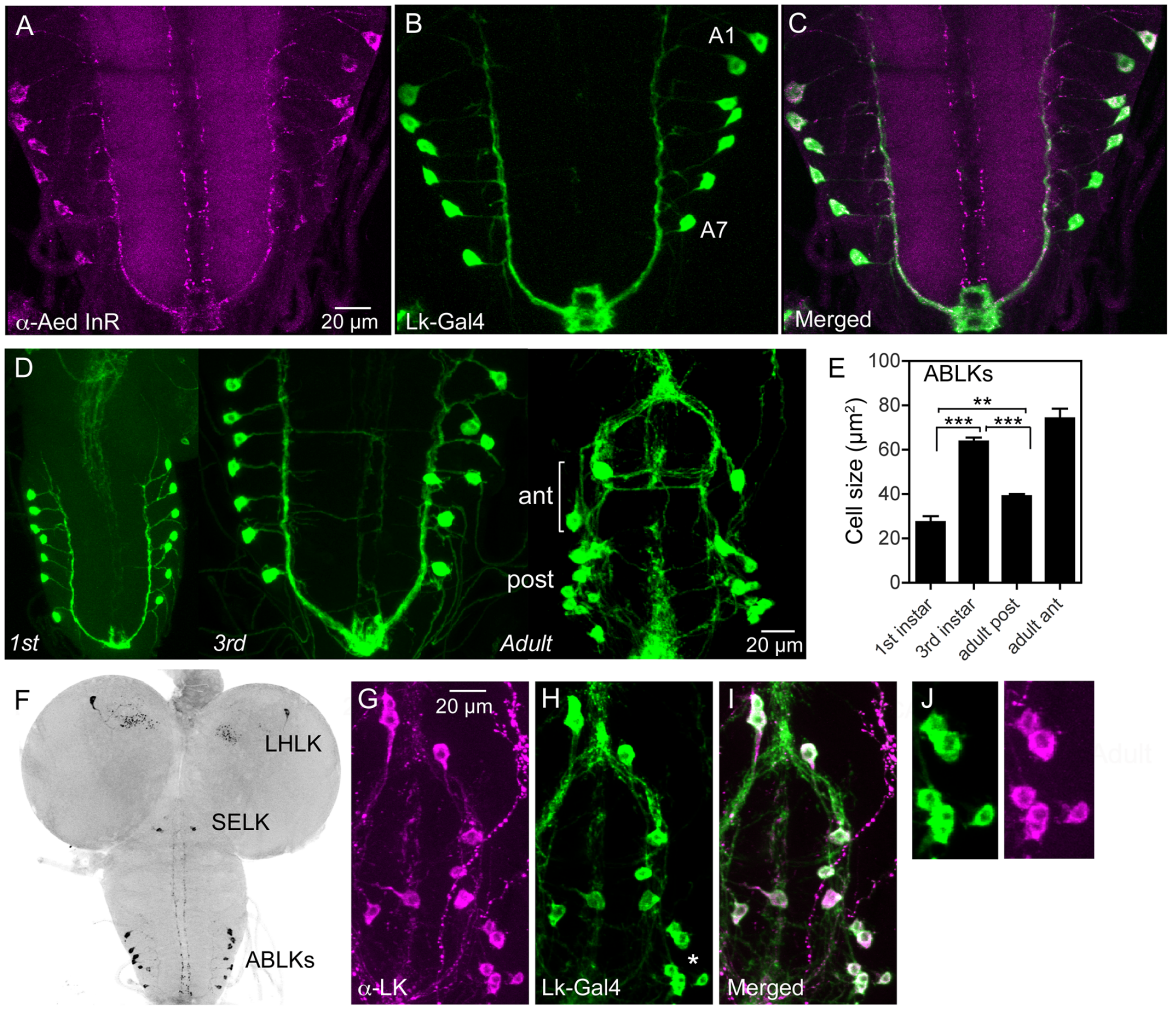
Distribution of insulin receptor protein in the larval CNS of *Drosophila* and growth of neurosecretory cells. **A–C** An antiserum to part of the insulin receptor (InR) of the mosquito *Aedes aegypti* labels neurosecretory cells (designated ABLKs) in the abdominal ganglia of the third instar larva that also produce the neuropeptide leucokinin (LK), visualized here with *Lk*-Gal4 driven GFP. Seven pairs of LK neurons coexpress the markers in abdominal segments A1–A7. **D** The cell bodies of LK neurons (ABLKs) in A1–7 grow substantially from first to third instar larvae, but not during pupal development. Thus, the posterior neurons (post) in the 3 d old adult fly (corresponding to ABLKs) are even smaller than in the late larva. However a set of 8 anterior adult-specific LK neurons (ant) have larger cell bodies than the ABLKs. LK neurons are visualized by *Lk*-Gal4-driven GFP. **E** Quantification of cell body sizes in larvae and 3 d old adult flies. The ABLKs (14 posterior neurons) are significantly larger in 3^rd^ instar larvae than in both 1^st^ instar and 3 d old adults (unpaired Student's T-test; **p<0.01, ***p<0.001; n = 5–6 animals for each developmental stage). **F** Overview of the larval CNS (3^rd^ instar) with LK-immunolabeled neurons, LHLK, SELK and ABLKs. **G–J** The immunolabeling of LK neurons with anti-LK (F) is a good marker for cell body size since it produces the same outline as membrane-targeted GFP (mcd8GFP) driven by the *Lk*-Gal4 (G). The posterior cell bodies marked by an asterisk are shown in higher magnification in **J**.

Since IIS plays an important role in growth regulation in the CNS as a whole, we decided to investigate the effect of targeted interference with the insulin receptor, dInR, in growth of individual neurons. It is likely that most, if not all, neurons and neuroblasts express the dInR, at least during developmental stages up to adult eclosion [21], [31]–[34]. To test this, we monitored receptor expression in the larval and adult CNS using three different insulin receptor antisera (described in material and methods). Two of these have been used previously in *Drosophila* to determine dInR localization in sensory cells of the antennae and germ line stem cells [35], [36]. All three antisera produced strong immunolabeling in the same set of neurons (Fig. 1A–C; Fig. S1, 2). In the third instar larva these were identical to a set of 20 neurons in the brain, subesophageal and abdominal ganglia expressing the neuropeptide leucokinin (LK) as seen by superposition with *Lk*-Gal4-driven GFP (Fig. 1A–C, F). In addition there is a weak receptor immunolabeling generally in the CNS that is insufficient to identify specific neurons, but suggests a more pan-neuronal expression. A similar set of insulin-receptor immunoreactive neurons was detected in the adult CNS and we confirmed expression of the receptor in the larval fat body (Fig. S1, 2). We could determine the effectiveness of our reagents by driving dInR-RNAi or UAS-dInR with the *Lk*-Gal4: this leads to reduction or increase of receptor immunolabeling in LK neurons, respectively (Fig. S1A–D). It was also clear that the size of the cell bodies of the 14 abdominal LK neurons (ABLK neurons) was altered by these dInR manipulations (Fig. S1C). Thus, we selected the ABLK neurons for a first analysis of the role of dInR in size regulation of neurons. Note that in all experiments cell body “size” was determined by measuring the largest surface area of each neuron in total z-projections of confocal images (given in µm^2^).

**Figure 2:**
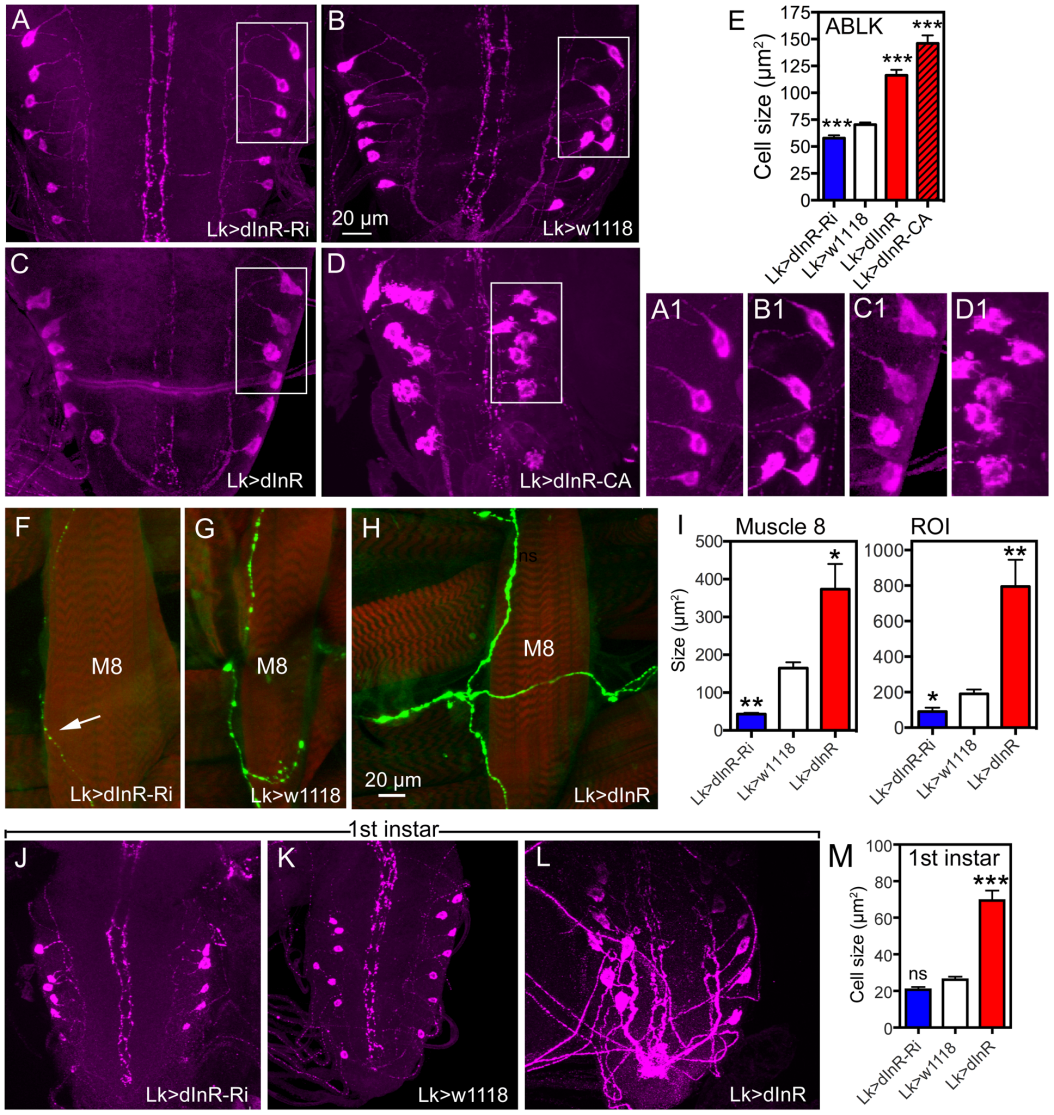
Manipulations of insulin receptor (dInR) levels alter size of the larval LK neurons in abdominal ganglia only. Neurons are visualized with anti-LK. In this and other figures cell body sizes are given as means (±SEM) of sizes of all specified neurons in a number (n) of specimens from at least 3 independent crosses for each genotype. **A–E** Knockdown or over expression of dInR using *Lk*-Gal4 alters the size of cell bodies (boxed areas are enlarged for details in A1–D1) monitored in late third instar larvae. The dInR-RNAi (Ri) significantly reduces size of cell bodies and, especially the constitutively active dInR (dInR-CA) drastically enlarges them. Note that the dInR-CA also induces a dislocation and irregular outlines of the cell bodies (D, D1). A quantification of the ABLK cell body size changes is shown in **E** (***p<0.001, n = 67–159 cell bodies from 6–13 animals of each genotype from 3 crosses; unpaired Student's T-test). **F–H** The peripheral axon processes of the ABLKs terminate primarily on body wall muscle 8 (M8). Muscles were stained with Rhodamine-phalloidin (red). Knockdown of dInR (in **F**) reduces axon diameter and bouton size of M8 branches compared to control (**G**), whereas overexpression of dInR leads to increased axon diameter, bouton size as well as increased branching of the axon (**H**). Axons were labeled with anti-LK and muscles with rhodamine-phalloidin. **I** Quantification of fluorescent area of axons on M8 in the three genotypes (*p<0.05, **p<0.01, n = 6–8 body walls for each genotype, 3 crosses; unpaired Student's T-test) and also in the total LK-immunolabeled axon termination in a region of interest (ROI) corresponding to the whole area shown in panel **H** (same for each genotype). This measurement provides combined information about branching area, as well as axon and bouton diameters (*p<0.05, **p<0.01, n = 6–8 body walls for each genotype from 3 crosses; unpaired Student's T-test). **J–M** The size of the ABLKs is altered already at the end of the first instar larval stage. Only the drastic increase caused by dInR overexpression is significant (***p<0.001, n = 5–10 animals for each genotype from 3 crosses; unpaired Student's T-test).

The seven pairs of segmentally arranged ABLKs are neurosecretory cells with cell bodies in the abdominal ganglia and axon terminations on body wall muscle [37], [38]. It has been established that all the neurons that label with the antiserum to LK are included in the *Lk*-Gal4 pattern [38] (see also Fig. 1G–J). Under normal feeding conditions the cell bodies of the 14 ABLKs grow substantially (113%) from first to late third instar larvae but do not grow further during pupal stages (Fig. 1D, E). During pupal development a set of 8 additional LK neurons appear anteriorly in the abdominal ganglia [37], [39]; these have far larger cell bodies than the 14 posterior ABLKs (Fig. 1D, E). In this study we monitored size changes in the ABLKs since their large cell bodies are segmentally arranged and readily visible in every specimen.

We used the *Lk*-Gal4 driver to alter dInR expression in LK-producing neurons by UAS-*dInR*-RNAi, UAS-*dInR* or UAS-*dInR^CA^*, a constitutively active receptor form. To monitor the cell body size we used antiserum to LK [40]. We could show that the LK-immunolabeling provides a good measure of cell body size since it matches the size seen with a membrane targeted GFP (*mcd8*-GFP) driven by the *Lk*-Gal4 (Fig. 1G–K). The effectiveness of the different UAS-dInR-constructs used has been described in several accounts [41]–[43]. The dInR manipulations led to significant cell body phenotypes in ABLK neurons. There was about 20% decrease in cell body size after diminishing dInR, and an increase of 65% for *dInR* and 107% for *dInR^CA^* overexpression (Fig. 2A–E, Table 1). In addition to the prominent increase in size, the *dInR^CA^* overexpression gave rise to irregular shapes of the cell bodies and a dislocation of the cell bodies resulting in a less distinct segmental distribution (Fig. 2D). In no experimental animal we found any indication that the number of LK neurons had changed, suggesting that size, but not cell proliferation, was affected by targeted dInR activity.

**Table 1:** Manipulations of genes in Dimm positive and Dimm negative peptidergic neuroendocrine cells.

Gal4	Cell type	Stage	Cell size after genetic manipulations (µm^2^)							
			*wildtype*	*dInR-Ri*	*dInR*	*dInR-CA*	*PI3K-DN*	*PI3K*	*Rheb-Ri*	*Rheb*
*Lk*	**ABLKs**	L1	26.1±1.7 n = 10	ns (5)	**+165%** (5)***	-	-	-	-	-
		L3	70.3±2.0 n = 13	**−18%** (7)***	**+65%** (7)***	**+107%** (6)***	**−35%** (8)***	**+116%** (7)***	ns (8)	**+93%** (7)***
		A 3 d ant	48.9±2.1 n = 7	ns (8)	**+78%** (9)***	-	**−18%** (6)**	**+121%** (9)***	**−20%** (5)**	**+122%** (9)***
		A 3 d post	24.3±1.1 n = 7	ns (7)	**+130%** (9)***	-	**−20%** (6)*	**+105%** (9)***	**−27%** (5)**	**+132%** (9)***
		A 35 d ant	52.2±2.6 n = 10	**−27%** (6)**	**+102%** (7)***	-	-	-	-	-
		A 35 d post	39.6±2.2 n = 10	**−39%** (6)***	**+114%** (7)***	-	-	-	-	-
*Dilp2*	**IPCs**	L3	59.2±2.0 n = 9	**−30%** (8)*	**+40%** (6)***	**+43%** (6)***	**−37%** (8)***	**+36%** (7)**	-	-
		A	84.0±5.4 n = 10	**−52%** (9)***	**+21%** (7)*	**+58%** (7)*	**−18%** (7)*	**+36%** (5)**	-	**Increase^1^**
*ptth*	**PTTH**	L3	105.3±15 n = 11	ns (15)	ns (10)	ns (6)	ns (5)	**+39%** (10)**	ns (8)	**+88%** (8)***
		A R-neur	46.7±2.5 n = 10	ns (5)	ns (6)	-	-	-	ns (5)	**+34%** (6)***

In this table only the PTTH neurons are DIMM negative. n = number of animals tested; numbers in brackets = number of animals tested,

p<0.05,

p<0.01,

p<0.001,

ns not significant (Unpaired Student's T-test), data are presented as mean values ± SEM. L1 = 1st instar larva, L3 = 3rd instar larva, A 3 d = 3 d old adult flies, A 35 d = 35 d old adult flies, ant = anterior LK neurons, post = posterior LK neurons, R-neur = R-neurons of ellipsoid body. **Increase^1^** Growth of IPCs was shown in Ref. [44]. Numerical data is provided in Table.

Each of the 14 larval ABLK neurons innervates muscle number 8 of the corresponding segment in the abdominal body wall [37]. To test whether the dInR-induced growth of the ABLK cell bodies is correlated with a growth of the peripheral axons we examined these axon terminations on muscle 8. Overexpression of the dInR in ABLK neurons increased the size of the axon terminations, and extra branches were seen (Fig. 2F–I). Conversely, *dInR*-RNAi in ABLKs led to thinner axons, smaller boutons and more restricted branching (Fig. 2F, G, I). Thus, dInR manipulations affected both the cell body size and the morphology of the peripheral axons. Importantly, we could show that the size of the ABLKs can be increased by dInR manipulations already when monitored in the first instar larva (Fig. 2J–M, Table 1). A very dramatic size increase (165%) compared to controls was noted.

Next we used a pan-neuronal driver, *elav*-Gal4, to manipulate the dInR widely in neurons, including the ABLK neurons. It was confirmed by anti-LK labeling that the *elav*-Gal4 pattern includes ABLK neurons, although the GFP expression is rather weak compared to most other neurons (not shown). *Elav*-driven *dInR-*RNAi led to smaller ABLK neurons (Fig. S3A,E), while expression of *dInR* or *dInR^CA^* did not result in larger ABLK neurons (Fig. S3C–E). This could be due to the *Elav* driver being weaker in the ABLK neurons or because the more global dInR activation induces a compensatory regulation of cell size (cell interactions preventing growth of the entire CNS). We also employed a driver that is intermediate between the *Lk*- and *Elav*-Gal4 in terms of neuron number, the c929-Gal4 [28], known to include the ABLKs [29]. Here, we found that the c929-driven dInR manipulations of neurons affected the size of the ABLK neurons at the same magnitude as the *Lk*-Gal4 driver (Fig. S3F–I).

### The size of ABLK neurons is affected also in adult flies

**Figure 3:**
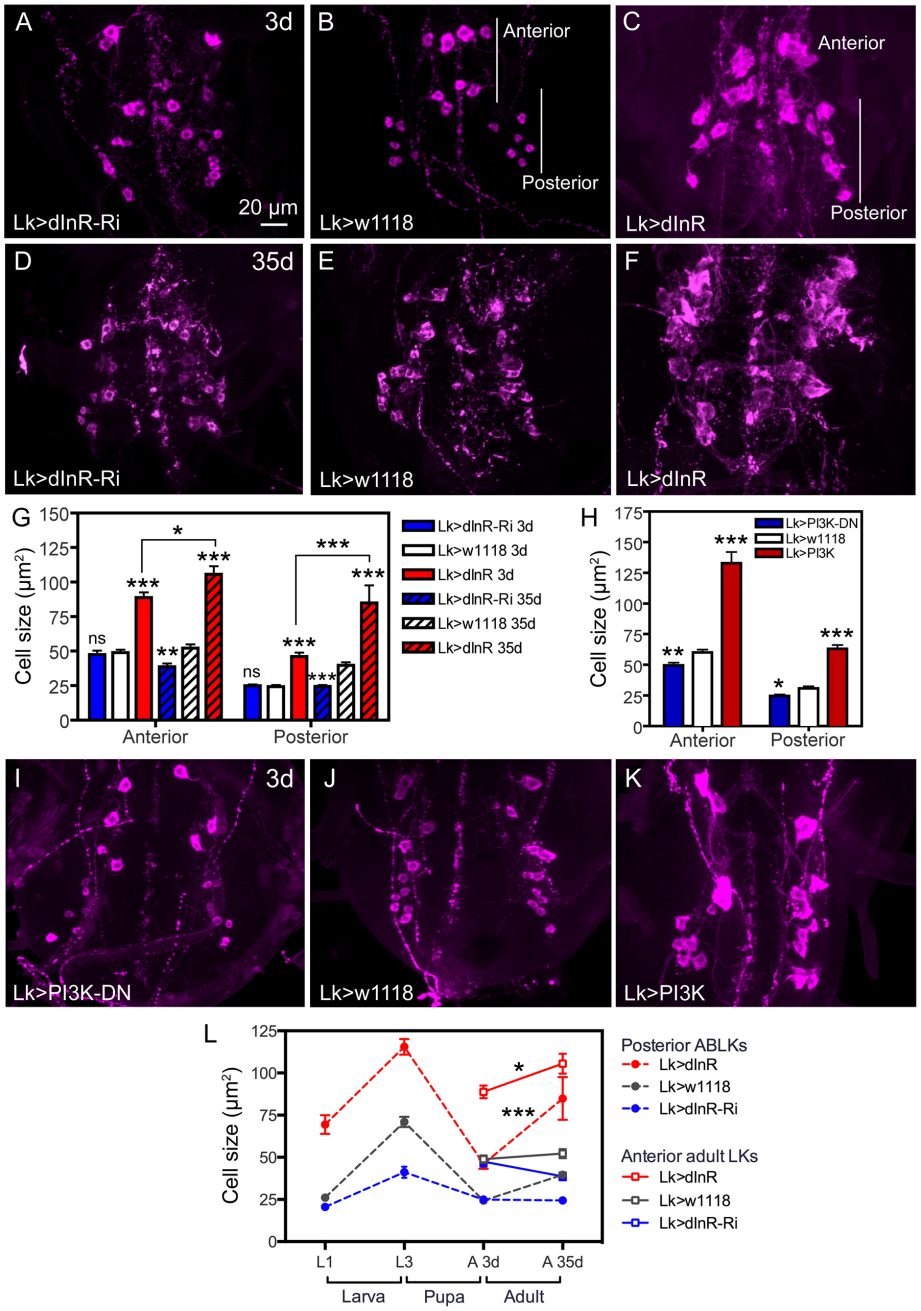
Manipulations of dInR in LK neurons also affect adult cell body size. Neurons are visualized with anti-LK. **A–C** Monitoring adult ABLKs (Posterior) and adult-specific anterior (Anterior) LK neurons in 3 d old adult flies reveal a size increase only (no effect of dInR-RNAi) for both the anterior adult-specific neurons (that differentiate during pupal development) and the posterior ABLKs. **D–F** In 35 d old flies the effect of the dInR manipulations are more drastic and also the dInR-RNAi has a significant effect on cell body size. **G** Quantification of cell body sizes in different genotypes and developmental time (3 d and 35 d old flies) in adults (*p<0.05, **p<0.01, ***p<0.001, n = 6–10 animals from 3 crosses for each genotype, unpaired Student's T-test; comparisons were made between the same neuron type of the same age). Note that both anterior and posterior ABLKs are significantly larger in 35 d old flies than in 3 d old ones (unpaired Student's T-test). **H–K** manipulations of PI3K also affect cell body size of anterior and posterior LK-neurons in 3 d old adult flies. A dominant negative PI3K (PI3K-DN) expressed in Lk-Gal4 neurons diminishes cell size in both anterior neurons and posterior ones, whereas overexpression of wild type PI3K drastically increases size of both neuron types. The cell sizes are quantified in **H** (*p<0.05, **p<0.01, ***p<0.001, n = 6–9 animals for each genotype from 3 crosses; unpaired Student's T-test). **L** Summary of sizes of abdominal LK neuron cell bodies in larvae and adults over time. We plotted the sizes of 14 ABLKs and their adult counterparts the posterior ABLKs (dashed lines), as well as the 8 adult-specific anterior LK neurons solid lines). In the wild type (or control) the size of the ABLKs increase from first to third instar larva, decreases during pupal development and then increases again from 3 d adult to 35 d old adults. The adult-specific anterior LK neurons do not increase significantly in size from 3–35 d in control flies. Over expression of dInR in LK neurons increases the ABLK cell body size (compared to controls), except during pupal development. The dInR-RNAi induces a significant difference only in the third instar and the 35 d old adults. Finally, after over expression of the dInR both the anterior adult-specific (red dashed line) and the posterior LK neurons (red solid line) display a significant increase in size after 35 d compared to 3 d (unpaired Student's T-test).

We also monitored the cell body size of abdominal LK neurons after dInR manipulations in adult male flies of two different ages (3 d and 35 d old flies). Monitoring 3 d old flies we found that overexpression of the receptor by the *Lk*-Gal4 driver leads to significantly larger cell bodies of both the 14 posterior ABLK neurons and the anterior adult-specific ones as compared to controls (Fig. 3A–C, G, Table 1). The dInR-RNAi was, however, inefficient in producing smaller cell body size in both types of abdominal LK neurons in the younger flies (Fig. 3A–C, G). Interestingly, we found that in 35 d old adult flies both the ABLKs and the anterior adult LK cells were significantly affected by both dInR knockdown and overexpression compared to 35 d controls and 3 d old flies (Fig. 3D–G, Table 1). After 35 d the dInR over expression resulted in very large cell bodies (more than 100% growth compared to 35 d controls) with highly irregular outlines (Fig. 3F). Especially the posterior ABLKs grow substantially compared to 3 d controls. This finding suggests that the abdominal LK neurons continuously respond to insulin signaling by altered cell size also in adult flies. Similar continuous growth of adult neurons was seen for mushroom body neurons and insulin producing cells (IPCs) in *Drosophila* after over expression of a component of the TOR pathway, the small GTPase Rheb [44]. For these neuron types 1 d and 21 d old flies were compared. The growth of larval and adult neurons over time is summarized in Fig. 3L.

### Manipulations of components downstream of the dInR and in the TOR pathway also produce size phenotypes in ABLKs

**Figure 4:**
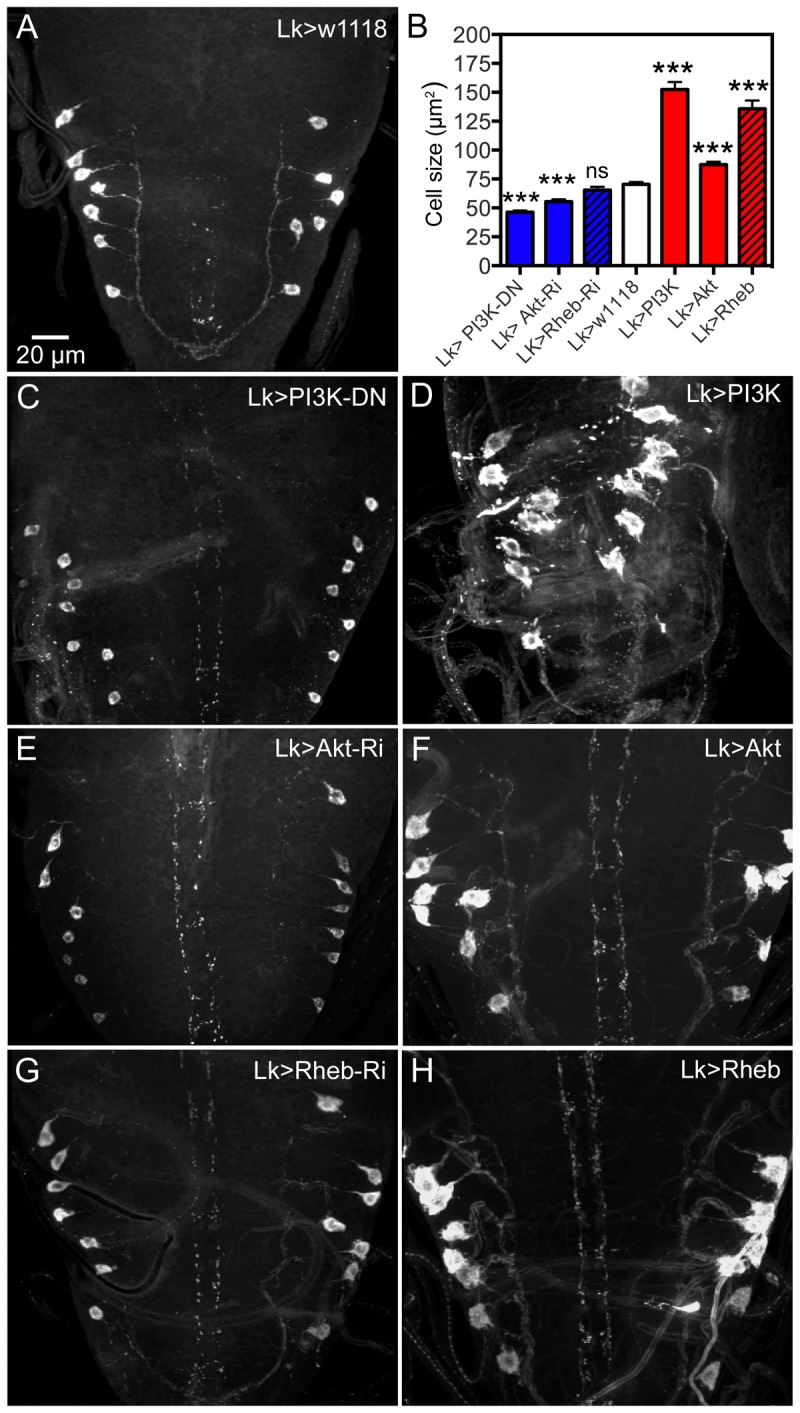
Manipulations of signaling components possibly downstream the dInR affect cell body size of larval ABLKs. Neurons are visualized with anti-LK. **A** ABLKs in control flies. **B** Quantification of cell body sizes of LK neurons in the different experiments. **C** and **D** expression of a dominant negative form of PI3K (PI3K-DN) diminishes cell body size and overexpression of wild type PI3K increases it (and leads to dislocation and irregular shapes of cell bodies). **E** and **F** Expressing Akt-RNAi and wild type Akt gives phenotypes similar to PI3K manipulations. **G** and **H** Overexpression of Rheb drastically increases cell body size, whereas Rheb-RNAi has no significant effect (***p<0.001, n = 8–14 animals for each genotype from 3 crosses; unpaired Student's T-test).

To further support that the cell size phenotypes in ABLKs depend on dInR-mediated signaling we manipulated the levels or activity of components that can act downstream of this receptor, such as PI3 kinase (PI3K) and the protein kinase B, Akt1 [see [7], [14], [15], [45]]. We expressed either a dominant-negative form of PI3K (*PI3K^DN^*) or a wild type PI3K in the ABLK neurons with the *Lk-Gal4*. LK immunolabeling in the larval CNS revealed that *PI3K^DN^* expression significantly decreased the size of ABLK cell bodies, while over expression resulted in enlarged ones with irregular outlines and displaced locations (Fig. 4A–D, Table 1). In the adult CNS overexpression of PI3K in LK neurons induces a substantial increase in both ABLK neurons and the adult-specific larger anterior LK neurons, whereas PI3K^DN^ expression significantly reduces cell body size (Fig. 3H–L, Table 1).

Manipulations of Akt1 expression in ABLK neurons by *Akt1*-RNAi and UAS-*Akt1* produced larval cell body phenotypes similar to PI3K and dInR, but without the irregular outlines (Fig. 4B,E, F, Table 1). We employed an antiserum to phosphorylated Akt (pAkt) to determine whether dInR manipulations in LK neurons affected Akt phosphorylation. In control flies the antiserum to pAkt labels a set of neurons in the CNS that includes the ABLKs and LHLKs, and mushroom body Kenyon cells, as well as several other sets of unidentified neurons (Fig. S4A,B, E–G). Using the c929-Gal4 driver we manipulated dInR levels and monitored pAkt immunolabeling levels and found that dInR over expression increased pAkt immunolabeling in what appeared to be ABLKs, but dInR-RNAi had no effect (Fig. S4C,D). There seems to be a basal Akt phosphorylation in control flies (Fig. S4A–D) and possibly this is independent of dInR signaling, explaining the lack of effect of dInR-RNAi (although we cannot exclude that the RNAi knockdown is too weak to affect the immunolabeling).

**Figure 5:**
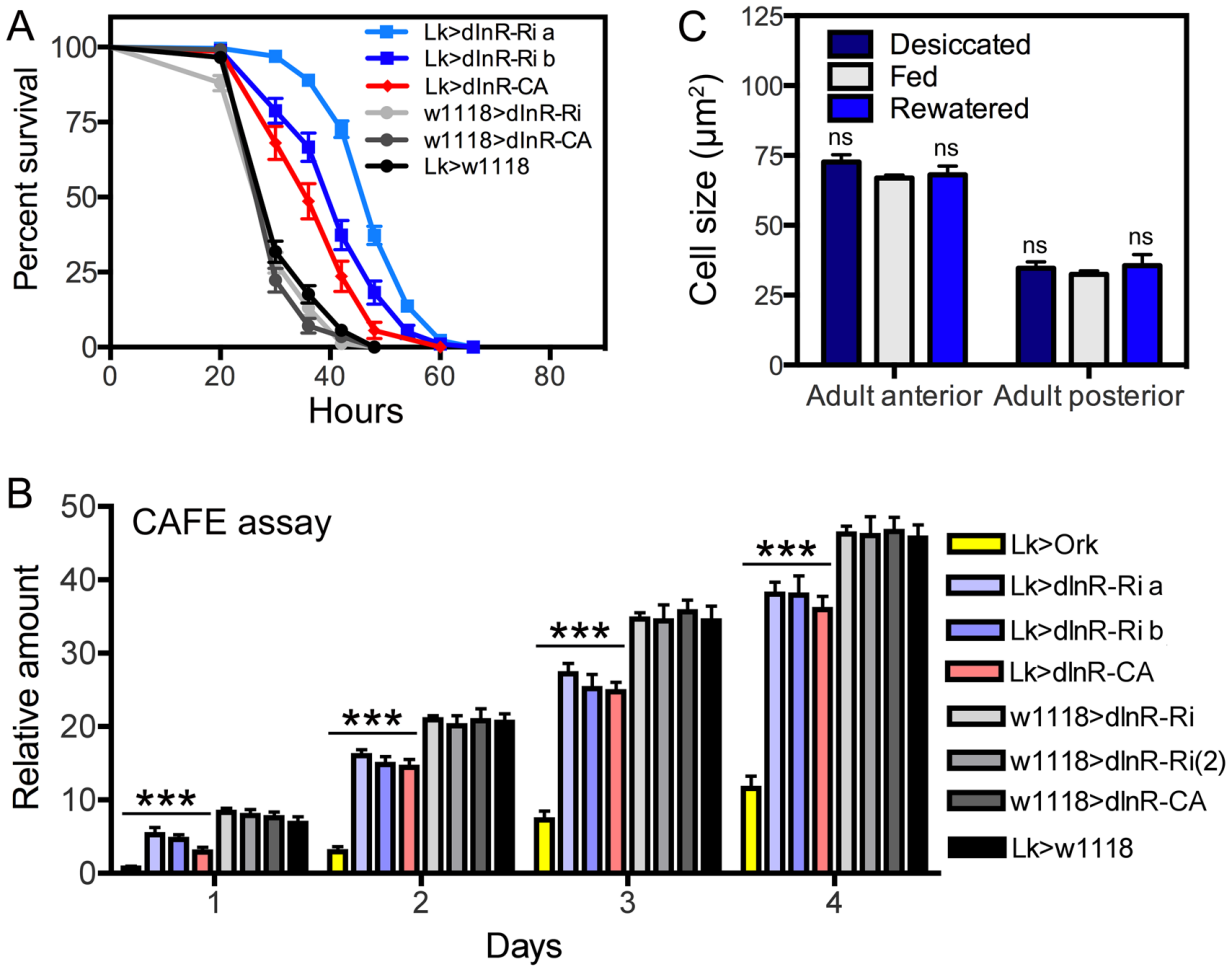
Functional effects of dInR manipulations of LK neurons in adult male flies. **A** Knockdown and overexpression of dInR in LK neurons produce the same phenotypes when flies are exposed to desiccation (dry starvation). Here the constitutively active dInR (dInR-CA) was used. Both manipulations lead to significantly increased resistance to desiccation, suggesting a malfunction in LK release that decreases diuresis in flies (***p<0.001, n = 120 flies for each genotype from 3 crosses; Log-rank (Mantel-Cox) Test). Note that two different dInR-RNAi lines were tested (a and b). **B** Feeding, as measured in CAFE assay, is decreased both in flies with dInR knockdown and over expression. Cumulative amount of food eaten over four days is displayed. As a control we used flies with LK neurons hyperpolarized with a constitutively active K-channel (Lk>Ork). These flies display strongly reduced feeding over four days, again suggesting that both manipulations of dInR levels produce malfunction of LK signaling; all manipulations (colored bars) produced significant decreases compared to the four controls shown in gray scale (***p<0.001, n = 10 flies for each genotype in 3 replicates, Two-way ANOVA). **C** To test whether cell body size of ABLK neurons is affected by activity (level of LK signaling) we exposed wild-type flies (w1118) to desiccation (dry starvation) for 18 h or 18 h desiccation followed by access to water (rewatered flies) for 2 h. As a control normally fed flies were used. No difference in cell body size was noted (ns, not significant for any comparison, n = 7–10 flies for each treatment, One-way ANOVA).

Another component that can be downstream of the dInR, but also be part of the TOR signaling pathway, is the small GTPase Rheb (Ras homolog enriched in brain) [46], [47] (see also Fig. S16). Thus, Rheb can either be controlled by dInR, PI3K, Akt1 and TSC1/2 (tuberous sclerosis tumor suppressor) and activate TOR to promote cell growth via S6 kinase and 4E-BP, or activate TOR in a nutrient-dependent pathway cell autonomously in growth control [46]–[48] (see also Fig. S16). It was recently shown that overexpression of Rheb in *Drosophila* IPCs and mushroom body Kenyon cells resulted in enlarged cell bodies of these neurons [44]. We thus tested whether manipulations of Rheb in LK neurons affected cell body size. Overexpression of Rheb in LK neurons led to strongly enlarged and disfigured cell bodies of ABLK neurons (Fig. 4B,H, Table 1). However, there was no significant effect of *Rheb*-RNAi on cell body size of ABLK neurons (Fig. 4B, G). We also analyzed 3 d old adult flies after Rheb manipulations in LK-neurons. Both the anterior and the posterior abdominal LK neurons displayed enlarged cell bodies after Rheb overexpression, and smaller ones after Rheb-RNAi (S Fig. 5, Table1).

Next we tried to determine whether dInR manipulations affect Rheb expression in ABLK neurons. When employing a Rheb antibody to the wild type larval CNS we found weak labeling in ABLK neurons and even less labeling elsewhere. Thus we were not able to detect any effects of dInR expression levels (not shown).

Two other components in the TOR signaling pathway were also investigated for their effect on ABLK cell body size: TOR and S6K. We expressed a dominant negative form of TOR under *Lk*-Gal4 control and observed decreased size of ABLKs in larvae (Fig. S6A, B, F). A dominant negative S6K also diminished the ABLK cell bodies, whereas a wild type and a constitutively active S6K increased their size (Fig. S6C–F). Thus we find that TOR signaling also affects the size of cell bodies of LK neurons.

When *Drosophila* larvae are exposed to conditions of restricted nutrients the growth of neuroblasts is ensured by anaplastic lymphoma kinase (Alk) signaling [16], [17], [24]. We therefore tested the effects of expressing wild type or a dominant negative form of Alk in LK neurons. As expected, we detected no effects of these manipulations under normal feeding conditions (Fig. S7A–C). Thus, we expressed a constitutively active form of Alk under *Lk*-Gal4 control to test its activity independent of the ligand Jelly belly. This had no effect on the size of ABLKs (Fig. S7D, E), possibly because the *Lk*-driver is active only post-mitotically in LK neurons [38], [39], [49].

### Manipulation of dInR expression in LK neurons affects their function

To test the effect of dInR manipulations on the function of the ABLK neurons we performed two different assays in adult flies, one for feeding and the other for diuretic action. These assays were selected since it is known that in *Drosophila* LK is a diuretic hormone [50]–[52] and plays a role in regulation of food intake [53]. We exposed adult male flies of different genotypes to dry starvation (desiccation) and monitored survival. Both knockdown and overexpression of dInR in the LK neurons resulted in flies that were more resistant to desiccation than controls, suggesting a reduced diuretic activity in both transgenes (Fig. 5A). Also in the capillary feeding (CAFE) assay we observed that both *dInR*-RNAi and overexpression lead to decreased feeding (Fig. 5B). As a control we tested the effect of hyperpolarizing LK neurons by expressing the open rectifier K^+^-channel Ork1 [54]. The expression of this channel did not affect the size of the ABLK cell bodies, but in both assays this inactivation of LK neurons resulted in phenotypes that suggest diminished LK signaling (Fig. 5B). Thus, the phenotypes seen after dInR-RNAi in LK neurons may also be caused by decreased LK signaling; perhaps as a result of smaller neurons and a diminished pool of LK peptide. However, the finding that overexpression of the dInR also may reduce LK signaling is harder to reconcile. We speculate that constitutive overexpression of the dInR induces overproduction of LK that offsets circulating hormone levels and thus physiology, or trigger growth effects that affect neuron function adversely.

Next we asked whether the physiological state of the flies could affect the size of the ABLK cell bodies, as a consequence of altered LK production and/or release. Since the ABLKs release LK as a diuretic hormone [51] we exposed flies (4–6 d old) to 18 h desiccation or 18 h desiccation followed by 2 h of access to water (re-watering). The size of the anterior and posterior LK neurons was monitored, compared to those of normally fed flies. There was no difference in cell body size between the experimental groups (Fig. 5C), although the intensity of LK immunoreactivity differed between them (not shown). Thus, the short-term physiological conditions tested do not affect cell body size of ABLK neurons.

### Manipulations of dInR in motor and interneurons do not affect cell size

**Figure 6:**
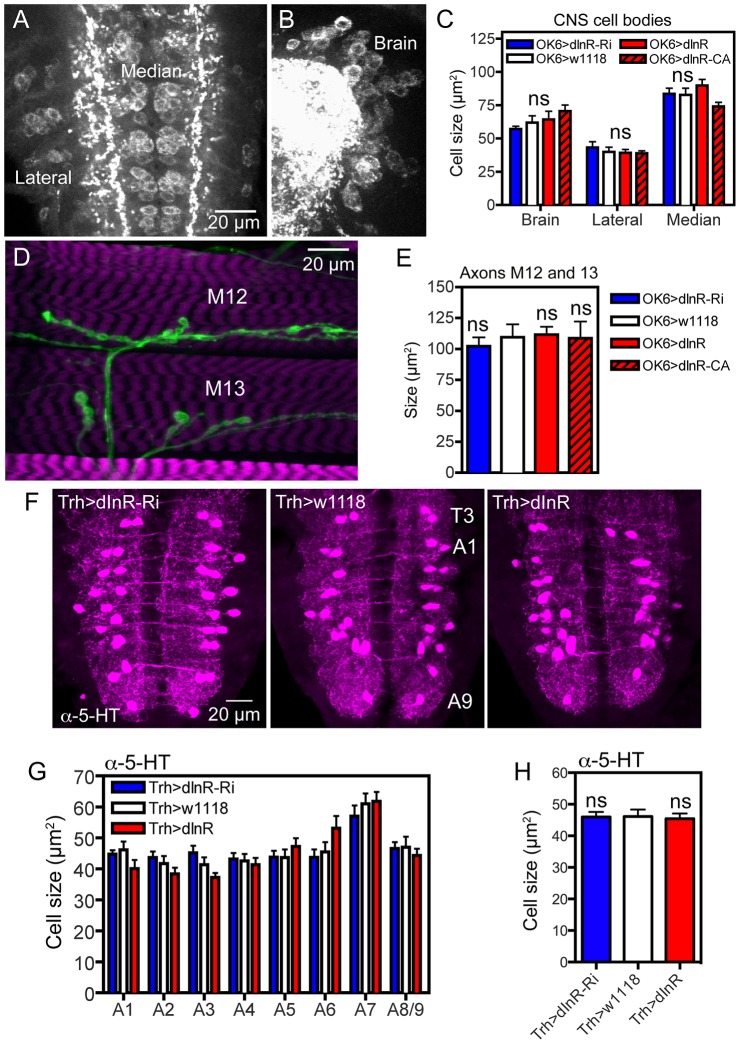
Manipulations of dInR expression in interneurons and motor neurons do not affect cell body size. For manipulations we used the OK6-Gal4 driver, known to identify interneurons in the brain and predominantly motor neurons in the ventral nerve cord. Many of the OK6 neurons are likely to be glutamatergic. **A** and **B** Distribution of vesicular glutamate transporter immunoreactivity in abdominal motor neurons (**A**), and brain interneurons (**B**) of control flies. Two sets of cell bodies were measured in abdominal ganglia lateral and median ones. **C** Neither dInR-RNAi, nor over expression of dInR or dInR^CA^ (dInR-CA) produced significant changes in cell body sizes of brain neurons or motor neurons (n = 6 animals for each genotype from 3 crosses, unpaired Student's T-test). **D** Analysis of the size of axon terminals of motor neurons on body wall muscles 12 and 13 (M12 and M13) was made after labeling with anti-HRP. **E** No effect was seen of dInR manipulations (n = 6–8 body walls for each genotype, unpaired Student's T-test). In Fig. S8 larger areas of body wall muscle innervation by motor neurons is shown for the different genotypes. **F** Manipulations of dInR levels in serotonergic neurons specified by a Trh-Gal4 driver did not affect cell body size of segmental interneurons in the abdominal ganglia (or elsewhere; not shown) revealed by anti-5HT immunolabeling. **G** Cell body size of 5-HT-immunolabeled interneurons in A1–A8/9 is not affected by dInR manipulations. **H** Average cell body size for all segments (ns, not significant, unpaired Student's T-test, n = 11–12 animals from 3 crosses).

To test whether the dInR-mediated growth is a feature of larger neurons with peripheral axon projections we manipulated the dInR in motor neurons. In the *Drosophila* ventral nerve cord of late embryos, around 80 motor neurons are stereotypically distributed in each segment [55]. They have distinct axonal projections into the periphery via the segmental nerve trunks and innervate specific target muscles of the body wall [56], [57]. We used the enhancer trap Gal4 line OK6, whose expression is predominantly in motor neurons and some interneurons in the larval CNS [58], [59], to drive *dInR*-RNAi, *dInR* and *dInR^CA^*. To monitor size of the cell bodies of motor neurons we employed an antiserum to a vesicular glutamate transporter (vGluT) known to be expressed in motor neurons [60] and to visualize their peripheral axons we used anti-HRP. Cell bodies of three types of neurons visualized by anti-vGluT antibody were measured: interneurons in the brain lobes, lateral motor neurons in segments A3–6 of the abdominal ganglia and median ones in the same segments. None of these neurons displayed any changes in cell body size after dInR manipulations (Fig. 6A–C, Table 2). Also the peripheral axonal projections of motor neurons on the body wall muscles displayed no obvious morphological effects of dInR manipulations (Fig. S8). The size of the axon terminals on muscles 12 and 13 were quantified and we found no effects of changing dInR levels (Fig. 6D, E).

**Table 2:** Manipulations of genes in different neuron types (peptidergic and non-peptidergic).

Gal4	Cell type	Stage	Cell size after genetic manipulations (µm^2^)			
			*wildtype*	*dInR-Ri*	*dInR*	*dInR-CA*
*Dilp7*	**DILP7 (A6–9+DP)**	L3	66.1±1.4 n = 18	**−13%** (10)**	**+82%** (12)***	-
*c929*	**Tv 1–3**	L3	62.1±5.1 n = 8	**−22%** (7)*	**+57%** (6)***	-
*pdf*	**Abd PDF**	L3	47.9±1.9 n = 8	ns (8)	**+61%** (9)**	-
	**l-LNv PDF**	A	86.9±12.2 n = 6	ns (6)	**+114%** (9)***	-
*ok6*	**Brain intern**	L3	61.9±5.1 n = 6	ns (6)	ns (6)	ns (6)
	**Abd intern**	L3	39.8±3.5 n = 6	ns (6)	ns (6)	ns (6)
	**Motoneuron**	L3	83.5±4.0 n = 6	ns (6)	ns (6)	ns (6)
*Trh*	**5-HT (A1–9)**	L3	46.1±2.2 n = 11	ns (12)	ns (11)	-

In this table *Dilp7*, *c929* and *pdf* neurons are DIMM positive, the *ok6* and *Trh* not. Brain intern = brain interneurons, Abd intern = abdominal ganglion interneurons, other abbreviations and statistics as in Table 1.

p<0.05,

p<0.01,

p<0.001,

ns not significant, data are presented as mean values ± SEM. Numerical data is provided in Table S2.

To test another type of interneurons we altered dInR levels in a set of serotonin-producing neurons defined by a *Trh*-Gal4 line [61]. We selected cell bodies of a set of 30 segmentally distributed serotonin-expressing interneurons in abdominal neuromeres A1–A9 for analysis. No differences in cell body size could be seen after dInR manipulations (Fig. 6 F–H, Table 2). Thus, both interneurons and motor neurons of the OK6 line and 5-HT-producing interneurons seem refractory to dInR induced size changes of the type seen in ABLKs. However, both interneurons and motor neurons grow in response to targeted overexpression of PI3K and Rheb as shown in neurons of the ellipsoid and mushroom bodies of the brain and in the neuromuscular junction [44], [45], [62], [63].

### Manipulation of dInR in peptidergic neurons that are DIMM positive

Since motor neurons and non-peptidergic interneurons seem not to display insulin-mediated size plasticity, whereas a set of peptidergic neurosecretory cells do, we asked whether this role of the dInR is general in neuroendocrine cells. Many of the peptidergic secretory and neuroendocrine cells express the bHLH transcription factor DIMM and it is known that DIMM expressing neurons are adapted to physiological demands for high secretory activity and often have large cell bodies [26], [29], [64]. We used the *c929*-Gal4, known to correspond to the DIMM expressing neurons [28], to manipulate the dInR in specific neurons. The c929 includes the ABLK neurons that also stain with DIMM antiserum from late embryo to adult stages [29], [65].

**Figure 7:**
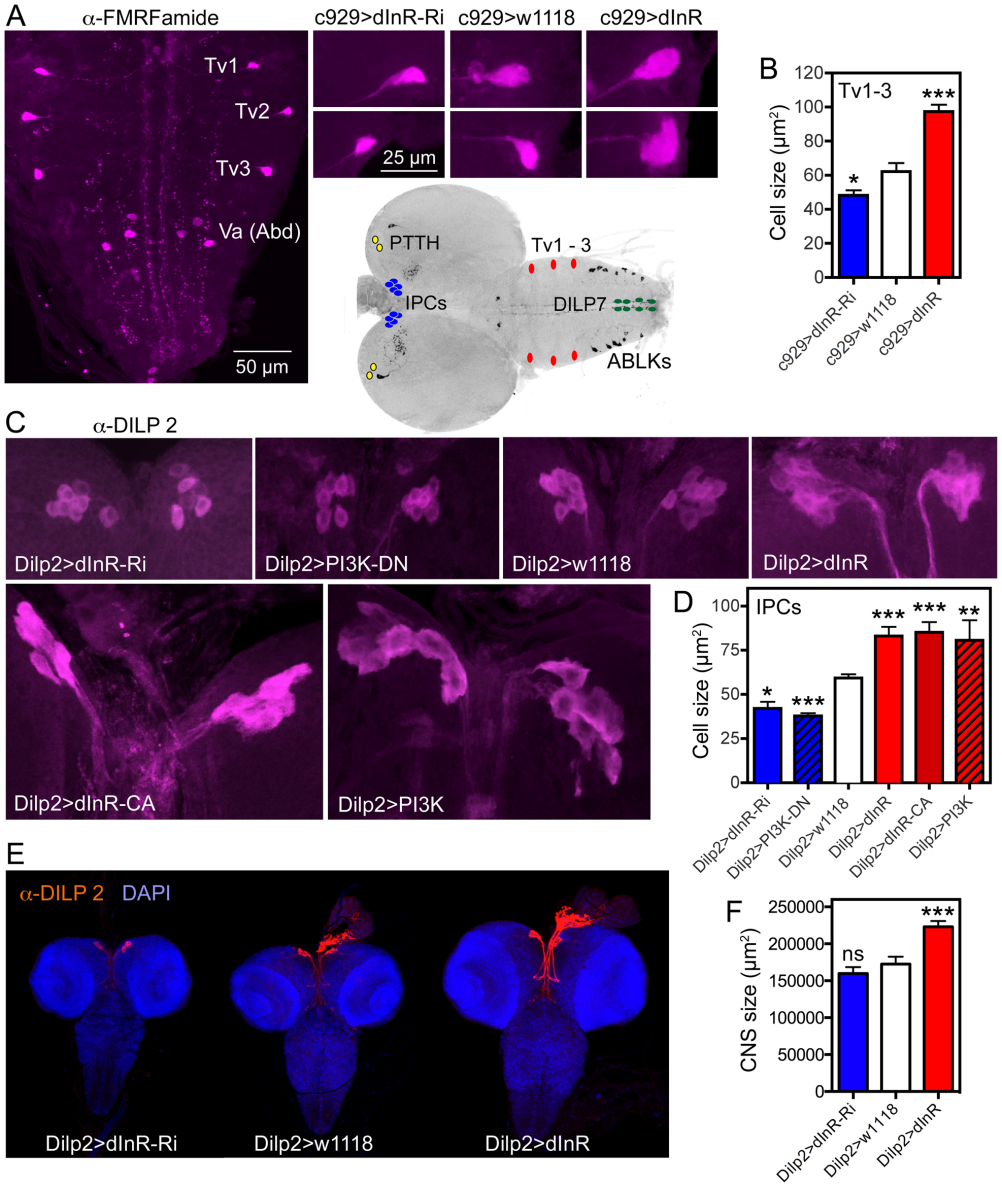
Dimm expressing neurons respond to manipulations of the dInR and PI3K. The inset at the center shows the localization in the CNS of third instar larva of neurons depicted in Fig. 6, 7 and Fig. S11. **A** Using the c929-Gal4 to drive dInR constructs, and an antiserum to FMRFamide, we monitored the cell body size of the thoracic Tv1 – 3 neurons, known to express Dimm. As seen in the smaller panels the cell bodies are smaller after dInR-RNAi and larger after dInR over expression. **B** Quantification of Tv1–3 cell body sizes (*p<0.05, ***p<0.001, n = 6–8 animals for each genotype from 3 crosses; unpaired Student's T-test). **C** and **D** Also the cell bodies of the insulin producing cells (IPCs) of the larval brain respond significantly to manipulations of the dInR as well as PI3K. A dominant negative form of PI3K (PI3K-DN) was used. **D** Quantification of cell body sizes of IPCs (*p<0.05, **p<0.01, ***p<0.001, n = 6–16 animals for each genotype from 3 crosses; unpaired Student's T-test). **E** Overexpression of dInR on IPCs also affect IIS and has systemic effects on growth of the CNS. **F** Quantification of the dInR mediated growth of the CNS. Only overexpression of dInR produces a significant change of CNS size (***p<0.001; unpaired Student's T-test; n = 10–12 animals for each genotype, from 3 crosses).

We showed already that c929-driven manipulations of dInR affected cell size in ABLK neurons (Fig. S3F–I). Next, we monitored the effect on the cell bodies of c929/DIMM neurons expressing the peptide FMRFamide. Among these are three pairs of large ventral FMRFamide-producing neurosecretory cells (Tv1–3) in the thoracic ganglia [29], [66]. Over expression of the dInR increases the size of the cell bodies of the Tv1–3 neurons and dInR-RNAi leads to a slight, but significant decrease (Fig. 7A,B, Table 2).

Another group of neurosecretory cells that express DIMM are the 14 insulin producing cells (IPCs) in the brain [29]. By manipulating the dInR levels in IPCs with a *Dilp2*-Gal4 driver we found that over-expression of both *dInR* and *dInR^CA^* leads to an increase in cell body size and *dInR*-RNAi to a reduction of cell body size both in larval and adult brains (Fig. 7C,D, Fig. S9, Table 1). Also PI3K manipulations in the IPCs produced similar growth effects both in larvae (Fig. 7C,D, Table 1) and adults (Fig. S9). Here we also noted that *Dilp2*-Gal4-driven dInR in IPCs led to an increase in the size of the entire larval CNS (Fig. 7E, F), suggesting that systemic insulin signaling was affected. Feedback actions of DILPs onto the IPCs that affect IIS have been demonstrated previously [67].

About 20 neurons in the abdominal ganglia express DILP7 as well as DIMM [68]. We used a *Dilp7*-Gal4 to alter dInR expression in these neurons and monitored the cell body size with a DILP7 antiserum in late third instar larvae. We focused on a set of 5 pairs of median neurons in abdominal neuromeres A1 and A6–9 with large cell bodies that were easy to identify individually. The cell bodies increased in size after dInR over expression and were slightly smaller after dInR-RNAi (Fig. S10A–D, Table 2). Especially the dorsal pair (DP neurons) in A1, known to send axons to the pars intercerebralis of the brain, was strongly affected; the more posterior ones in A8–9 that send axons to the intestine, were less, but significantly, affected (Fig. S10A–D).

Finally, using a *Pdf*-Gal4 we found that two subsets of the pigment-dispersing factor (PDF) expressing neurons in the larval abdominal ganglion and in the adult brain known to express DIMM [29] display size phenotypes after dInR manipulations. A set of six abdominal PDF and DIMM-expressing neurosecretory cells in the larva respond to dInR alterations (Fig. S10E, G, Table 2). In the adult brain the cell bodies of the PDF and DIMM-expressing clock neurons designated large LN_v_s (l-LN_v_s) respond to dInR manipulations by size changes (Fig. S10F, G, Table 2), but the DIMM-negative small LNvs (s-LN_v_s), present both in larval and adult brains, do not (not shown).

In summary, our data so far, indicate that peptidergic neurons that express DIMM are affected by targeted manipulations of the dInR during development.

### The size of DIMM-negative neuroendocrine cells is not influenced by dInR, but by PI3K and Rheb

**Figure 8:**
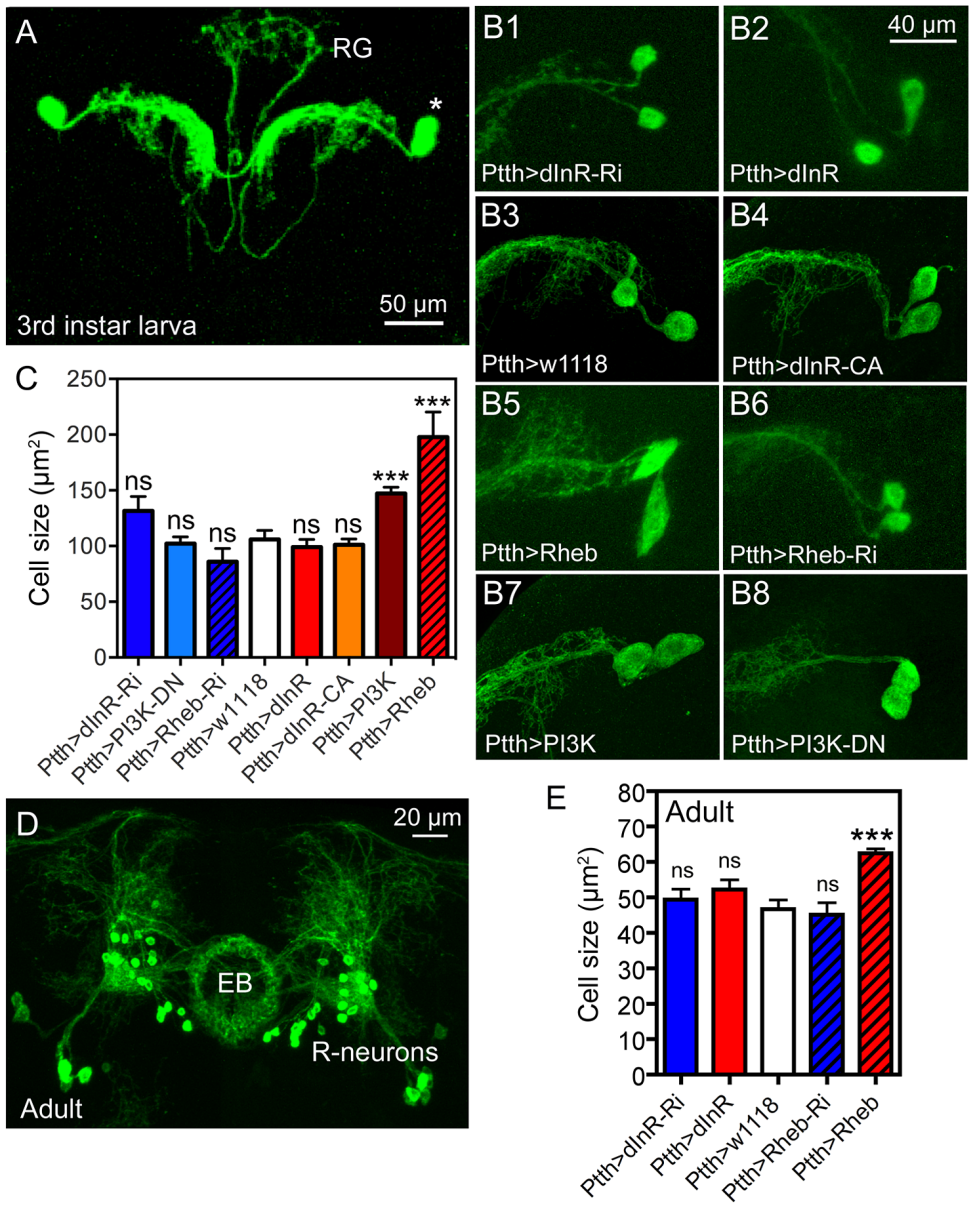
Dimm-negative neurosecretory cells are not affected by dInR manipulations, but by Rheb and PI3K overexpression. **A** Two pairs of larval PTTH neurons displayed with *Ptth*-Gal4-driven GFP. These lateral neurosecretory cells (LNCs; asterisk) supply axon terminations to the ring gland (RG). **B** and **C** Effects of manipulations of dInR, Rheb and PI3K on PTTH neurons of third instar larvae. Neurons are displayed with recombinant *Ptth*Gal4;*gfp*. Rheb (**B5**) and PI3K (**B7**) over expression leads to a size increase, whereas dInR and dInR-CA expression and RNAi and dominant negative (DN) manipulations have no significant effects. **C** Quantification of cell body size reveals that only Rheb and PI3K overexpression increases cell body size (**p<0.01, n = 5–15 animals for each genotype from 3 crosses; unpaired Student's T-test. **D** In the adult brain the PTTH-expressing LNCs undergo apoptosis and instead a subset of the ellipsoid body (EB) R-neurons display GFP. **E** Again, only the Rheb over expression leads to increased cell body size in R-neurons (***p<0.001, n = 5–10 animals for each genotype from 3 crosses; unpaired Student's T-test). CA, constitutively active; DN, dominant negative.

To test the requirement of DIMM in regulation of cell size in large peptidergic neuroendocrine cells we analyzed a set of 4 large neurosecretory cells producing the peptide prothoracicotropic hormone (PTTH). These neurons are functional only in the larva [69] and are known to be DIMM-negative [29]. We expressed *dInR*, *dInR^CA^* and *dInR*-RNAi under the influence of a *Ptth*-Gal4 and monitored cell body size of the neurons in the late third instar larvae. The size was determined after anti-GFP labeling of *Ptth*-Gal4;UAS-*gfp*-driven dInR manipulations and was not affected (Fig. 8A–C, Table 1). Using the *Ptth*-Gal4 we then drove *Rheb* and *Rheb*-RNAi in the PTTH neurons. Here we noted a significant increase in cell body size with over expression of *Rheb*, but similar to the *Lk*-Gal4 experiments in larvae there was no effect of *Rheb*-RNAi (Fig. 8B, C, Table 1). Also PI3K overexpression induces enlarged cell bodies of PTTH neurons, but there was no effect of PI3K^DN^ (Fig. 8B, C; Table 1). Growth effects of PI3K have been demonstrated in interneurons previously and it was proposed that tyrosine kinases other than the dInR could be upstream regulators [45], [63], [70]. Hence, we show that the cell size of DIMM negative neurons can be increased, but not via the dInR.

The PTTH neurons undergo apoptosis in the early adult fly and curiously in the older fly the *Ptth*-Gal4 expression is seen in R-neurons that supply processes to the ellipsoid body of the central complex [69]. We analyzed the cell body size of these R-neurons in older flies and found that again only Rheb over expression increased the cell size (Fig. 8D, E, Table 1). This is consistent with the effect of Rheb of cell size in DIMM negative mushroom body neurons [44].

### Origin of DILPs that regulate the size of peptidergic neurons

There are several possible sources of DILPs that could affect growth of DIMM-expressing peptidergic neurons in early larvae: DILP2, 3 and 5 released from IPCs or DILP6 from fat body could act via the circulation, DILP7 could act in a paracrine fashion from abdominal neurons with central branches (Fig. S11A), or DILP2 and 6 released from glial cells during early larval stages [7], [21], [68], [71] (see Fig. S12). Perhaps even the newly discovered DILP8 released from imaginal discs could act via the circulation [72], [73].

We tested two of these alternatives experimentally. To investigate the possible roles of brain IPCs and abdominal DILP7-producing neurons on LK neuron size we inactivated either of these insulin-producing neuron groups by expressing the hyperpolarizing potassium channel, dOrk1 [54] under control of *Dilp2* or *Dilp7*-gal4 drivers. We measured ABLK cell bodies in third instar larvae and found that hyperpolarization of IPCs led to slightly, but significantly, decreased ABLK cell bodies, whereas the same manipulation of DILP7 neurons produced no phenotypes (Fig. S11B–F). Also ablation of the IPCs by means of *Dilp2*-Gal4 driven *reaper* reduced the size of the ABLKs (Fig. S11D). Thus DILPs from IPCs, but not DILP7, could play a role in regulation of ABLK neuron size. Not surprisingly, we found that this may be due to a general effect on somatic growth and the Dilp2>Ork larvae displayed smaller volume of the CNS and adults had smaller body and wing sizes (Fig. S11F, H–J). Furthermore, as described in a previous section we also found that the size increase in IPC cell bodies after dInR over expression was accompanied by an increase in over-all CNS size (see Fig. 7E, F). As seen in Fig. S11D and F the ablation or inactivation of IPCs does not cause a drastic decrease in ABLK size. Probably this is due to redundant sources of DILPs such as DILP6 released from the fat body or glial cells in earlier larval stages.

In 1^st^ and 2^nd^ instar larvae DILPs released from specific glial cells in thoracic neuromeres of the ventral ganglion terminate cell cycle arrest and growth in neuroblasts [21]. Glial cells may therefore be a source of DILPs also for neuronal growth. We confirm here that *Dilp6*-Gal4 driven GFP is prominent also in glial cells adjacent to ABLK neurons and elsewhere in abdominal ganglia in 1^st^ and 2^nd^ instar larvae (Fig. S12) and we demonstrated that dInR over expression induces strong increase in ABLK cell body size already in 1^st^ instar larvae (Fig. 2J–M). Our findings suggest DILPs from the IPCs, but not DILP7 from abdominal neurons, are likely to be important for size regulation, and we cannot exclude a role of DILP6 from glial cells in earlier growth.

### Dimm-RNAi in ABLK neurons affects cell size and dInR expression, and dInR-RNAi affects DIMM levels

**Figure 9:**
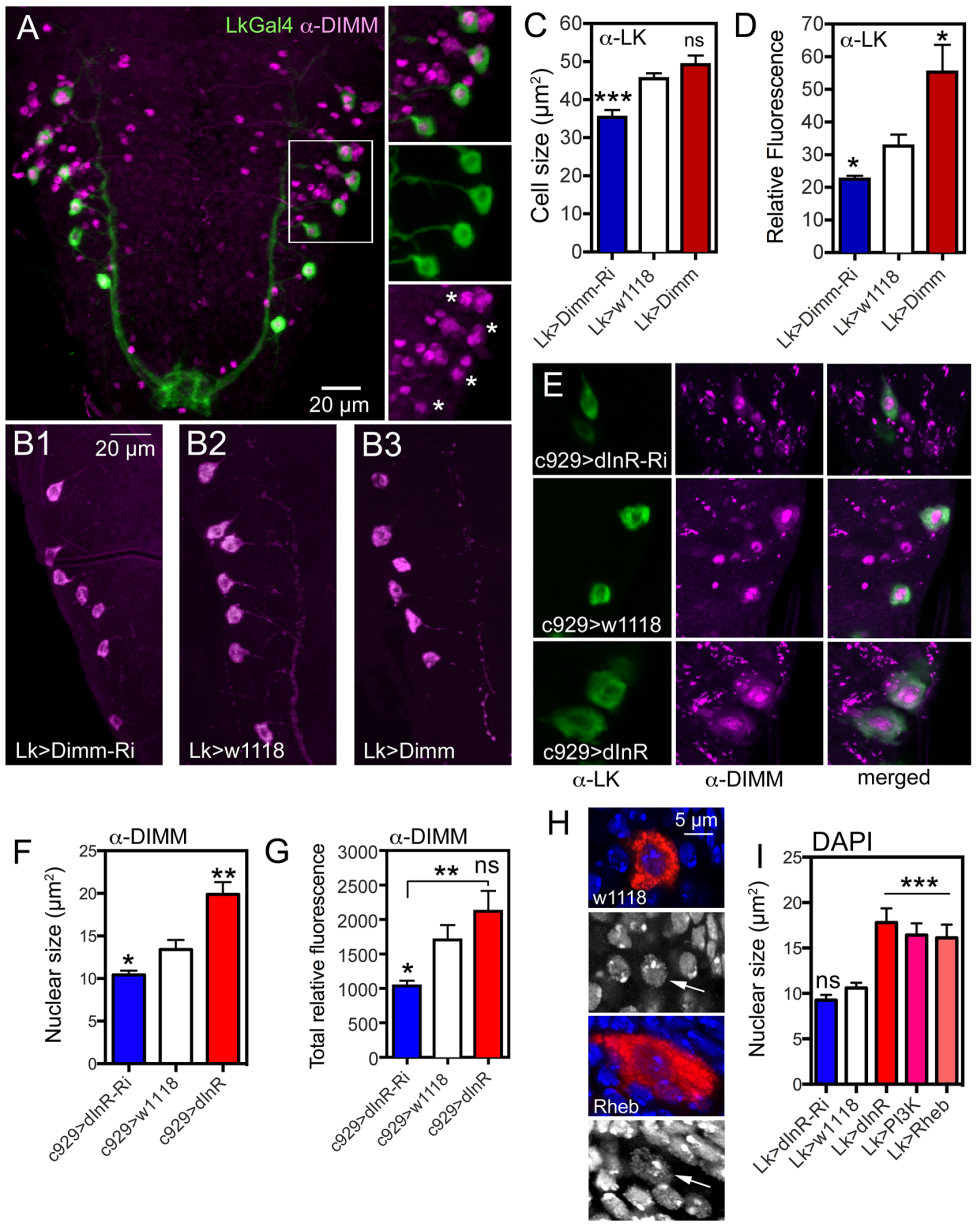
Possible interactions between *Dimm* and *dInR* in ABLKs and effects on nuclear size. **A**. The ABLKs (*Lk*-Gal4-GFP; green) express DIMM-immunolabeling (magenta) in nuclei. Details of the framed area are shown in smaller panels (asterisks indicate nuclei of LK neurons). **B–C** The size of LK cell bodies diminishes with *Lk*-Ga4 driven *Dimm*-RNAi, but is not affected by *Dimm* overexpression (***p<0.001, n = 6–9 animals from 3 crosses for each genotype; unpaired Student's T-test). **D** The LK-immunofluorescence in ABLKs is affected by both Dimm-RNAi and over expression (*p<0.05, n = 6–9 flies for each genotype from 3 crosses; unpaired Student's T-test). **E** and **F** Manipulations of dInR using the c929-Gal4 affects nuclear size as determined by DIMM immunolabeling (*p<0.05, **p<0.01, n = 7–8 animals for each genotype from 3 crosses: unpaired Student's T-test). The surface area of each DIMM-labeled nucleus in LK-immunolabeled cell bodies (in A1–A4 segments) was measured using Image J and the average nuclear size (LK-neurons in A1–A4) of each fly was thereafter determined. **G** The total fluorescence (mean fluorescence multiplied by cell size) in abdominal DIMM immunolabeled neurons decreases with dInR-RNAi, but there is no significant increase after dInR over expression (*p<0.05, **p<0.01, n = 8 animals for each genotype from 3 crosses; unpaired Student's T-test). **H** and **I** Effects of dInR, PI3K and Rheb manipulations on size of nuclei in ABLKs. In **H** nuclear size is visualized by DAPI staining (blue) in ABLK neurons (red). Nuclear size (arrows) is enlarged after Rheb over expression. **I** Quantification of nuclear size shows that dInR-RNAi has no effect (compared to controls), whereas over expression of dInR, PI3K and Rheb produces a significant enlargement of nuclei (unpaired Student's T-test, *** p<0.001, n = 6–11 animals for each genotype from 3 crosses).

DIMM is expressed in a large subpopulation of the peptidergic neurons of *Drosophila*, including ABLKs (Fig. 9A) and is required for activation of key genes involved in differentiation of a dynamic secretory phenotype [26], [28], [29]. Since only DIMM neurons displayed size phenotypes after dInR manipulation, we asked whether there is a functional correlation between DIMM and dInR-regulated neuronal development and growth.

To test this we knocked down or overexpressed *Dimm* with the *Lk*-Gal4 driver and monitored ABLK cell size. These cell bodies were smaller after *Dimm*-RNAi, but did not increase in size after over expression compared to controls (Fig. 9B, C). As shown earlier, the LK-immunoreactivity in ABLKs diminished after Dimm knockdown and increased after over expression (Fig. 9D) [65].

We next asked if there is a relation between dInR levels and DIMM expression. To test this we expressed dInR or dInR-RNAi in *Lk*-Gal4 or c929-Gal4 neurons and applied antiserum to DIMM to the larval CNS and monitored the immunofluorescence level in nuclei of the neurons. After dInR-RNAi there was a decrease in total DIMM immunolabeling intensity, as well as in the size of DIMM labeled nuclei (Fig. 9E–G). However after dInR overexpression the nuclear size, as determined by DIMM immunolabeling, increased (Fig. 9F), whereas total DIMM immunolabeling was not significantly affected (Fig. 9G). Thus, there is a weak, but significant effect of dInR on DIMM expression.

**Figure 10:**
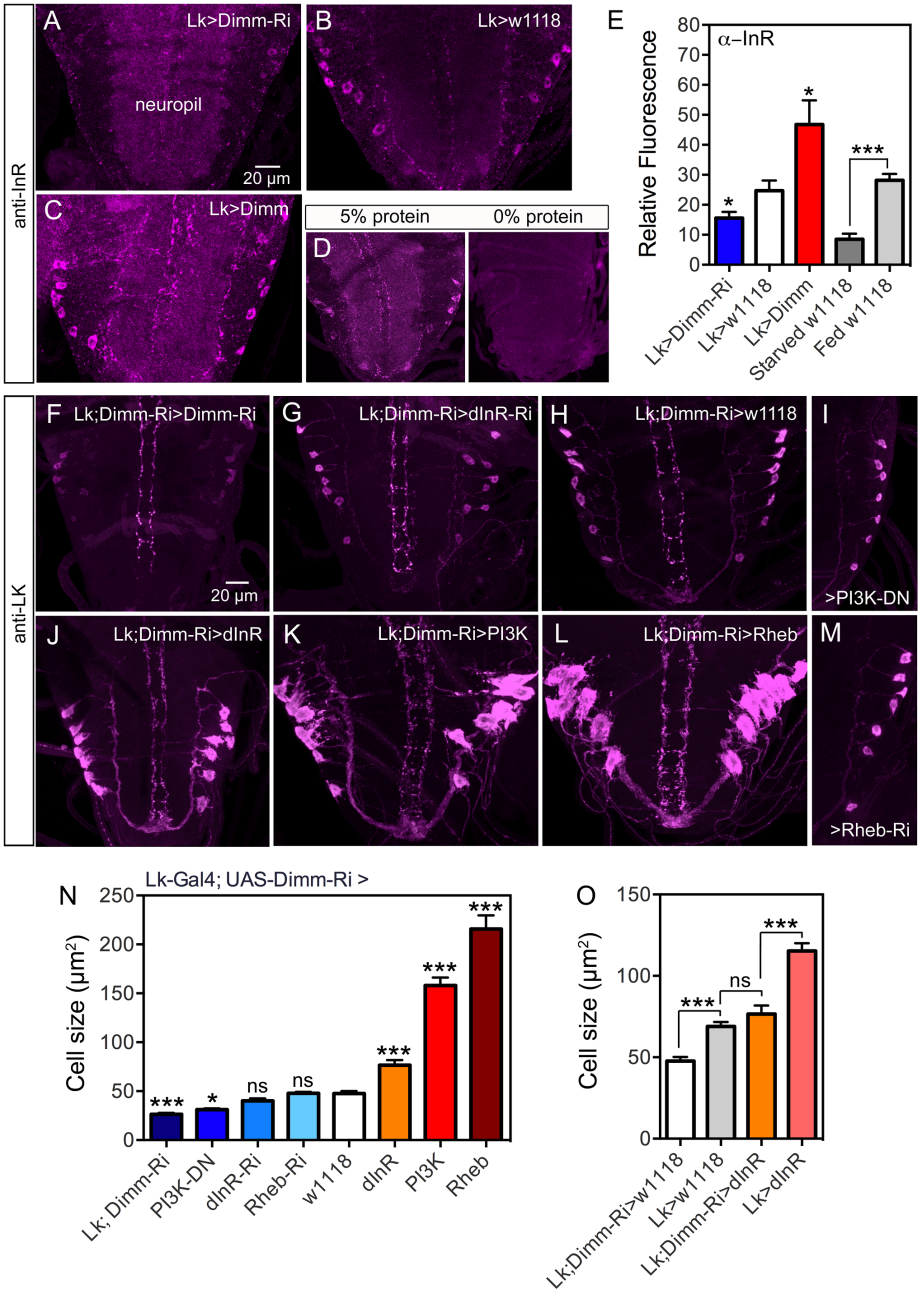
Knockdown of Dimm in ABLKs affects dInR expression and cell size regulation. **A–C** InR immunolabeling (antiserum #3021) after manipulations of *Dimm* levels in LK neurons. Dimm knockdown drastically decreases InR immunolabeling, except in the neuropil (A) compared to controls (B). Overexpression of Dimm in LK neurons increases InR immunolabeling (C). **D** Insulin receptor immunoreactivity is affected by protein diet. Larvae were reared under two conditions: 20% sucrose with 5% or 0% protein (Yeast) according to the schedule in Fig. S15A. InR labeling is far stronger under 5% protein than 0% protein. Not only the ABLKs, but also general neuropil InR immunolabeling is decreased under 0% conditions. **E** Quantification of immunolabeling of ABLK neurons in A–D. The changes in InR immunolabeling are shown in relation to controls (Lk>w1118) (*p<0.05, ***p<0.001, n = 7–16 animals for each genotype from 3 crosses: unpaired Student's T-test). This graph also shows the effect of 48 h protein starvation on InR immunolabeling (grey bars). **F–M** With a double transgene (*Lk*-Gal4;UAS-*Dimm*-RNAi) we analyzed the effects of *Dimm*-RNAi on manipulations of dInR, PI3K and Rheb in ABLKs labeled with anti-LK in late third instar larva. In I and M only half a ganglion is depicted. **N** Quantification of ABLK cell body size in the different genotypes shown in F–M. Larvae with a heterozygous *Dimm*-RNAi genetic background displayed significantly larger ABLK cell size when either of dInR, PI3K or Rheb was overexpressed in LK neurons. Overexpression of dInR induced much weaker cell growth than PI3K or Rheb. ABLKs displayed smaller cell size when Dimm was knocked down with two copies of Dimm-RNAi (*Lk-Gal4;*UAS-*Dimm-RNAi>Lk-Gal4*; UAS-*Dimm-RNAi*). Knockdown of dInR in heterozygous Dimm-RNAi flies lead no decrease of cell size. Statistics: ***p<0.001; ns, not significant; n = 9–11 animals from 3 crosses for each genotype, unpaired Student's T-test; comparisons were made between each of the manipulations and the control (*Lk-Gal4*;UAS-*Dimm-RNAi>*w1118). Genotypes for each bar are as follows. **Lk;DIMM-Ri**, *Lk-Gal4/Lk-Gal4*; UAS-*Dimm-RNAi/*UAS-*Dimm-RNAi*, **PI3K-DN**, *Lk-Gal4/*UAS-*PI3K-DN*; UAS-*Dimm-RNAi/+*, **Rheb-Ri**, *Lk-Gal4/+*; UAS-*Dimm-RNAi/*UAS-*Rheb-RNAi*, **dInR-Ri**, *Lk-Gal4/*UAS-*dInR-RNAi*; UAS-*Dimm-RNAi/+*, **w1118**, *Lk-Gal4/+*; UAS-*Dimm-RNAi/+*, **dInR**, *Lk-Gal4/*UAS-*dInR*; UAS-*Dimm-RNAi/+*, **PI3K**, *Lk-Gal4/*UAS-*PI3K*; UAS-*Dimm-RNAi/+*, **Rheb**, *Lk-Gal4/*UAS-*Rheb*; UAS-*Dimm-RNAi/+*. **O** Diminished *Dimm* in ABLK neurons significantly restricted dInR-induced cell growth. Overexpression of dInR in *Dimm*-RNAi flies (Lk;Dimm-RNAi>dInR) resulted in cell size similar to controls (Lk>w1118), and much smaller than after overexpressing dInR alone. (***p<0.001; ns, not significant; n = 6–13 animals for each genotype from 3 crosses; unpaired Student's T-test). Genotypes for each bar are as follows. **Lk;DIMM-Ri>w1118**, *Lk-Gal4/+*;UAS-*Dimm-RNAi/+*, **Lk>w1118**, *Lk-Gal4/+; +/+*, **Lk;Dimm-Ri>dInR**, *Lk-Gal4/*UAS-*dInR*;UAS-*Dimm-RNAi/+*, **Lk>dInR**, *Lk-Gal4/UAS-dInR; +/+*.

To test whether *Dimm* influences dInR expression levels in ABLK neurons we knocked down or overexpressed Dimm with the *Lk*-Gal4 and measured dInR immunolabeling. We found that Dimm-RNAi diminished dInR labeling in ABLKs, but not generally in neuropil, whereas overexpression resulted in significantly increased dInR (Fig. 10 A–C, E). We also measured dInR labeling in third instar larvae that had been kept for 48 h with 0% protein in the diet compared to 5% protein (Fig. 10D; Fig. S15A). There was a significant difference in dInR immunolabeling levels between these two treatments (Fig. 10D, E).

To further examine the requirement of DIMM expression for dInR-mediated size regulation we employed a double transgene (*Lk-*Gal4*/Lk-*Gal4;UAS-*Dimm-*RNAi*/*UAS-*Dimm-*RNAi; here abbreviated *Lk*-Gal4;*Dimm*-Ri) for interfering with *dInR*, *PI3K*, *Rheb* and *Dimm* in ABLKs. We find that flies bearing the homozygous double transgene *Lk*-Gal4;*Dimm*-Ri display very small ABLKs (Fig. 10F, N). However, driving the *dInR*-RNAi with *Lk*-Gal4;*Dimm*-Ri does not significantly diminish cell bodies compared to controls (Fig. 10G, H, N) and overexpression of the dInR in *Dimm* knockdown flies results in less increase of cell size (Fig. 10J,N). Over expression of *PI3K* and *Rheb* with the *Lk*-Gal4;*Dimm*-Ri driver on the other hand leads to a substantial size increase (Fig. 10K,L,N). Dominant negative PI3K expression decreases cell body size significantly (Fig. 10I, N), but not Rheb-RNAi (Fig. 10M, N). As discussed for PTTH neurons above, we propose that PI3K and Rheb may act independently of dInR activation. Compared to *Lk*-Gal4>*dInR* and *Lk*-Gal4>*w^1118^* controls, the *Lk*-Gal4;*Dimm*-Ri>*dInR* flies display ABLKs that did not grow significantly (Fig. 10O), suggesting that after *Dimm* knockdown the *dInR* has little effect on cell size. In Fig. 10O it can also be seen that *Dimm*-RNAi (Lk;Dimm-Ri>w^1118^) has an effect on cell size compared to Lk>w^1118^ suggesting that *Dimm* expression alone affects cell size.

### The dInR-mediated size increase affects Golgi apparatus and nuclear size

Since DIMM expression correlates with competence to produce neuropeptides in bulk we monitored the effect of dInR manipulations on cell organelles involved in protein synthesis, such as the trans-Golgi network from which secretory vesicles arise. One marker that has been associated with the trans-Golgi network in *Drosophila* neurons is PICK1 (the protein interacting with C kinase 1) a Bar domain protein that is known to be expressed in DIMM expressing neurons [74], [75]. We therefore employed antiserum to PICK1 to obtain an estimate of the amount of Golgi and likely secretory activity in ABLKs after dInR manipulations (using the c929-Gal4). We found that dInR over expression increases both the PICK1-immunolabeled area within the cell body and the total relative PICK1 immunofluorescence in the abdominal ganglion neurons (Fig. S13A–E). Since the punctate PICK1 immunolabeling approximately represents the extent of the trans-Golgi [74], [75] (see also Fig. S13F) we suggest that dInR over expression leads to an increased capacity for secretory vesicle production.

Using neuropeptide immunolabeling the nuclei of neurons commonly remain unlabeled and their size can be estimated. A survey of nuclei in ABLKs and IPCs (e. g. Fig. 2A–D, 4, 7C) suggest that their size increases after dInR over expression. Also DIMM immunolabeling indicated nuclear growth after IIS manipulations (Fig. 9E, F). Thus we wanted to test whether the nuclear size increase is due to DNA-endoreplication, as proposed for giant neurons in mollusks [27], [76]. Endoreplication entails DNA synthesis in absence of cell division and leads to increased cell size and either polyploidy or replication of some genomic regions, polygeny [77]. Increases in DNA ploidy can be induced by growth and IIS/TOR signaling in endoreplicating tissues [47], [78]. To obtain a measure of nuclear size, as well as estimate of DNA content, we applied DAPI staining (see [27], [44]) after dInR, PI3K and Rheb manipulations or LK neurons. We found significant increases in the size of DAPI labeled nuclei in ABLK neurons after over expression of these components, but no change in size after dInR-RNAi (Fig. 9H,I). Also in IPCs the nuclei were larger after dInR over expression (Fig. S14A–D). We quantified the DAPI fluorescence but there was no significant increase in fluorescence in nuclei of ABLKs or IPCs (Fig. 9H, Fig. S14E). Thus, there is probably no induction of DNA-endoreplication by increased IIS and TOR activity in these neuron types in the larvae.

### Size scaling in *Dimm* positive neurons is nutrient dependent

To test whether the *Dimm*-positive neurons are affected by nutrient restriction or increased protein content in the diet we conducted a set of experiments. For nutrient restriction we transferred second instar larvae of different genotypes from food with 20% sucrose and 5% protein to food containing only 20% sucrose (0% protein). Another set of larvae was raised on food containing 20% sucrose and 20% protein. Controls were raised on 20% sucrose and 5% protein throughout (see Fig. S15A for diet regime). The cell body size of ABLKs was measured in late third instar larvae along with the size of abdominal serotonin-immunoreactive neurons (5-HT neurons) as a reference. We used larvae of the genotypes Lk>dInR-RNAi, Lk>w^1118^ and Lk>dInR.

**Figure 11:**
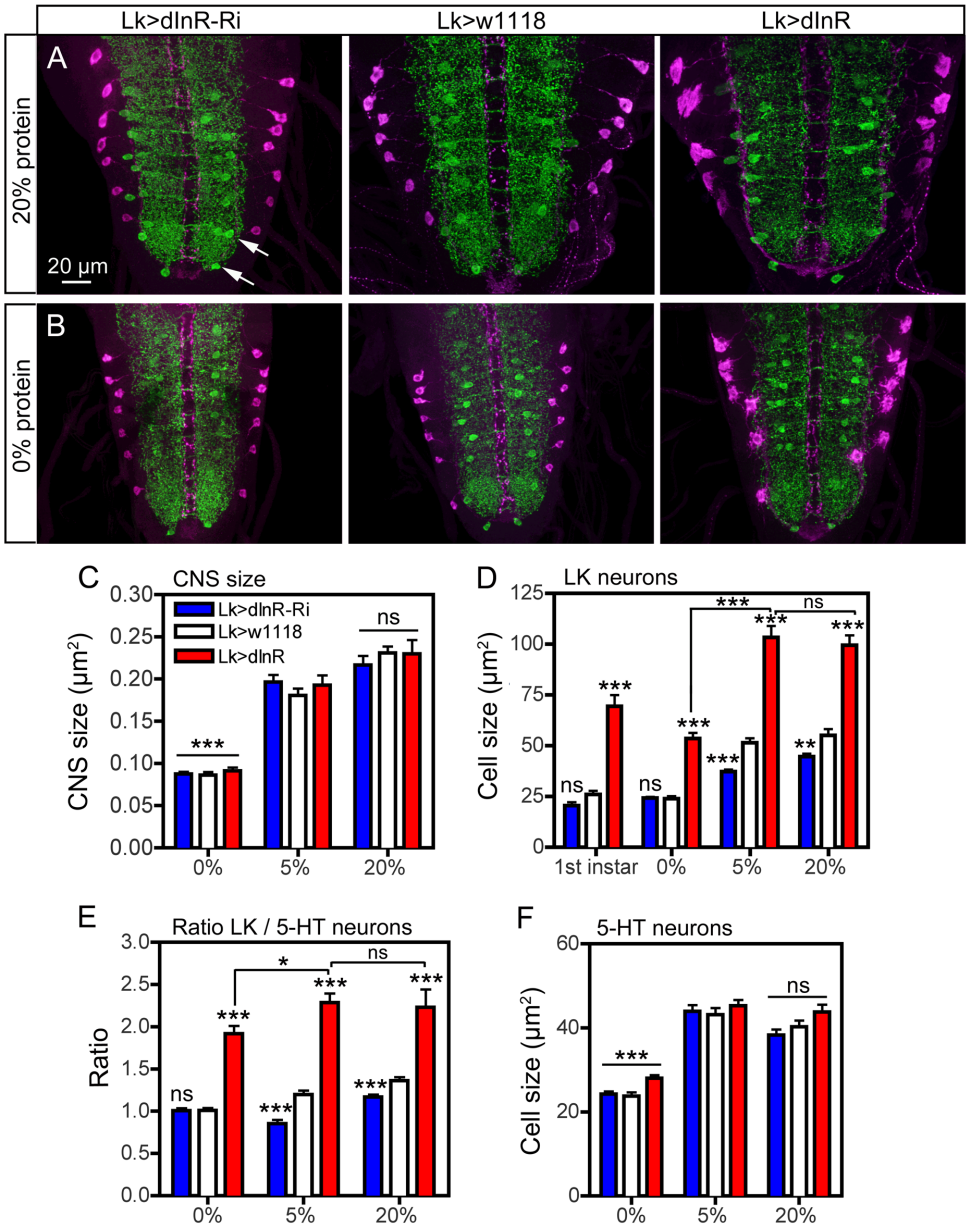
Effects of diet on size of ABLK neurons. Larvae of different genotypes were reared on three different diets: 20% sucrose with either 0%, 5% or 20% protein (yeast) as detailed in the scheme in Fig. S15A. Late third instar larvae were labeled with anti-LK (magenta) and anti-5-HT (green). Statistics: *p<0.05, **p<0.01, ***p<0.001, n = 6–12 larvae for each genotype from 3 crosses; unpaired Student's T-test for comparison of genotypes; Two-way Anovas for comparisons of diet effects). **A** and **B** Manipulation of dInR expression in LK neurons affects the size of ABLK neurons in larvae fed protein-rich (20%; A) and protein-free (0%; B) and normal (5%; not shown) diet. Cell bodies of 5-HT immunolabeled neurons (arrows in Fig. 11 A) are used for reference. The size changes in CNS and different neurons are quantified in Fig. 11 C–F (cell bodies are shown in higher magnification in Fig. S15 B). **C** The low protein (0%) diet significantly (p<0.001) affects the size of the entire CNS compared to “standard diet” (5%), whereas 20% protein does not increase the CNS (ns; p>0.05). Note that the CNS size is not affected by the dInR manipulations. **D** Quantification of ABLK cell body size in third instar larvae fed the three different diets, compared to those in first instar larvae (genotypes as in C). Under 0% protein conditions all genotypes display third instar ABLK cells with sizes corresponding to those in first instar larvae. These are significantly smaller than in flies fed 5% and 20% protein (p<0.001 for all genotypes compared for 0% and 5% protein; Two-way Anova). Larvae fed protein-rich food (20%) do not display increased ABLK cell bodies compared to those fed 5% protein. The significance values indicate differences to control flies (Lk>w1118) for each protein condition (*p<0.05, **p<0.01, ***p<0.001, ns, not significant, n = 6–10 larvae for each genotype from 3 crosses; unpaired Student's T-test) and comparisons between protein diets for dInR overexpression (Two-way Anova). **E** The ratio between the ABLK cell size and the size of 5-HT neuron cell bodies in corresponding abdominal neuromeres (same protein conditions and genotypes as in C). These were determined to establish whether the ABLK size is affected disproportionally by protein diet (the size of the 5-HT neuron cell bodies for the three protein diets are shown in **F**). It can be seen that with 5-HT cells as size reference the dInR overexpression only marginally (p<0.05; Two-way Anova) increased the ABLKs after dInR overexpression when comparing 0% and 5% (and 20%) protein. For each diet dInR over expression significantly increases the ratio between ABLK and 5-HT cell body sizes compared to control flies. Only small (but significant) effects can be seen after dInR-RNAi in 5% and 20% diets. **F** Size of cell bodies of 5-HT immunolabeled neurons in abdominal ganglia in larvae reared in the three different diets (genotypes as in C). Note that manipulations of the dInR in LK neurons do not affect the 5-HT neurons, but 0% protein diet results in significantly smaller cell bodies compared to 5% and 20% protein.

We found that under nutrient restriction the entire CNS is significantly smaller in larvae, of all genotypes, as compared to those fed 5% or 20% protein (Fig. 11C). The size of the ABLK cell bodies was also significantly smaller for the three genotypes after 0% protein diet, compared to the other feeding conditions (Fig. 11A,B,D; Fig. S15B). In fact, the cell body sizes resembled those seen for the same genotypes in the late first instar larva (Fig. 11D). Feeding larvae protein-rich diet did not increase CNS size or ABLK cell body size compared to 5% protein for any genotype (Fig. 10C,D; Fig. S15C,D). We calculated the ratio between cell body size of 5-HT neurons and ABLKs to determine relative growth of dInR manipulated versus non-manipulated neurons. It was seen that the ratio only marginally (but significantly) increased from 0% to 5% protein after dInR overexpression in LK neurons (Fig. 11E). It can be noted that not only the CNS but also the 5-HT neurons are significantly smaller after 0% protein diet compared to 5 and 20% (Fig. 10F). In summary we show that the dInR-induced growth is protein dependent.

## Discussion

Our study shows that insulin receptor-mediated signaling can selectively regulate the size of peptidergic neuroendocrine cells during development and in the adult organism. This may provide a mechanism for plasticity in size of secretory neurons independent on growth of other neurons, since we found that motor neurons and several types of interneurons do not display this feature. The dInR-mediated growth regulation seems to require properties mediated by, or coincident with, the expression of the transcription factor DIMM (summarized in Tables 1 and 2). Thus, several subpopulations of the approximately 300 DIMM-expressing neurons were found to respond to manipulations of dInR levels or signaling components downstream to this receptor. We also found that dInR manipulations are less effective when *Dimm* levels are knocked down. DIMM-expressing neurons are known to episodically release large amounts of amidated neuropeptide or peptide hormone through the regulated secretory pathway, and are thus equipped with a sizable protein synthesis machinery in the cell body [25], [26], [29], [30]. It has been proposed that secretory cells have their regulated secretory machinery scaled up during differentiation to be fit for their demanding peptide signaling and plasticity in function related to changes in environment and organismal homeostasis later in life [25]. Hence, DIMM, and MIST1 in vertebrates, are transcription factors that orchestrate the up-scaling of the secretory pathway and, thus, the size of the cell bodies [25]. A large number of genes are transcriptional targets of DIMM, but insulin-signaling components are not prominent among genes affected by over-expression of DIMM [26]. Therefore, the activation of the dInR may trigger signaling that converges on scaling events in the DIMM expressing neurons.

An extensive scaling of neuron size has been recorded in gastropod snails where many of the peptidergic neurosecretory cells are gigantic [76]. A recent study further demonstrated that the size of these peptidergic neurons increases as the adult animal grows in response to rich nutrition [27]. This growth depends on DNA-endoreplication in the specific neurons, where genomic multiplication ensures efficient protein synthesis. It was proposed that efferent neurons and neurosecretory cells, but not interneurons, adapt their sizes to the enlarged periphery and circulation as the animal grows [27]. Our results suggest that targeted increases in IIS results in larger nuclei, but not significantly increased DNA levels, as measured by DAPI labeling in nuclei. Thus up-regulated IIS probably does not induce increases in DNA ploidy in the DIMM-positive neurons. This is expected since although many cell types in *Drosophila* larvae are polyploid, there are no reports on endoreplication in neurons [47], [78], [79]. However, other mechanisms may regulate the size of the nucleus. For instance the gene *kugelkern* (*kuk*) affects growth of the nuclear envelope, and it is generally presumed that larger cells have larger nuclei (see [80], [81]).

In mammals brain growth depends on IGF signaling during precise time windows during development, and relies on ubiquitous neuronal expression of the IGF1 receptor [10], [82], [83]. IGF signaling, as well as insulin signaling, also partake in other developmental processes in the CNS and may do so in concert with other factors as part of a combinatorial instructive signal [5]. In *Drosophila* growth mediated by IIS has primarily been studied at the whole organism or tissue/organ level [3], [6], [7], [11], [16], [71], [84]. During development the IIS may act on both cell growth and proliferation. This has been shown for *Drosophila* neuroblasts where developmental quiescence is terminated by IIS and cell proliferation starts after neuroblast growth [21]–[23]. There seems to be no reports on IIS-mediated post-mitotic growth of neurons at the single cell level in *Drosophila*, although the effects of PI3K and TSC2 (*gigas*) on synaptogenesis and neuropil growth has been documented after targeting specific sets of neurons [45], [63].

During feeding stages post-mitotically growing neurons (as a tissue) are regulated by the cell autonomous nutrient sensing TOR signaling pathway combined with IIS [6], [15]–[17], [24], [47] (Fig. S16). In case of nutritional deficiency during development the growth of CNS neuroblasts is spared, whereas many other cell types respond to malnutrition by restricted growth [17], [85]. This sparing depends on a pathway employing Alk and its ligand jelly belly [17]. At nutritional restriction Alk signaling utilizes some of the same downstream components as the dInR mediated IIS (see Fig. S16), but is independent of TOR signaling [17].

The sets of DIMM-expressing neuroendocrine cells studied here appear to have another layer of growth control. This may enable enlargement of their cell bodies and neurites during feeding stages to adapt to somatic growth of the animal during rich nutritional conditions, or spare their growth during nutrient restriction. This regulation depends on IIS through the evolutionarily conserved pathway including dInR, the insulin receptor substrate Chico, PI3K and AKT1 [6], [7], [14], [21]. We also found that the dInR-mediate size regulation is especially prominent during nutritional restriction. Thus, we propose that post-mitotic DIMM-positive neurons are protected by the dInR in a manner resembling the action of Alk signaling in neuroblasts [17]. The size of the DIMM-positive neurons can also be altered by manipulations of TOR signaling components such as TOR, Rheb and S6K/4EBP [6], [15], [44], [46]. DIMM-negative neurons tested display increased cell body size after overexpression of Rheb but not dInR, suggesting a non-IIS dependent growth regulation. There is a previous report that targeted Rheb overexpression induces growth of IPCs and mushroom body Kenyon cells, and thus the entire mushroom body lobes [44]. Furthermore, Rheb over expression in *Drosophila* motor neurons increases growth of axon terminations in neuromuscular junctions, NMJs [62]. This Rheb action could occur either downstream of the dInR, or independent of the dInR as part of the TOR pathway [46], [47] (see Fig. S16). The Rheb-regulated growth of the NMJ and Kenyon cells is likely to be independent of bona fide dInR signaling since we found no dInR-mediated growth phenotypes in motor neurons and a recent paper reported that diminishment of insulin signaling to the Kenyon cells by expression of dInR^DN^ does not induce any overt alteration of mushroom body size or morphology [86]. Thus, it is likely that the Rheb-mediated growth in DIMM negative neurons shown in the present study is independent of dInR.

Our data suggest that PI3K overexpression can increase the size of DIMM-negative neurons (PTTH neurons) and in ABLKs after Dimm knockdown. Since these neurons do not grow in response to dInR expression PI3K could possibly be activated by other upstream elements than the dInR. It has indeed been proposed that PI3K-induced growth of brain interneurons (and synapse formation) could be triggered by tyrosine kinases other than the dInR [45], [63], [70].

The source of the insulin signal that activates growth of DIMM neurons could be either globally released DILPs from the IPCs of the brain (or the fat body) or DILPs released from the local niche in a paracrine fashion. During early larval development surface glial cells in the ventral nerve cord express DILP2 and 6 [21]. These glial cells were shown to release DILPs in response to nutritional signals from the fat body and the paracrine DILPs activate IIS in dormant neuroblasts to enable reactivation of the cell cycle. This exit from dormancy involves cell growth and entry into S-phase [21], [23]. Thus, the DILP signal that regulates growth of DIMM-expressing neurons might be paracrine and glial derived in earlier larval stages, whereas systemic DILP signaling plays a role in later stages. Systemic DILP signaling is suggestive since we find that the size of DIMM-positive neurons can be affected by dInR activation or inactivation throughout larval and adult life. Also, we saw an effect on size of DIMM-positive neurons after inactivation of the DILP producing IPCs in the brain. Combined our findings suggest that there is no specific time window for the dInR mediated size regulation in DIMM-positive neurons. In this context it is important to note that DIMM-expression begins in the embryo and persists into the adult fly [65]. The level of DIMM-expression could be correlated with the expression of LK and DILP2 peptides and transient over-expression of DIMM in adults resulted in increased peptide levels [65].

The dInR-induced growth of the DIMM-positive LK neurons, that we investigated most thoroughly, includes enlarged cell bodies with increased amount of stored neuropeptide and trans-Golgi, increased nuclear size and more extensive axon terminations with larger boutons. We have no evidence that these growth phenomena are linked to DNA endoreplication. In other larval tissues, such as larval muscles of *Drosophila* dInR/TOR signaling is among the key regulators of endoreplication, along with FOXO and the transcription factor Myc [78]. In *Drosophila* DNA endoreplication has furthermore been shown in cells in epidermis, muscle, salivary glands and fat body, as well as in certain large glial cells [78], [79], [87], [88], but to our knowledge not in neurons (see also [44]).

In summary, we propose that major DIMM-positive neuroendocrine cells in *Drosophila* are equipped with a mechanism to maintain their hormone production at an optimum. Selective size scaling of these neurons could adjust hormone production capacity relative to somatic growth and increased demands for peptide signaling. This dInR-dependent regulation is diet-dependent.

## Materials and Methods

### Fly strains and husbandry

All flies (except diet experiments) were reared at 25°C and 12∶12 h light∶dark conditions on a standard yeast, corn meal, agar medium according to the standard medium recipe of Bloomington *Drosophila* Stock Center (BDSC), Bloomington, IN (see http://flystocks.bio.indiana.edu/). Flies with other original genetic backgrounds were backcrossed into *w^1118^* background for four generations before experiments, and *w^1118^* flies were used as controls in all experiments. For experiments in adult flies only males were used (unless specifically stated 3–4 d old adults were used).

The following Gal4 lines were used:

w; *Lk-*Gal4 (II) ([38]; gift from P. Herrero, Madrid, Spain),

w; *Dilp2*-Gal4 (III) ([89] from E. Rulifson, Stanford, CA),

w; *Dilp7*-Gal4 (III) ([90] from Y. N. Jan, San Francisco, CA),

w; *elav*-Gal4/TM6B (III), w; *OK6*-Gal4;UAS-*Myc^Ric^*/TM3Sb (II) ([59] from A. Ferrus, Madrid, Spain),

yw; *pdf*-Gal4 (II) ([91] from J. H. Park, Knoxville, TN),

w; *c929*-Gal4 (III) ([28] from P. H. Taghert, St Louis, MO),

w; *Trh*-Gal4 (II) ([61] from E. A. Kravitz, Boston, MA).

w; *Dilp6*-Gal4 (II) ([21] from A. Brand, Cambridge, UK)

yw, *Dilp6*-Gal4 (X) ([92] from the Drosophila Genetic Resource Center, Kyoto Institute of Technology, Kyoto, Japan; Stock No 103-877).

The following UAS lines were employed:

w; UAS-*dInR*-RNAi (III) [from Vienna *Drosophila* RNAi Center (VDRC), Vienna, Austria]. This line was utilized and characterized previously [41], [42].

w; UAS-*InR^CA^* and yw; UAS-*InR^DN^* ([43] from P. Shen, Athens, GA),

yw; UAS-*dInR* (II), w; UAS-*Akt1*-RNAi (III), w; UAS-*Akt1* (II), w; UAS-*Rheb*-RNAi (III), w; UAS-*Rheb* (II), w; UAS-*S6k.KQ* (II), w; UAS-*S6k.STDETE* (III), w; UAS-*S6k.M* (II), yw;UAS-*Tor.TED* (II), yw;UAS-*NaChBac* (III), w; UAS*-Ork1/*TM6cSb (III), yw;UAS-*mcd8-gfp* (III) and w; UAS-*gfp.nls* (III) [all from Bloomington *Drosophila* Stock Center (BDSC), Bloomington, IN],

w; UAS-*PI3K* and yw; UAS-*PI3K^DN^* (UAS-PI3K92E^D954A^) [[93], [94] from A. Ferrus],

w; UAS-*dInR*-RNAi/CyO (II) and w; UAS-*rpr-hid* (II) ([95] from J. R. Martin, Gif-Sur-Yvette, France),

yw; UAS-*dimm-Myc* (II) and w; UAS-*dimm*-RNAi (II, III) ([28], [96] from P. H. Taghert, St Louis, MO).

yw; *ptth*-Gal4;UAS*-gfp* ([69] from K. F. Rewitz, Copenhagen, Denmark),

w; UAS-*Alk* (III), w; UAS-*Alk^DN^* (III), w; UAS-*Alk^CA^* (II) ([97], [98] from R. Palmer, Umeå, Sweden)

w; UAS-*GRASP-gfp* ([74] from O. Kjaerulff, Copenhagen, Denmark).

Two balancer flies were used:

w; Sco/SM1cy; Dr/TM6BTbSb and w; Sp/Cyo; Vno/TM3Sb (from C. Samakovlis, Stockholm, Sweden).

### Antisera and immunocytochemistry

The central nervous system (CNS) of first, second and third instar larvae, adult CNS, as well as third instar larval body wall muscles were dissected out in 0.1 M sodium phosphate buffer (PB; pH 7.4) and fixed in ice-cold 4% paraformaldehyde (4% PFA) in 0.1 M PB for 2–4 h. All tissues were rinsed with 0.1 M PB three times over 1 hr and washed finally in 0.01 M PBS with 0.25% Triton-X (PBS-Tx) for 15 min before application of primary antisera. The primary antisera were diluted in 0.01 M PBS-Tx with 0.05% sodium azide. Incubation with primary antiserum for whole tissues was performed for 24–48 h at 4°C with gentle agitation. Tissues were rinsed thoroughly with PBS-Tx, followed by application of secondary antibody overnight and thorough wash in 0.01 M PBS and then mounted in 80% glycerol in 0.01 M PBS.

The following primary antisera were used: an antiserum to cockroach leucokinin I (LK I) (1∶2000) [40] known to recognize the conserved C-terminus of insect leucokinin peptides [37], [99] was used for identification and quantification of leucokinin. Rabbit anti-mosquito (*Aedes aegypti*) insulin receptor (anti-Aed InR; raised against 50 amino acids from the extracellular alpha chain), kindly provided by of M. Brown, Athens, GA) at 1∶1000, rabbit anti-phosphorylated human InR (#3021 and #3024, from Cell Signaling Technology) at 1∶2000, rabbit anti-DILP2 (1∶2000) (from J. Veenstra, Bordeaux, France; [100]), rabbit anti-DILP7 (1∶4000) (from I. Miguel-Aliaga, Cambridge, UK; [68]), mouse anti-PDF (1∶80) (C7; Developmental Studies Hybridoma Bank, University of Iowa), rabbit anti-PDH (1∶3000) ([101] from H. Dircksen, Stockholm, Sweden), guinea pig anti-DIMM (1∶2000) (from P. Taghert, St. Louis, MO; [102]), rabbit anti-FMRFamide (1∶4000) (117∶I, from C. Grimmelikhuijzen, Copenhagen, Denmark; [103]), rabbit anti-vGluT (1∶1000) (from H. Aberle, Münster, Germany [104]), rabbit anti-PICK1 (1∶500) (from O. Kjaerulff [74]), rabbit anti-serotonin (1∶1000) (s5545, Sigma), mouse anti-serotonin (1∶150) (Clone 5HT-H209; Dako, Copenhagen, Denmark), rabbit anti-horseradish peroxidase (1∶500) (anti-HRP, # 323-005-021, Jackson ImmunoResearch), rabbit anti-Rheb (1∶1000) (Abcam, Cambridge, UK), rabbit anti-phosphorylated AKT (1∶500) (pAKT; D9E) from Cell Signaling Technology (see [21]), rabbit and mouse anti-GFP (1∶1000) (Invitrogen, Carlsbad, CA), mouse anti-Repo (1∶10) (8D12; Developmental Studies Hybridoma Bank, University of Iowa).

For detection of primary antisera the following secondary antisera were used: goat anti-rabbit Alexa 546 antiserum, goat anti-rabbit Alexa 488 antiserum, goat anti-mouse Alexa 488 antiserum (all from Invitrogen), Cy3-tagged goat anti-rat antiserum and Cy3-tagged goat anti-guinea pig antiserum (Jackson ImmunoResearch, West Grove, PA) were all used at a dilution of 1∶1000.

Rhodamine-phalloidin (Invitrogen) was used at a dilution of 1∶500 to stain muscle. For insulin binding to tissue 1 mg Insulin-FITC (Sigma) was dissolved into 1 ml milli-Q water and used at a dilution of 1∶1000 in 0.01 M PBS. DNA was visualized with 4′,6-Diamidine-2-phenylindole (DAPI; Sigma) at 1∶2000.

### Experiments on effect of diet

For nutrient restriction, eggs of different genotypes collected for 12 h were placed on a diet of 20% sucrose and 5% dry yeast in 2% aqueous agar and second instar larvae (48 h old) were transferred into medium containing 20% sucrose only (see Fig. S15A). The larvae were dissected at the wandering third instar stage for immunocytochemistry. For protein-rich conditions, eggs of different genotypes collected for 12 h were reared on food containing 20% sucrose and 20% yeast, and wandering third instar larvae were collected as above. As a control, flies were reared on food containing 20% sucrose and 5% dry yeast.

### Image analysis

Specimens were imaged with Zeiss LSM 510 META and Zeiss LSM 780 confocal microscopes (Jena, Germany) using 20×, 40× oil or 63× oil immersion objectives. Confocal images were processed with Zeiss LSM software for either projection of z-stacks or single optical sections. Images were edited for contrast and brightness in Adobe Photoshop CS3 Extended version 10.0.

For quantification of immunofluorescence, immunostainings of tissues from different genotypes were carried out in glass vials with same amount of antibodies and all other conditions constant. Confocal images of neurons from different genotypes were obtained with identical laser intensity and scan settings. Immunofluorescence intensity in both cell bodies and tissue background was quantified in a set of regions of interest using Image J 1.40 from NIH, Bethesda, Maryland, USA (http://rsb.info.nih.gov/ij/). Mean fluorescence of projections of each cell body and image background were measured and the final immunofluorescence intensity in cell bodies was determined by subtracting the intensity of the tissue background. Total fluorescence of cell bodies (or nuclei) was calculated as mean fluorescence multiplied with cell size (or nuclear size) in some cases. These are specified in the figure legends of Fig. 9G, Fig. S1D, Fig. S4D, and Fig. S14E. For each genotype neurons in 5–15 brains were measured. The data were analyzed in Prism GraphPad 6.0, with Student's t-test. All data are presented as mean values ± SEM.

For CNS and cell size determination, the outline of CNS or cell body was extracted manually using Image J and its area determined. For each genotype CNS or neurons of 5–15 male flies from 3 independent crosses were measured. For cell size quantification in starved and re-fed flies, male w^1118^ flies were exposed to dry starvation for 18 h and then re-fed with 0.5% aqueous agarose gel for 2 h. For each treatment 7–10 flies were measured. For determination of axon and arborization size in larval body wall muscle, staining of a region of interest (Muscle 8 for anti-LK staining, and Muscle 12–13 for anti-HRP staining) in the 3^rd^ segment was imaged and then quantified by outlining of each axon using Image J. For each genotype neurons in at least 6 body walls were measured.

Images of male 3 d old male flies were obtained with a Leica EZ4HD light microscope (Wetzlar, Germany) and sizes of whole body, wing and abdomen were outlined using Image J and hence quantified. 19–20 flies of each genotype from 3 crosses are measured.

### Functional assays for ABLK neuron manipulations

The capillary feeding (CAFE) assay was conducted according to Ja and others [105] with slight changes. Male flies were placed into 1.5 ml Eppendorf tubes with an inserted 5 µl capillary tube with 5% sucrose, 2% yeast extract and 0.1% propionic acid. Three control food-filled capillaries were inserted in identical tubes without flies. The final consumption of food was determined as the diminished food level minus the average diminishment in control capillaries (due to evaporation). Daily consumption was measured every 24 h and calculated cumulatively over 4 consecutive days. These experiments were run in three replicates with 10 flies of each genotype for each replicate.

For desiccation (dry starvation) experiments, male flies were kept in an incubator at 25°C with 12∶12 h light∶dark (LD) conditions and controlled humidity. Flies aged 4–6 d were exposed to desiccation in bottles with neither food nor water. The number of dead flies was monitored every 12 h. These experiments were run in three replicates with at least 30 flies of each genotype per replicate.

## Supporting Information

Figure S1 Manipulations of dInR levels affect InR-immunolabeling. **A–D** We used an antiserum to the phosphorylated human insulin receptor β (Tyr1146; code #3021) to determine InR immunolevels in larval ABLKs after dInR-RNAi and over expression. The dInR-RNAi produces a loss of immunolabeling in the posterior ABLKs (A3–7) and a reduction in the anterior ones. Total InR immunofluorescence decreases significantly (see D). Over expression of a constitutively active dInR (dInR-CA) increases the cell body size of the ABLKs as well as the total immunofluorescence. **D** Quantification of total InR immunofluorescence (mean fluorescence multiplied by cell size; **p<0.01, ***p<0.001, n = 5–8 animals for each genotype from 3 crosses; unpaired Student's T-test). **E**. Adult distribution of InR immunolabeling in ABLKs (anterior adult-specific and posterior ones), using another antiserum to phosphorylated human insulin receptor β (Tyr1150/1151; code #3024). See also Fig. S2 H for adult InR immunolabeling combined with *Lk*-Gal4-GFP.
(TIF)

Figure S2 Expression of InR immunoreactivity in CNS and fat body. **A–D** In the larval fat body the cell surfaces display InR immunolabeling as well as binding of FITC-tagged bovine insulin (**A**). **B–D** shows details of the immunolabeling and insulin-FITC binding. In panel **D** only insulin-FITC was applied without anti-InR. **E–G** InR immunolabeling in the adult brain identifies the LHLK neurons (not shown here) and some additional neurons dorsally in the brain (**E**). InR immunolabeling in neuropil of pars intercerebralis (**F**) is derived from neurons in the tritocerebrum (**G**). **H1–H2** Antiserum to Mosquito InR (AedInR) labels the ABLKs in the adult abdominal ganglia seen in Lk-Gal4-GFP expressing CNS. **I** Photoreceptor axons in the developing imaginal optic lobe label with InR antiserum.
(TIF)

Figure S3 Pan-neuronal and c929-Gal4 driven dInR manipulations alter ABLK neuron cell body sizes. **A–E** Using *Elav*-Gal4-driven dInR manipulations only RNAi affected cell body size; a significant size reduction was seen (n = 6 flies from 3 crosses for each genotype; unpaired Student's T-test). Details are shown in A1–D1. **F–I** The c929-Gal4 driver (representing *Dimm* expression) induces both reduction (dInR-RNAi) and increase (dInR over expression) of ABLK neuron cell bodies (**p<0.01, ***p<0.001, n = 8–10 animals for each genotype from 3 crosses; unpaired Student's T-test).
(TIF)

Figure S4 Phosphorylated Akt (pAkt) is expressed in ABLK neurons of control animals and influenced by dInR expression levels. **A** Antiserum to pAkt labels a subpopulation of the c929 (*Dimm*) expressing neurons in the abdominal ganglia (some at arrows), as well as in c929-negative neurons. **B** All the ABLKs express pAkt in the third instar larva. In A and B the pAKT expression represents control levels since no dInR manipulations were made. **C** Over expression of dInR with the c929-Gal4 increases cell size of ABLKs (arrows in **C3**) and pAkt immunofluorescence (**C3, D**), whereas dInR-RNAi has no significant effect (**C1, D**) (*p<0.05, n = 7–10 animals for each genotype from 3 crosses; unpaired Student's T-test). **E–G** Further distribution of pAkt immunolabeling in non-treated wild type flies. **E** Also the larval LHLK neurons (arrows), but not the LK-negative ALK neurons, express pAkt label. **F** The larval mushroom body Kenyon cells with their cell bodies above the calyx (Ca) and axons in the peduncle (P) and alpha (aL) and beta (bL) lobes express pAkt label (and some other non-identified neurons). G Overview of pAkt immunolabeled neurons in the brain subesophageal ganglion (SEG) and thoracic ganglion (ThG).
(TIF)

Figure S5 Over expression of Rheb in adult ABLK neurons affects cell body size. **A–D** Over expression of Rheb in LK neurons increases size of both anterior and posterior neurons, whereas knockdown of Rheb diminishes both (**p<0.01, ***p<0.001, n = 5–9 animals for each genotype from 3 crosses, unpaired Student's T-test). Note also that Rheb over expression produces cell bodies with irregular outlines.
(TIF)

Figure S6 Manipulation of TOR and S6K affect ABLK cell body size in larval CNS. **A** ABLKs in control CNS. **B** Over expression of a dominant negative form of TOR (dTor-TED) diminishes cell bodies of ABLKs. **C–E** A dominant negative form of S6K (S6K-KQ) reduces cell body size, whereas expression of wild type S6K and a constitutively active form (dS6K-CA) increases size. **F** Quantification of ABLK cell body size changes (**p<0.01, ***p<0.001, n = 6–12 animals for each genotype from 3 crosses; unpaired Student's T-test).
(TIF)

Figure S7 Manipulations of ALK levels in LK neurons have no effect on cell body size in normally fed larvae. **A–E** Expressing a dominant negative ALK (ALK-DN), wild type ALK or its constitutively active form (ALK-CA) with Lk-Gal4 does not affect ABLK cell body size (ns not significant, n = 8–13 animals for each genotype from 3 crosses; unpaired Student's T-test).
(TIF)

Figure S8 Manipulation of dInR levels in motor neurons does not affect peripheral axon morphology. **A–D** Using the OK6-Gal4 driver we knocked down or over expressed the dInR (and its active form dInR-CA). Axons were labeled with anti-HRP and muscles with phalloidin-rhodamine. No overt changes in axon diameters, bouton sizes of axon branching were discovered (not quantified here). The muscle 8 (M8) supplied by LK axons (and other axons) is indicated. In **A** the box indicates the area of M12 and 13 analyzed in Fig. 5.
(TIF)

Figure S9 The cell body size of insulin producing cells of adult flies is affected by dInR and PI3K manipulations. The insulin producing cells were labeled with anti-DILP2. Both knockdown and over expression of the dInR affects cell body size (**A–D** and **G**) (*p<0.05, ***p<0.001, n = 7–10 animals for each genotype from 3 crosses, unpaired Student's T-test). **E** and **F** Over expression of PI3K increases cell body size (**F, H**) and the dominant negative PI3K (PI3K-DN) diminishes it (**E, H**) (*p<0.05, **p<0.01, n = 5–7 animals for each genotype from 3 crosses; unpaired Student's T-test).
(TIF)

Figure S10 Further types of Dimm-positive neurons also respond to changes of dInR levels. **A–D** The size of the cell bodies of the DILP7 producing neurons of the abdominal ganglia were altered by dInR manipulations using a *Dilp7*-Gal4. Cells are shown from segments A6–9 (**A–C**) and the DP neurons in the first segment (**A1–C1**). Neurons were visualized with antiserum to DILP7. Quantification summarized in **D** (*p<0.05, **p<0.01, ***p<0.001, n = 10–18 animals for each genotype from 3 crosses; unpaired Student's T-test). **E–G** Two types of Dimm positive neurons that produce the neuropeptide pigment-dispersing factor (PDF) were tested. **E** and **G** Cell bodies of the six to eight PDF neurons posteriorly in abdominal ganglia of larvae (Abd in **G**) displayed increased size after dInR over expression, but RNAi had no effect (**p<0.01, ***p<0.001, n = 8–9 animals for each genotype from 3 crosses; unpaired Student's T-test). **F** and **G** The cell bodies of the adult clock neurons designated large LN_v_s also responded by drastic size increase to dInR over expression (n = 6–9 flies for each genotype from 3 crosses; unpaired Student's T-test). Me, medulla of optic lobe.
(TIF)

Figure S11 Effects of DILP2 and DILP7 on neuron growth. **A** The abdominal neurons producing DILP7 (**A1**) have arborizations superimposing those of the LK neurons (**A2**). Thus we tested manipulations of these and the insulin producing cells (IPCs) of the brain for effects on LK neuron size. **B–F** We deleted (using UAS reaper, Rpr) or hyperpolarized (UAS-Ork) the IPCs (using *Dilp2*-Gal4) and hyperpolarized Dilp7-Gal4 expressing neurons and monitored ABLK neuron cell bodies. Only Dilp2-Rpr (**B**–**D**) and Dilp2-Ork (**F**) lead to significantly decreased cell body size of LK neurons (**p<0.01, ***p<0.001, n = 6–10 animals for each genotype from 3 crosses; unpaired Student's T-test). Dilp7-Ork reduced LK-immunofluorescence (**E**), but not cell size. Also Dilp2>Ork and Rpr reduced immunofluorescence (**E**). Depolarizing IPCs with Dilp2>NaChBac (F) did not increase LK neuron size. **G** and **H** The decrease of LK neuron size after IPC manipulations is likely to be a result of over-all diminishment of the CNS volume. The CNS of Dilp2>Ork larvae was reduced in size, whereas the CNS Dilp2>NaChBac larvae resembled that of controls (**p<0.01, ***p<0.001, n = 7–12 brains for each genotype from 3 crosses; unpaired Student's T-test). **I** and **J** Also the size of the body, the abdomen and wings of adult fly was smaller after hyperpolarization of the IPCs with Dilp2>Ork (***p<0.001, n = 19–20 animals for each genotype from 3 crosses; unpaired Student's T-test).
(TIF)

Figure S12 Expression of Dilp6 in CNS of first instar larvae. **A** Dilp6-Gal4 driven mcd8-GFP (green) in relation to LK immunolabeled ABLK neurons (magenta). Many cells express Dilp6 in abdominal ganglia. **B** Higher magnification of ventral nerve cord. Note Dilp6 expression in surface glia cells (arrow). **C** Using a nuclear GFP reporter we show that Dilp6-Gal4 expression is mainly in glial cells as determined by antiserum to Repo (magenta). The colocalization of the two nuclear marks is seen as whitish labeling. Nuclei of surface glial cells can be seen (e. g. at arrows).
(TIF)

Figure S13 Over expression of the dInR in DIMM neurons increases PICK1 expression. **A–C** We used an antiserum to PICK1 to monitor the approximate abundance of trans-Golgi network units. The association of PICK1 immunolabeling with Golgi was determined in *Drosophila* brain neurons with a Golgi marker GRASP (see [74] and Fig. 10 **F**). Over expression of dInR affected the PICK1 immunolabeled cell size as well as total relative PICK1 fluorescence in c929-Gal4 expressing neurons of the larval abdominal ganglia, quantified in **D** and **E** (*p<0.05, **p<0.01, n = 5 animals for each genotype from 3 crosses; unpaired Student's T-test). The increased PICK1 immunofluorescence indicates addition of trans-Golgi units or increased Golgi volume. No effect on PICK1 immunolabeling was seen after dInR-RNAi. **F** PICK1 immunolabeling in cell body of ABLK neuron is associated with UAS-*Grasp*-GFP expression.
(TIF)

Figure S14 Over expression of dInR in IPCs leads to increased nuclear size. **A** After manipulations of dInR in IPCs (Using *Dilp2*-Gal4) the nuclear size in IPCs (labeled with anti-DILP2; red) was determined by DAPI staining (blue). **B** and **C** The nuclei of the IPCs indicated by arrows in **A** are shown after DAPI staining (IPC nuclei indicated by arrows in C). D Quantification of nuclear size in IPCs after dInR manipulations. Overexpression of the dInR induced larger nuclei (***p<0.001, Unpaired Students' T-test; n = 6–8 animals for each genotype from 3 crosses). **E** Quantification of total DAPI fluorescence (mean fluorescence multiplied by cell size) in IPCs after receptor manipulations. No significant change in fluorescence intensity was seen (for the Dilp2>dInR p>0.05, Unpaired Student's T-test; n = 6–8 animals for each genotype from 3 crosses). Note that the images of DAPI staining shown here are saturated to clearly visualize the nuclei; for intensity measurements, they were not saturated.
(TIF)

Figure S15 Protein levels in diet affect size of ABLKs. **A** The diets and feeding regimes used in experiments. Standard food is based on the one used by Bloomington *Drosophila* Stock Center. This is used in most experiments in this paper. Three experimental diets were tested for experiments in Fig. 11 and here in panels B–D. We combined 20% sucrose with 0, 5 and 20% yeast paste; two of these were based on sucrose and yeast only, and one a combination of 20% sucrose and 5% yeast until the proposed critical size of larvae was reached (at 60 h) and then 20% sucrose only. Wandering third instar larvae were used for imaging (about 108 h development). **B** Details of ABLK cell bodies (anti-LK; magenta) and 5-HT immunoreactive ones (green) in third instar larvae where dInR was manipulated in Lk-Gal4 neurons (and different protein diets). The whole ganglia are shown in Fig. 11A and B. **C** Cell bodies of ABLKs of wild type (w1118) larvae feed three different diets. **D** Quantification of ABLK cell body sizes in the diet experiment shown in C. Cell size increases significantly with protein levels (*p<0.05, **p<0.01, ***p<0.001, n = 6–13 larvae for each genotype from 3 crosses; unpaired Student's T-test).
(TIF)

Figure S16 A scheme of the nutrient sensing TOR and insulin signaling pathways. This scheme is a compilation of several published ones (see [17], [47]) and display key features of interest in the present paper. The two pathways intersect at the level of Akt and TSC1/2 and converge on regulation of protein synthesis and growth (via ribosome biogenesis and translation apparatus). The Alk signaling pathway is activated only at nutritional restriction (NR) and inhibits the two other pathways to ensure CNS growth as a super sparing of this tissue at the cost of other tissues. However at low nutrients conditions the Alk signaling activates the pathway downstream the dInR (PI3K). Arrows depict activating signals and the T-shaped connectors inhibitory ones.
(TIF)

Table S1 Manipulations of dInR expression in different cell types: numerical data. This table provides numerical data for manipulations of genes in Dimm positive and Dimm negative peptidergic neuroendocrine cells. In this table only the PTTH neurons are DIMM negative. Values are given as means ± SEM. n = number of animals tested; *p<0.05, **p<0.01, ***p<0.001, ns not significant (Unpaired Student's T-test), data are presented as mean values ± SEM. L1 = 1st instar larva, L3 = 3rd instar larva, A 3 d = 3 d old adult flies, A 35 d = 35 d old adult flies, ant = anterior LK neurons, post = posterior LK neurons, R-neur = R-neurons of ellipsoid body.
(DOCX)

Table S2 Manipulations of PI3K and Rheb expression in different cell types: numerical data. This table provides numerical data for manipulations of genes in Dimm positive peptidergic neuroendocrine cells. Values are given as means ± SEM. n = number of animals tested; *p<0.05, **p<0.01, ***p<0.001, ns not significant (Unpaired Student's T-test), data are presented as mean values ± SEM. L3 = 3rd instar larva, A 3 d = 3 d old adult flies, A 35 d = 35 d old adult flies, ant = anterior LK neurons, post = posterior LK neurons, R-neur = R-neurons of ellipsoid body.
(DOCX)
