# Supplementary figures and images for: Insulin/IGF-Regulated Size Scaling of Neuroendocrine Cells Expressing the bHLH Transcription Factor Dimmed in Drosophila
Source: PLoS Genet. 2013 Dec 26;9(12):e1004052. doi: 10.1371/journal.pgen.1004052 (PMC3873260; doi:10.1371/journal.pgen.1004052)

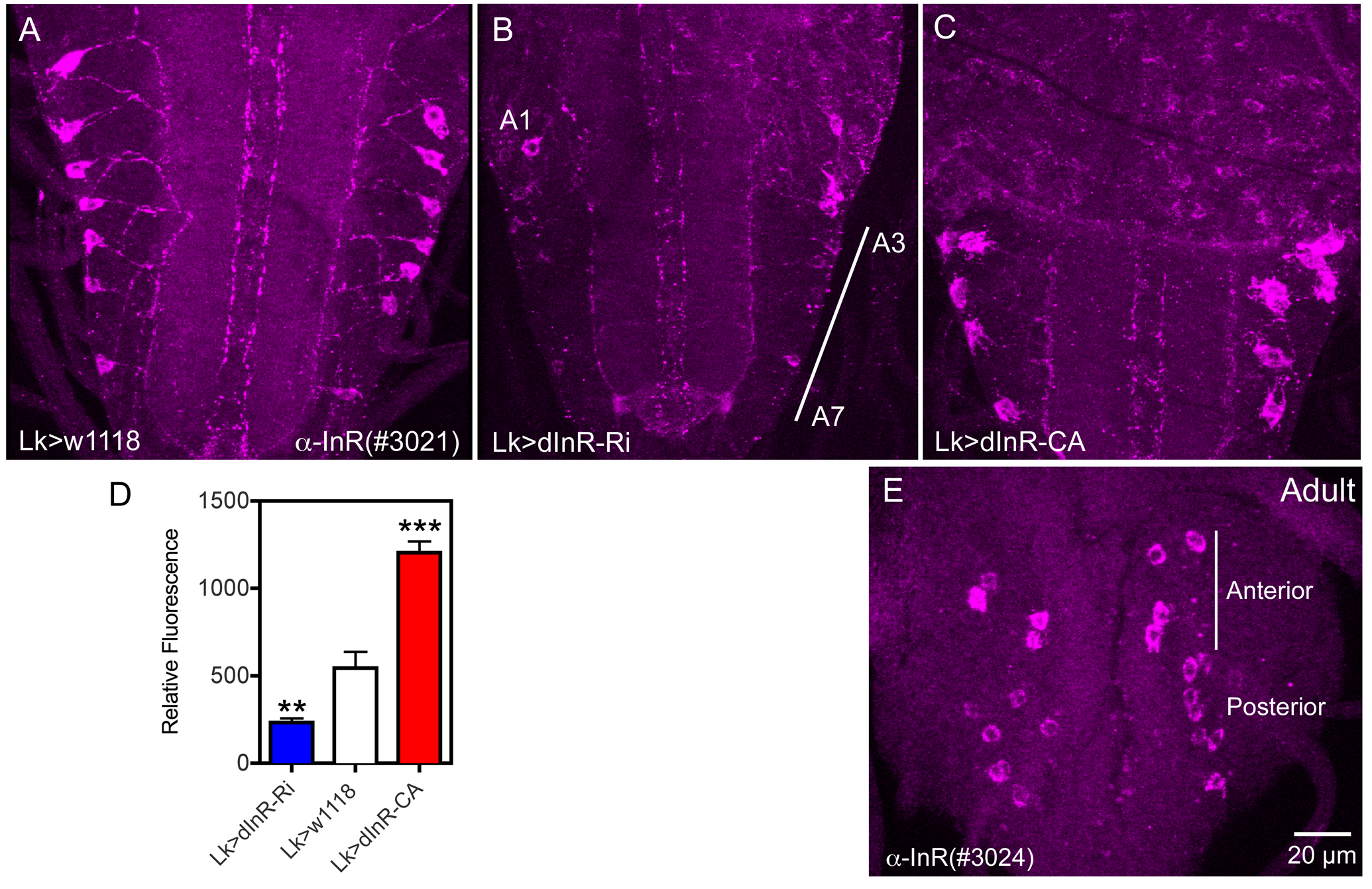

Supplement: Figure S1 — Manipulations of dInR levels affect InR-immunolabeling. A–D We used an antiserum to the phosphorylated human insulin receptor β (Tyr1146; code #3021) to determine InR immunolevels in larval ABLKs after dInR-RNAi and over expression. The dInR-RNAi produces a loss of immunolabeling in the posterior ABLKs (A3–7) and a reduction in the anterior ones. Total InR immunofluorescence decreases significantly (see D). Over expression of a constitutively active dInR (dInR-CA) increases the cell body size of the ABLKs as well as the total immunofluorescence. D Quantification of total InR immunofluorescence (mean fluorescence multiplied by cell size; **p<0.01, ***p<0.001, n = 5–8 animals for each genotype from 3 crosses; unpaired Student's T-test). E. Adult distribution of InR immunolabeling in ABLKs (anterior adult-specific and posterior ones), using another antiserum to phosphorylated human insulin receptor β (Tyr1150/1151; code #3024). See also Fig. S2 H for adult InR immunolabeling combined with Lk-Gal4-GFP. (TIF) [file pgen.1004052.s001.tif]

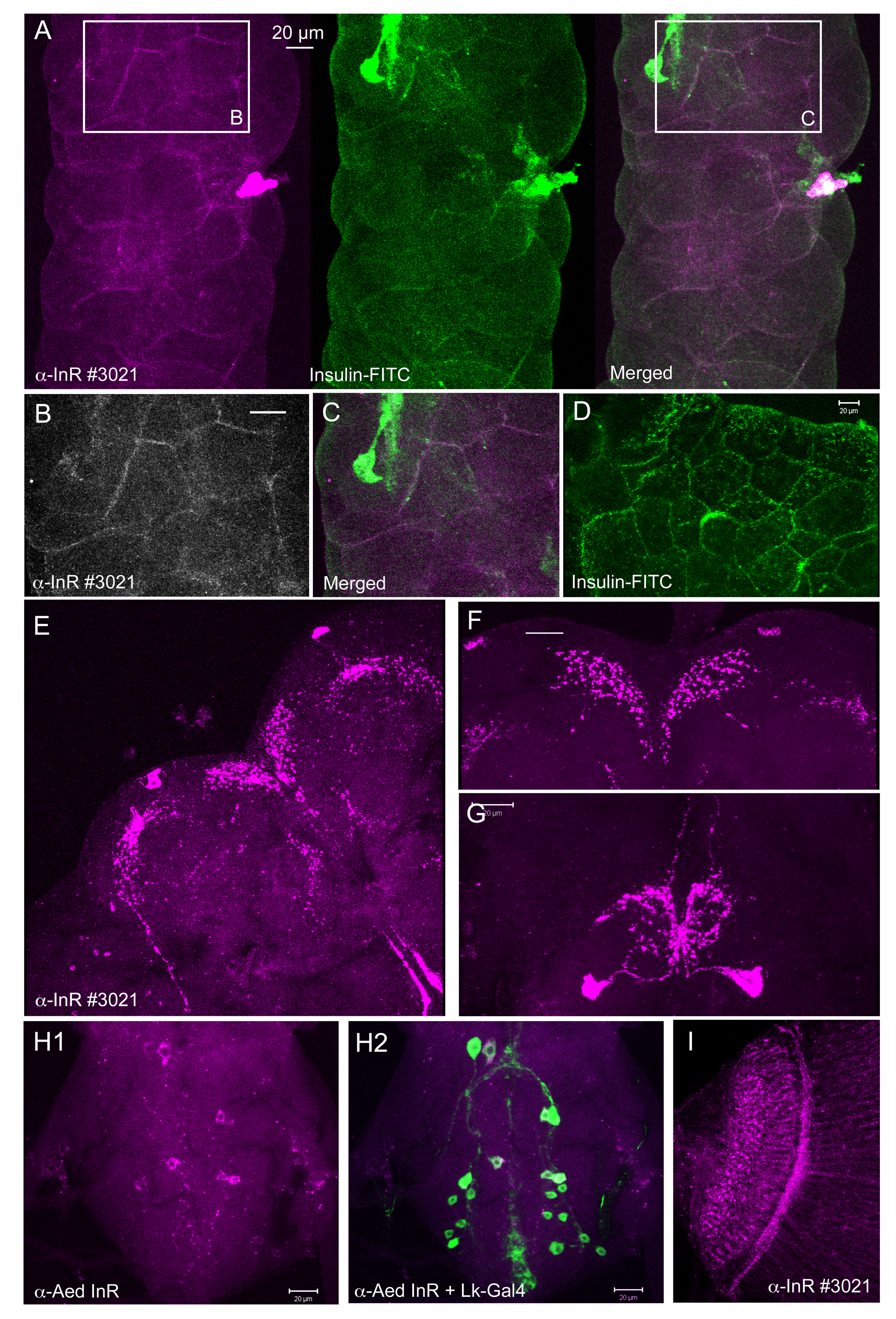

Supplement: Figure S2 — Expression of InR immunoreactivity in CNS and fat body. A–D In the larval fat body the cell surfaces display InR immunolabeling as well as binding of FITC-tagged bovine insulin (A). B–D shows details of the immunolabeling and insulin-FITC binding. In panel D only insulin-FITC was applied without anti-InR. E–G InR immunolabeling in the adult brain identifies the LHLK neurons (not shown here) and some additional neurons dorsally in the brain (E). InR immunolabeling in neuropil of pars intercerebralis (F) is derived from neurons in the tritocerebrum (G). H1–H2 Antiserum to Mosquito InR (AedInR) labels the ABLKs in the adult abdominal ganglia seen in Lk-Gal4-GFP expressing CNS. I Photoreceptor axons in the developing imaginal optic lobe label with InR antiserum. (TIF) [file pgen.1004052.s002.tif]

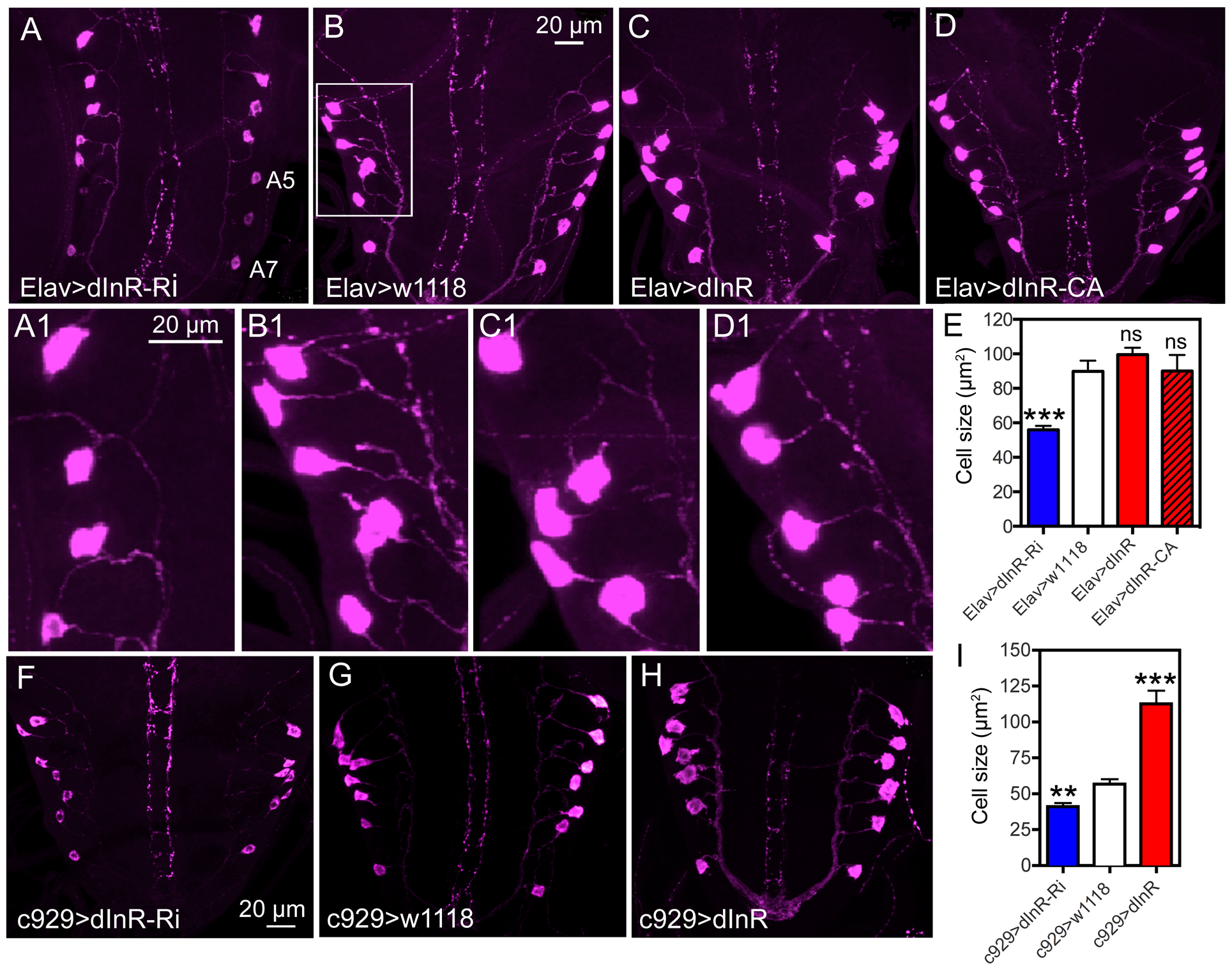

Supplement: Figure S3 — Pan-neuronal and c929-Gal4 driven dInR manipulations alter ABLK neuron cell body sizes. A–E Using Elav-Gal4-driven dInR manipulations only RNAi affected cell body size; a significant size reduction was seen (n = 6 flies from 3 crosses for each genotype; unpaired Student's T-test). Details are shown in A1–D1. F–I The c929-Gal4 driver (representing Dimm expression) induces both reduction (dInR-RNAi) and increase (dInR over expression) of ABLK neuron cell bodies (**p<0.01, ***p<0.001, n = 8–10 animals for each genotype from 3 crosses; unpaired Student's T-test). (TIF) [file pgen.1004052.s003.tif]

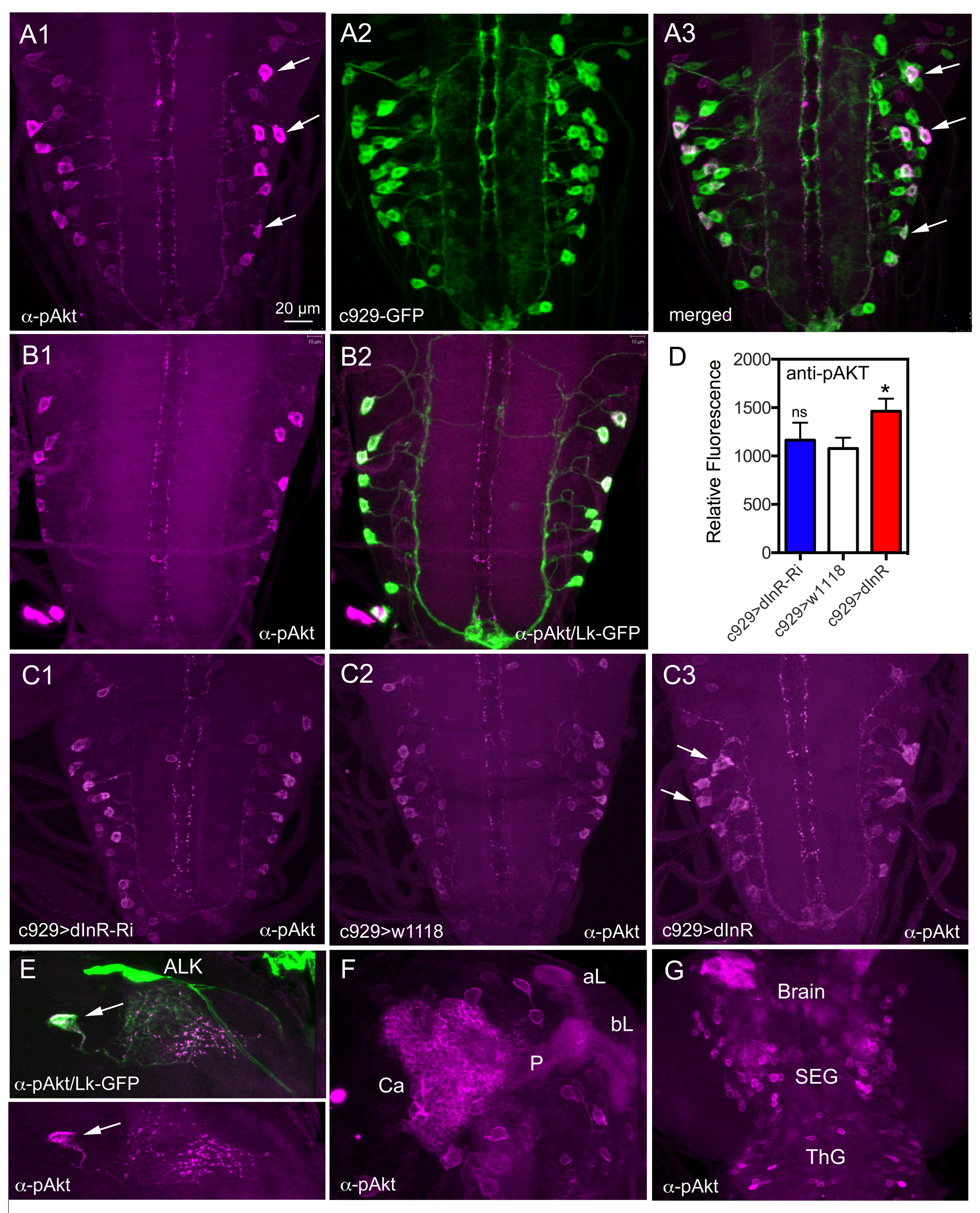

Supplement: Figure S4 — Phosphorylated Akt (pAkt) is expressed in ABLK neurons of control animals and influenced by dInR expression levels. A Antiserum to pAkt labels a subpopulation of the c929 (Dimm) expressing neurons in the abdominal ganglia (some at arrows), as well as in c929-negative neurons. B All the ABLKs express pAkt in the third instar larva. In A and B the pAKT expression represents control levels since no dInR manipulations were made. C Over expression of dInR with the c929-Gal4 increases cell size of ABLKs (arrows in C3) and pAkt immunofluorescence (C3, D), whereas dInR-RNAi has no significant effect (C1, D) (*p<0.05, n = 7–10 animals for each genotype from 3 crosses; unpaired Student's T-test). E–G Further distribution of pAkt immunolabeling in non-treated wild type flies. E Also the larval LHLK neurons (arrows), but not the LK-negative ALK neurons, express pAkt label. F The larval mushroom body Kenyon cells with their cell bodies above the calyx (Ca) and axons in the peduncle (P) and alpha (aL) and beta (bL) lobes express pAkt label (and some other non-identified neurons). G Overview of pAkt immunolabeled neurons in the brain subesophageal ganglion (SEG) and thoracic ganglion (ThG). (TIF) [file pgen.1004052.s004.tif]

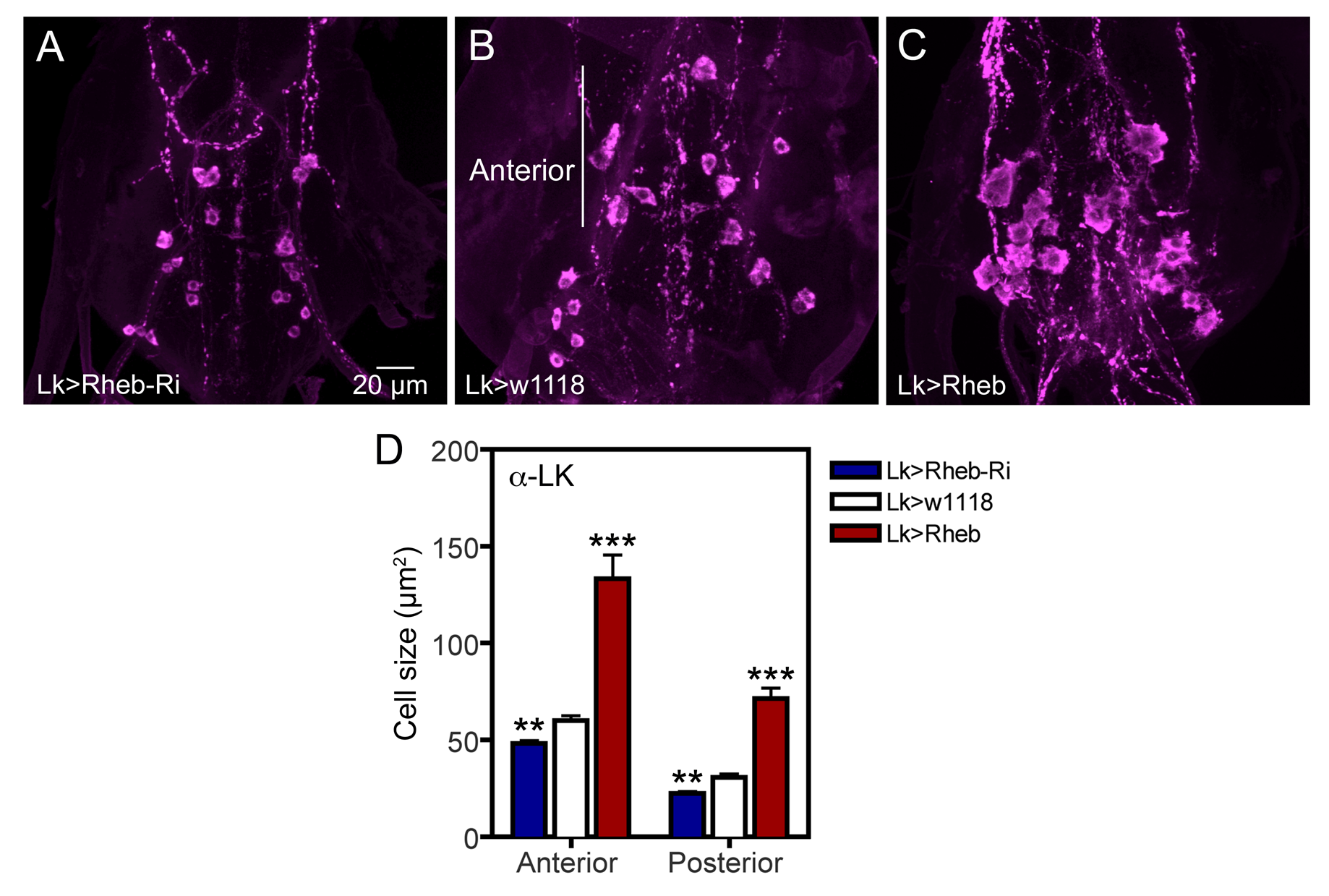

Supplement: Figure S5 — Over expression of Rheb in adult ABLK neurons affects cell body size. A–D Over expression of Rheb in LK neurons increases size of both anterior and posterior neurons, whereas knockdown of Rheb diminishes both (**p<0.01, ***p<0.001, n = 5–9 animals for each genotype from 3 crosses, unpaired Student's T-test). Note also that Rheb over expression produces cell bodies with irregular outlines. (TIF) [file pgen.1004052.s005.tif]

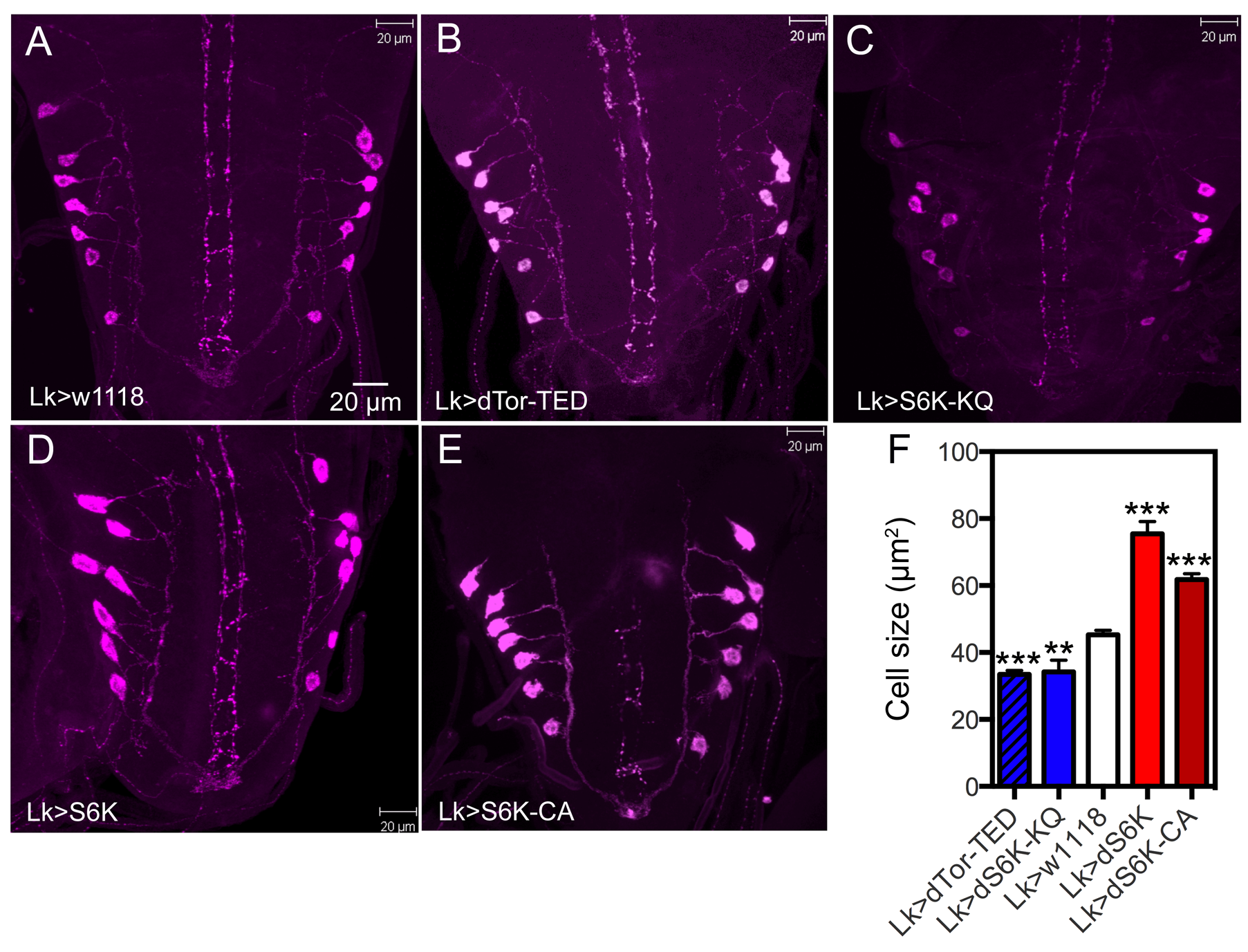

Supplement: Figure S6 — Manipulation of TOR and S6K affect ABLK cell body size in larval CNS. A ABLKs in control CNS. B Over expression of a dominant negative form of TOR (dTor-TED) diminishes cell bodies of ABLKs. C–E A dominant negative form of S6K (S6K-KQ) reduces cell body size, whereas expression of wild type S6K and a constitutively active form (dS6K-CA) increases size. F Quantification of ABLK cell body size changes (**p<0.01, ***p<0.001, n = 6–12 animals for each genotype from 3 crosses; unpaired Student's T-test). (TIF) [file pgen.1004052.s006.tif]

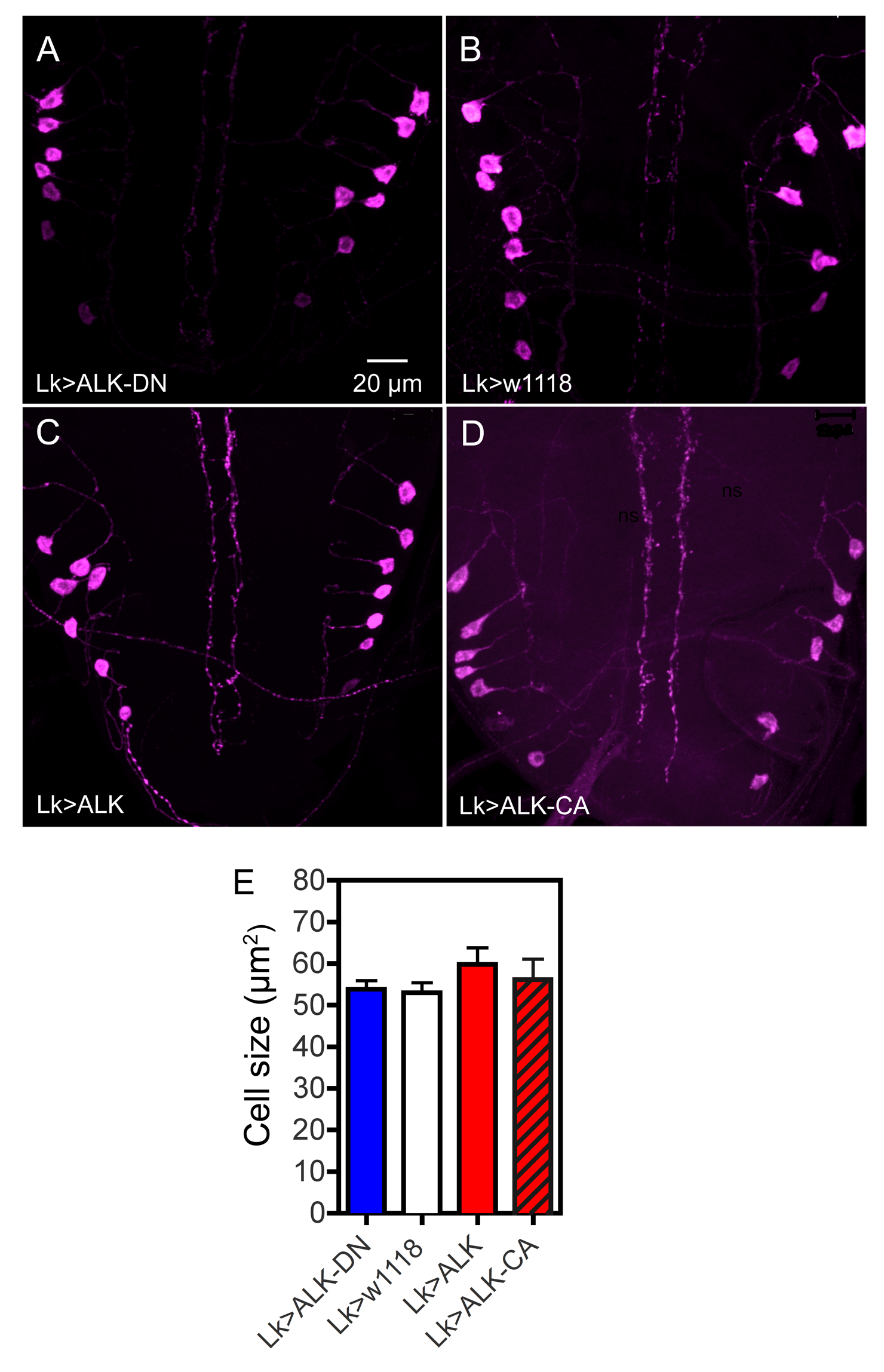

Supplement: Figure S7 — Manipulations of ALK levels in LK neurons have no effect on cell body size in normally fed larvae. A–E Expressing a dominant negative ALK (ALK-DN), wild type ALK or its constitutively active form (ALK-CA) with Lk-Gal4 does not affect ABLK cell body size (ns not significant, n = 8–13 animals for each genotype from 3 crosses; unpaired Student's T-test). (TIF) [file pgen.1004052.s007.tif]

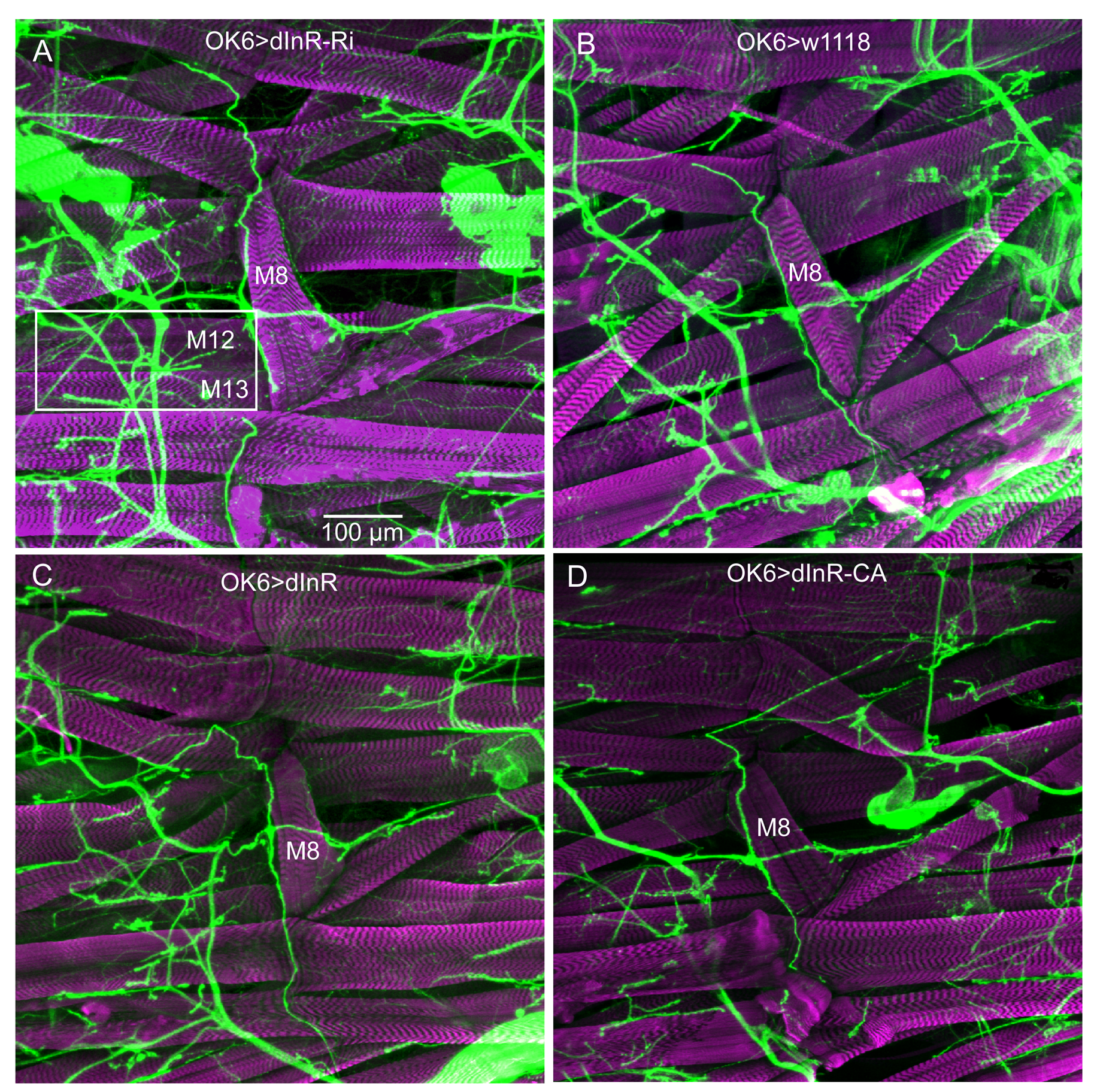

Supplement: Figure S8 — Manipulation of dInR levels in motor neurons does not affect peripheral axon morphology. A–D Using the OK6-Gal4 driver we knocked down or over expressed the dInR (and its active form dInR-CA). Axons were labeled with anti-HRP and muscles with phalloidin-rhodamine. No overt changes in axon diameters, bouton sizes of axon branching were discovered (not quantified here). The muscle 8 (M8) supplied by LK axons (and other axons) is indicated. In A the box indicates the area of M12 and 13 analyzed in Fig. 5. (TIF) [file pgen.1004052.s008.tif]

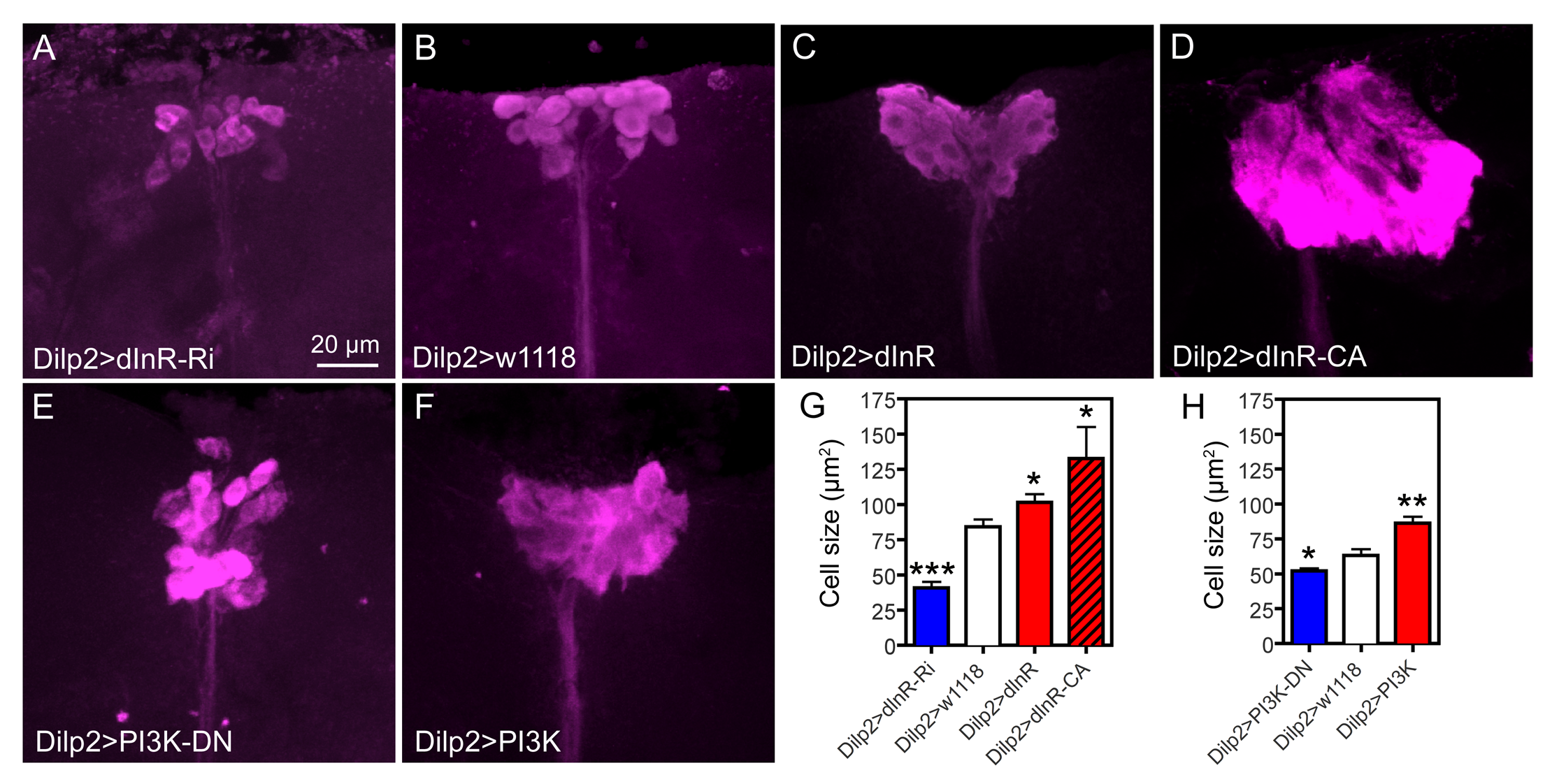

Supplement: Figure S9 — The cell body size of insulin producing cells of adult flies is affected by dInR and PI3K manipulations. The insulin producing cells were labeled with anti-DILP2. Both knockdown and over expression of the dInR affects cell body size (A–D and G) (*p<0.05, ***p<0.001, n = 7–10 animals for each genotype from 3 crosses, unpaired Student's T-test). E and F Over expression of PI3K increases cell body size (F, H) and the dominant negative PI3K (PI3K-DN) diminishes it (E, H) (*p<0.05, **p<0.01, n = 5–7 animals for each genotype from 3 crosses; unpaired Student's T-test). (TIF) [file pgen.1004052.s009.tif]

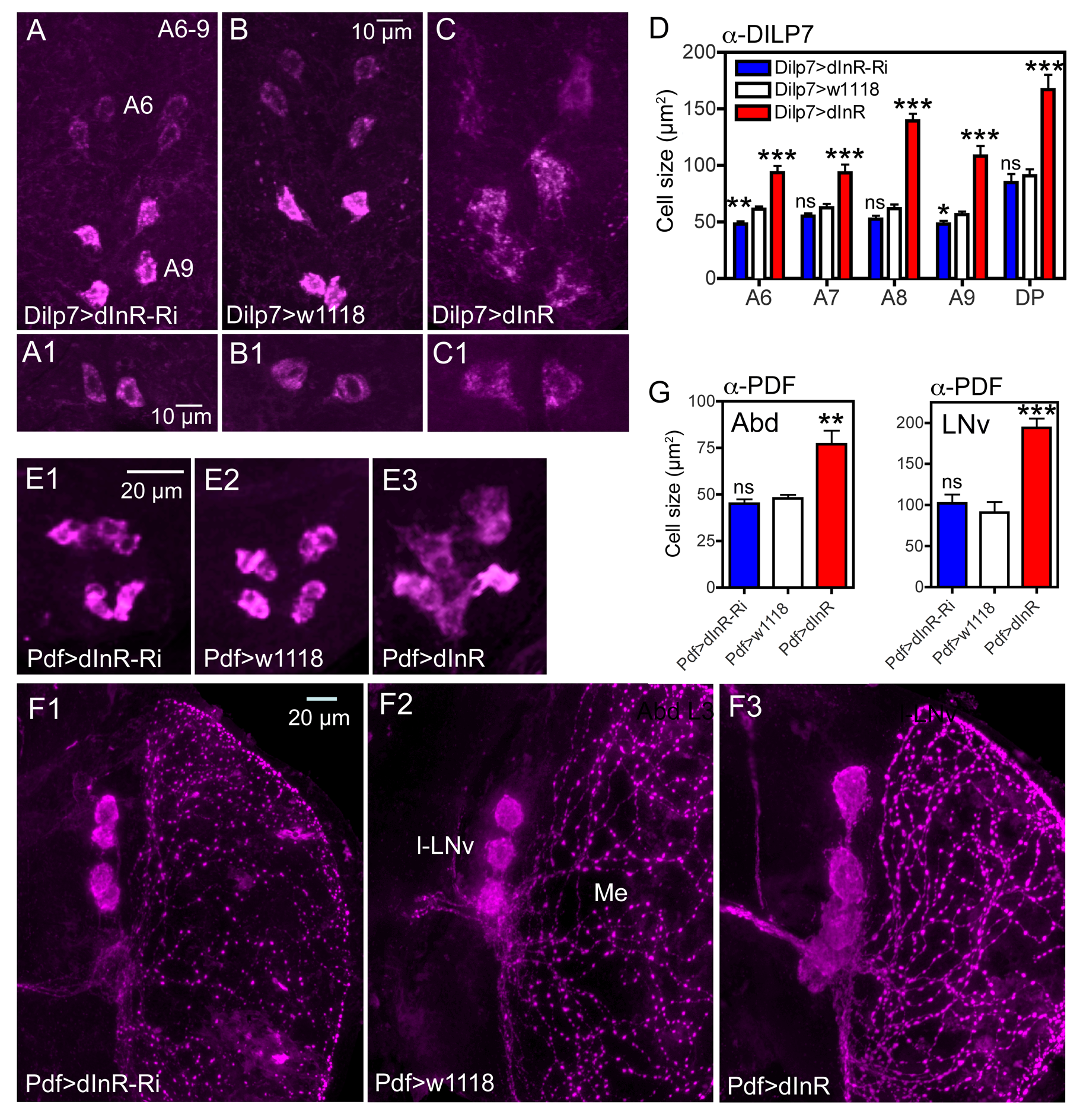

Supplement: Figure S10 — Further types of Dimm-positive neurons also respond to changes of dInR levels. A–D The size of the cell bodies of the DILP7 producing neurons of the abdominal ganglia were altered by dInR manipulations using a Dilp7-Gal4. Cells are shown from segments A6–9 (A–C) and the DP neurons in the first segment (A1–C1). Neurons were visualized with antiserum to DILP7. Quantification summarized in D (*p<0.05, **p<0.01, ***p<0.001, n = 10–18 animals for each genotype from 3 crosses; unpaired Student's T-test). E–G Two types of Dimm positive neurons that produce the neuropeptide pigment-dispersing factor (PDF) were tested. E and G Cell bodies of the six to eight PDF neurons posteriorly in abdominal ganglia of larvae (Abd in G) displayed increased size after dInR over expression, but RNAi had no effect (**p<0.01, ***p<0.001, n = 8–9 animals for each genotype from 3 crosses; unpaired Student's T-test). F and G The cell bodies of the adult clock neurons designated large LNvs also responded by drastic size increase to dInR over expression (n = 6–9 flies for each genotype from 3 crosses; unpaired Student's T-test). Me, medulla of optic lobe. (TIF) [file pgen.1004052.s010.tif]

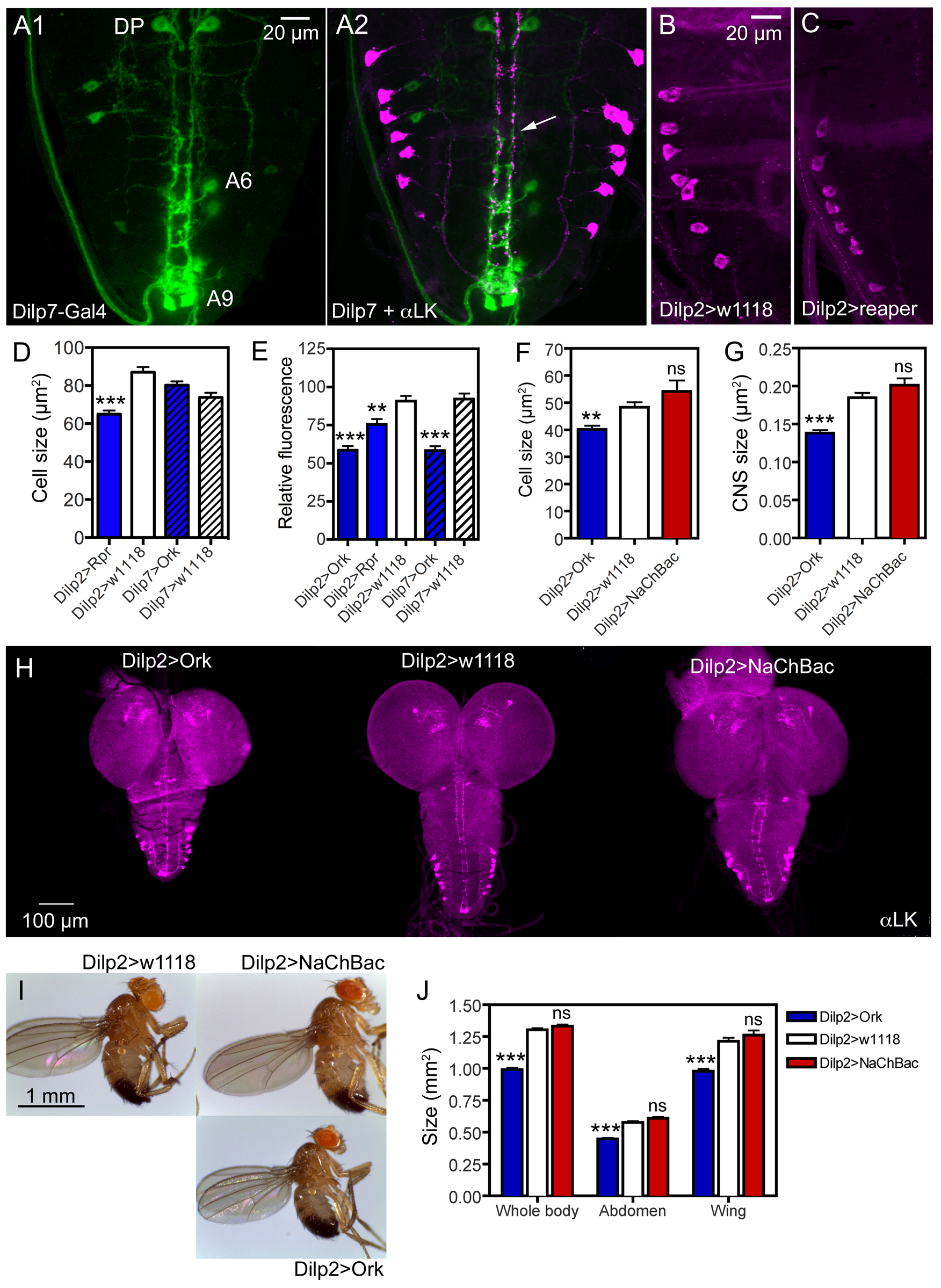

Supplement: Figure S11 — Effects of DILP2 and DILP7 on neuron growth. A The abdominal neurons producing DILP7 (A1) have arborizations superimposing those of the LK neurons (A2). Thus we tested manipulations of these and the insulin producing cells (IPCs) of the brain for effects on LK neuron size. B–F We deleted (using UAS reaper, Rpr) or hyperpolarized (UAS-Ork) the IPCs (using Dilp2-Gal4) and hyperpolarized Dilp7-Gal4 expressing neurons and monitored ABLK neuron cell bodies. Only Dilp2-Rpr (B–D) and Dilp2-Ork (F) lead to significantly decreased cell body size of LK neurons (**p<0.01, ***p<0.001, n = 6–10 animals for each genotype from 3 crosses; unpaired Student's T-test). Dilp7-Ork reduced LK-immunofluorescence (E), but not cell size. Also Dilp2>Ork and Rpr reduced immunofluorescence (E). Depolarizing IPCs with Dilp2>NaChBac (F) did not increase LK neuron size. G and H The decrease of LK neuron size after IPC manipulations is likely to be a result of over-all diminishment of the CNS volume. The CNS of Dilp2>Ork larvae was reduced in size, whereas the CNS Dilp2>NaChBac larvae resembled that of controls (**p<0.01, ***p<0.001, n = 7–12 brains for each genotype from 3 crosses; unpaired Student's T-test). I and J Also the size of the body, the abdomen and wings of adult fly was smaller after hyperpolarization of the IPCs with Dilp2>Ork (***p<0.001, n = 19–20 animals for each genotype from 3 crosses; unpaired Student's T-test). (TIF) [file pgen.1004052.s011.tif]

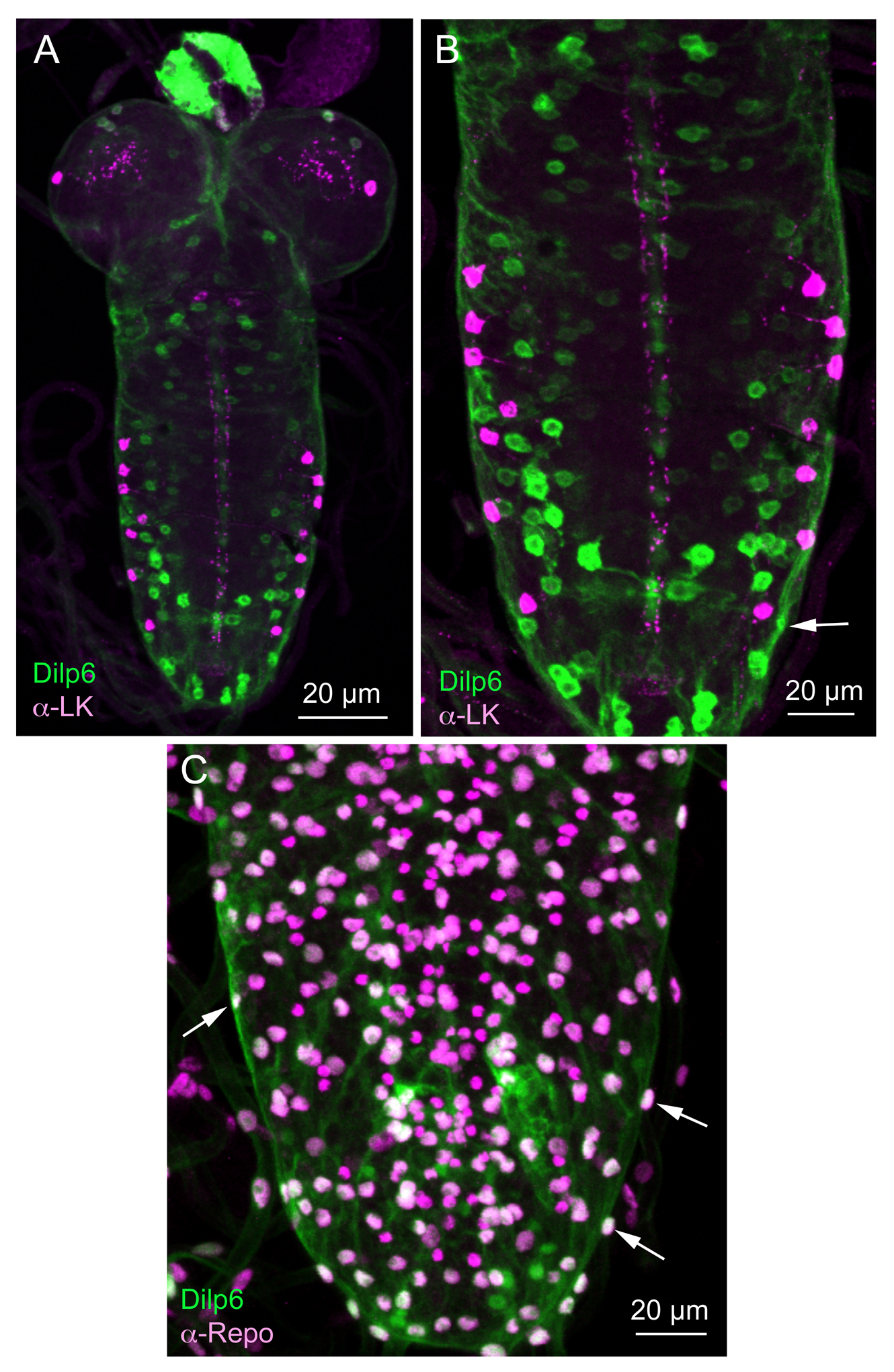

Supplement: Figure S12 — Expression of Dilp6 in CNS of first instar larvae. A Dilp6-Gal4 driven mcd8-GFP (green) in relation to LK immunolabeled ABLK neurons (magenta). Many cells express Dilp6 in abdominal ganglia. B Higher magnification of ventral nerve cord. Note Dilp6 expression in surface glia cells (arrow). C Using a nuclear GFP reporter we show that Dilp6-Gal4 expression is mainly in glial cells as determined by antiserum to Repo (magenta). The colocalization of the two nuclear marks is seen as whitish labeling. Nuclei of surface glial cells can be seen (e. g. at arrows). (TIF) [file pgen.1004052.s012.tif]

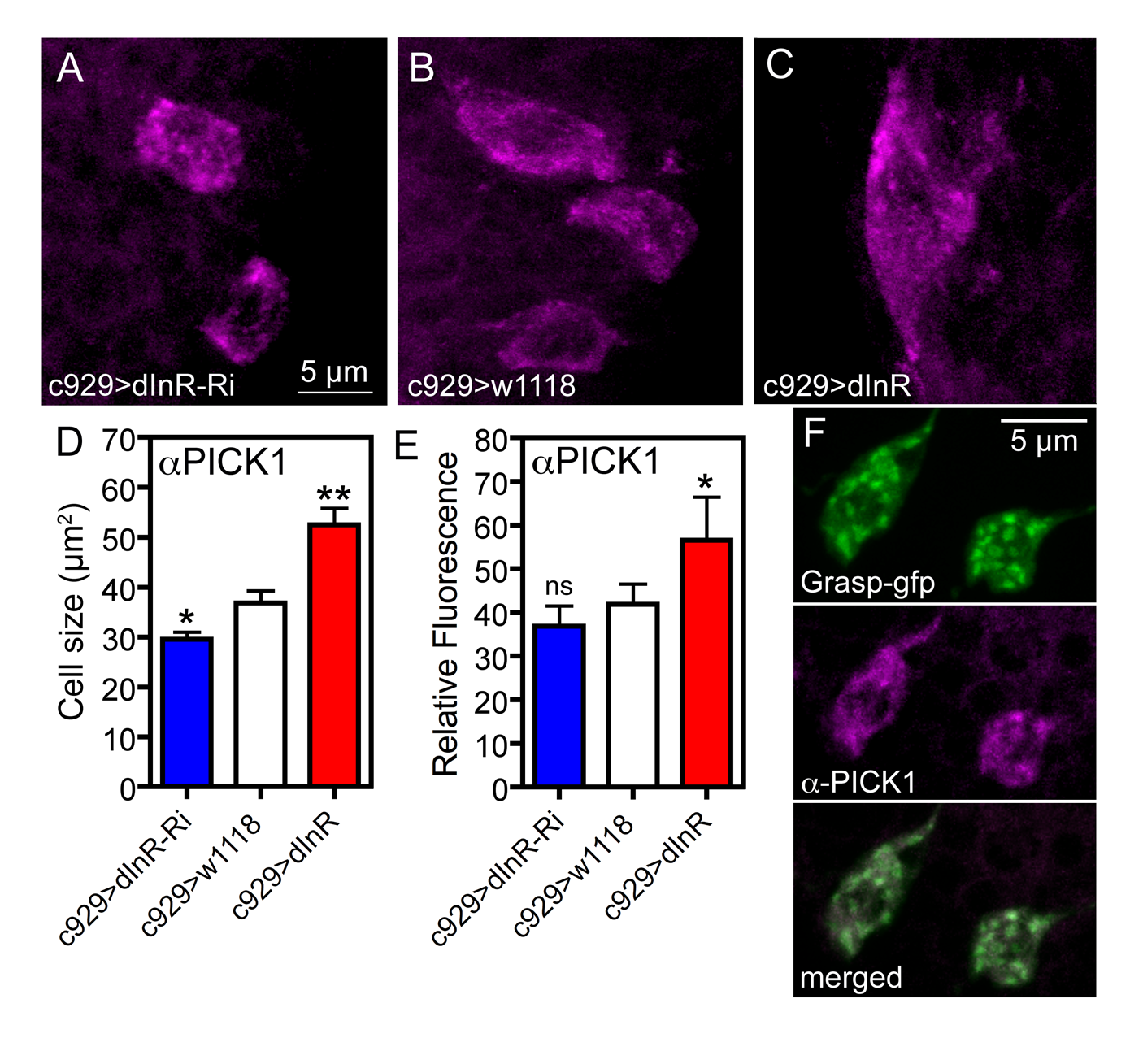

Supplement: Figure S13 — Over expression of the dInR in DIMM neurons increases PICK1 expression. A–C We used an antiserum to PICK1 to monitor the approximate abundance of trans-Golgi network units. The association of PICK1 immunolabeling with Golgi was determined in Drosophila brain neurons with a Golgi marker GRASP (see [74] and Fig. 10 F ). Over expression of dInR affected the PICK1 immunolabeled cell size as well as total relative PICK1 fluorescence in c929-Gal4 expressing neurons of the larval abdominal ganglia, quantified in D and E (*p<0.05, **p<0.01, n = 5 animals for each genotype from 3 crosses; unpaired Student's T-test). The increased PICK1 immunofluorescence indicates addition of trans-Golgi units or increased Golgi volume. No effect on PICK1 immunolabeling was seen after dInR-RNAi. F PICK1 immunolabeling in cell body of ABLK neuron is associated with UAS-Grasp-GFP expression. (TIF) [file pgen.1004052.s013.tif]

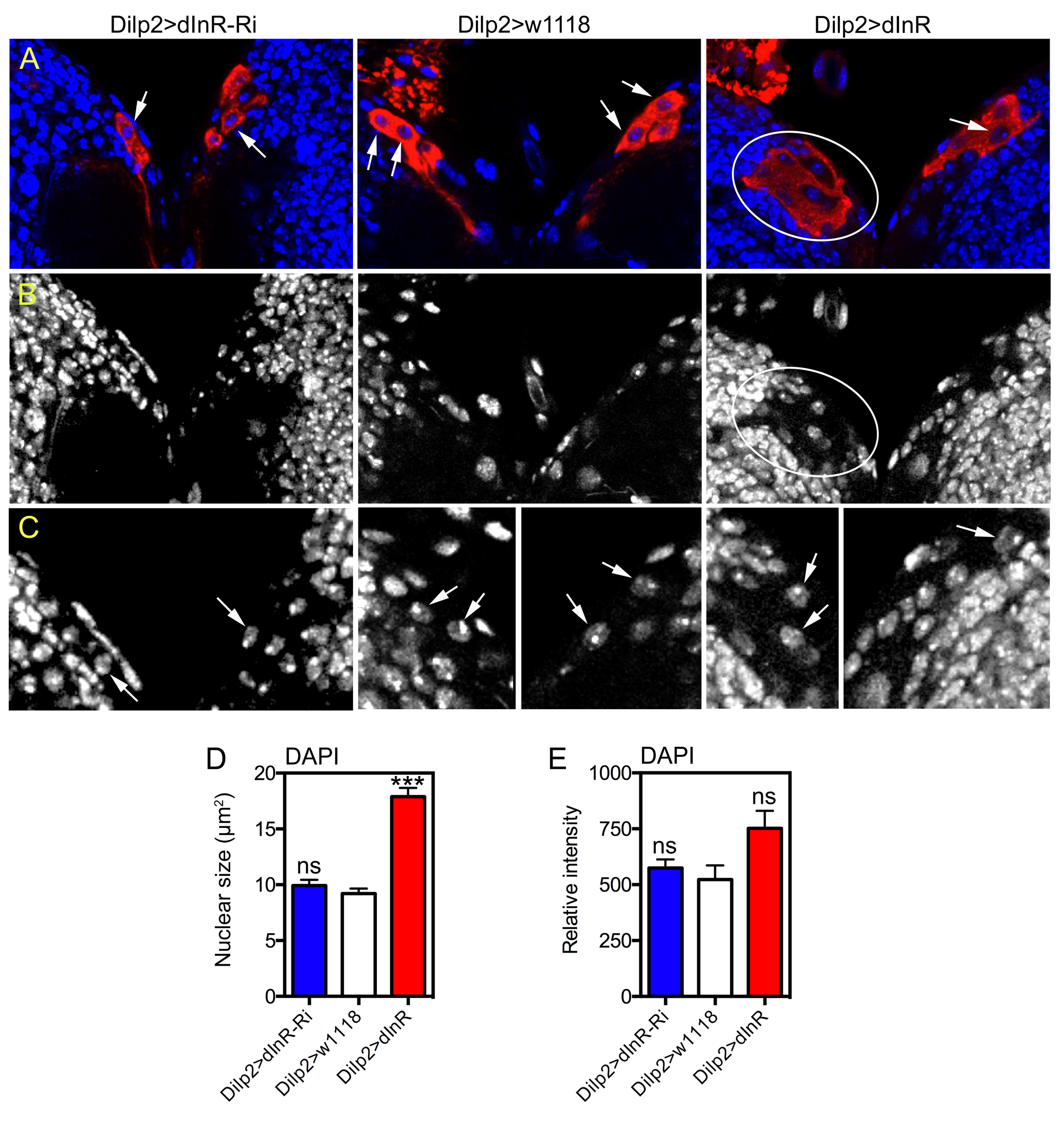

Supplement: Figure S14 — Over expression of dInR in IPCs leads to increased nuclear size. A After manipulations of dInR in IPCs (Using Dilp2-Gal4) the nuclear size in IPCs (labeled with anti-DILP2; red) was determined by DAPI staining (blue). B and C The nuclei of the IPCs indicated by arrows in A are shown after DAPI staining (IPC nuclei indicated by arrows in C). D Quantification of nuclear size in IPCs after dInR manipulations. Overexpression of the dInR induced larger nuclei (***p<0.001, Unpaired Students' T-test; n = 6–8 animals for each genotype from 3 crosses). E Quantification of total DAPI fluorescence (mean fluorescence multiplied by cell size) in IPCs after receptor manipulations. No significant change in fluorescence intensity was seen (for the Dilp2>dInR p>0.05, Unpaired Student's T-test; n = 6–8 animals for each genotype from 3 crosses). Note that the images of DAPI staining shown here are saturated to clearly visualize the nuclei; for intensity measurements, they were not saturated. (TIF) [file pgen.1004052.s014.tif]

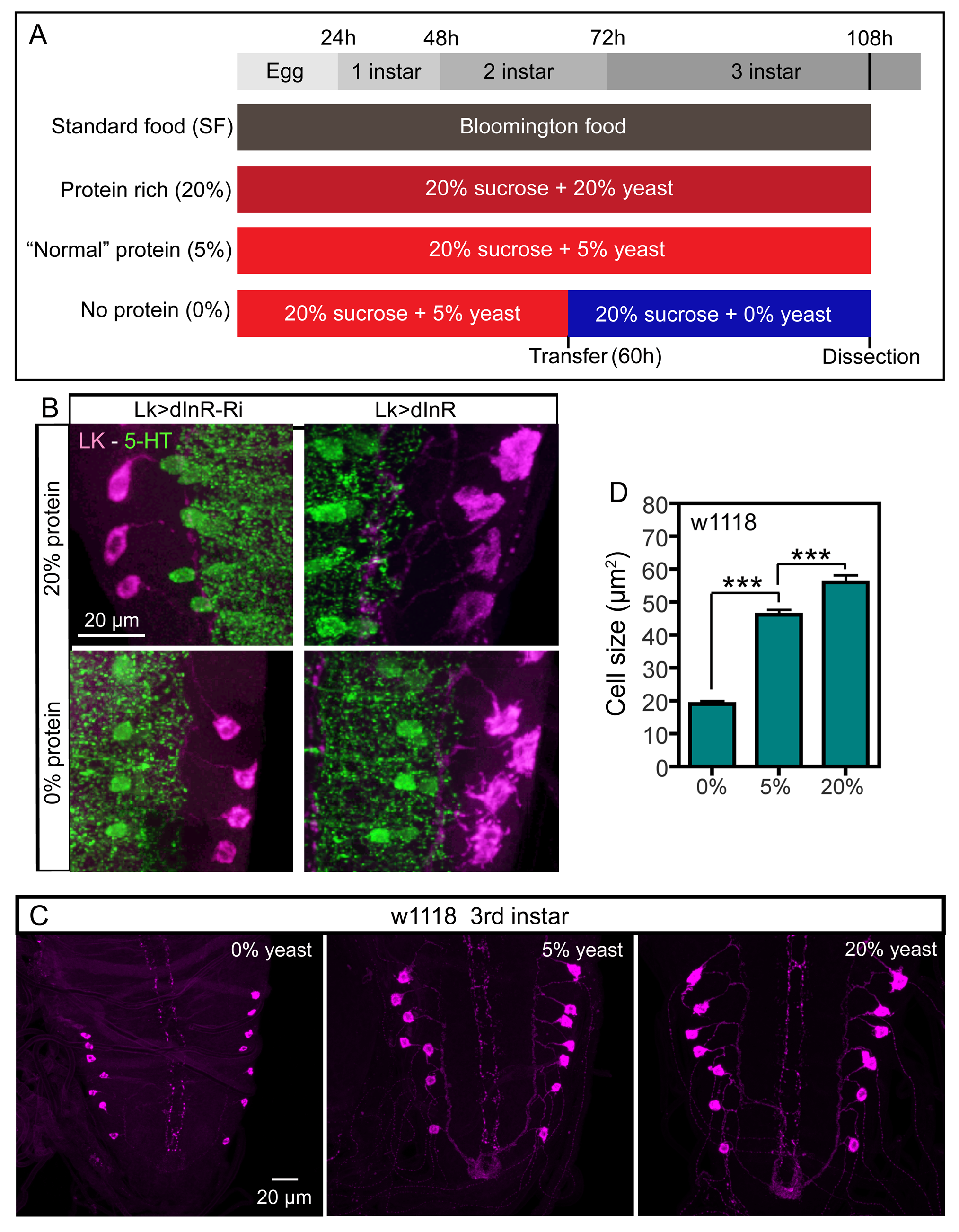

Supplement: Figure S15 — Protein levels in diet affect size of ABLKs. A The diets and feeding regimes used in experiments. Standard food is based on the one used by Bloomington Drosophila Stock Center. This is used in most experiments in this paper. Three experimental diets were tested for experiments in Fig. 11 and here in panels B–D. We combined 20% sucrose with 0, 5 and 20% yeast paste; two of these were based on sucrose and yeast only, and one a combination of 20% sucrose and 5% yeast until the proposed critical size of larvae was reached (at 60 h) and then 20% sucrose only. Wandering third instar larvae were used for imaging (about 108 h development). B Details of ABLK cell bodies (anti-LK; magenta) and 5-HT immunoreactive ones (green) in third instar larvae where dInR was manipulated in Lk-Gal4 neurons (and different protein diets). The whole ganglia are shown in Fig. 11A and B. C Cell bodies of ABLKs of wild type (w1118) larvae feed three different diets. D Quantification of ABLK cell body sizes in the diet experiment shown in C. Cell size increases significantly with protein levels (*p<0.05, **p<0.01, ***p<0.001, n = 6–13 larvae for each genotype from 3 crosses; unpaired Student's T-test). (TIF) [file pgen.1004052.s015.tif]

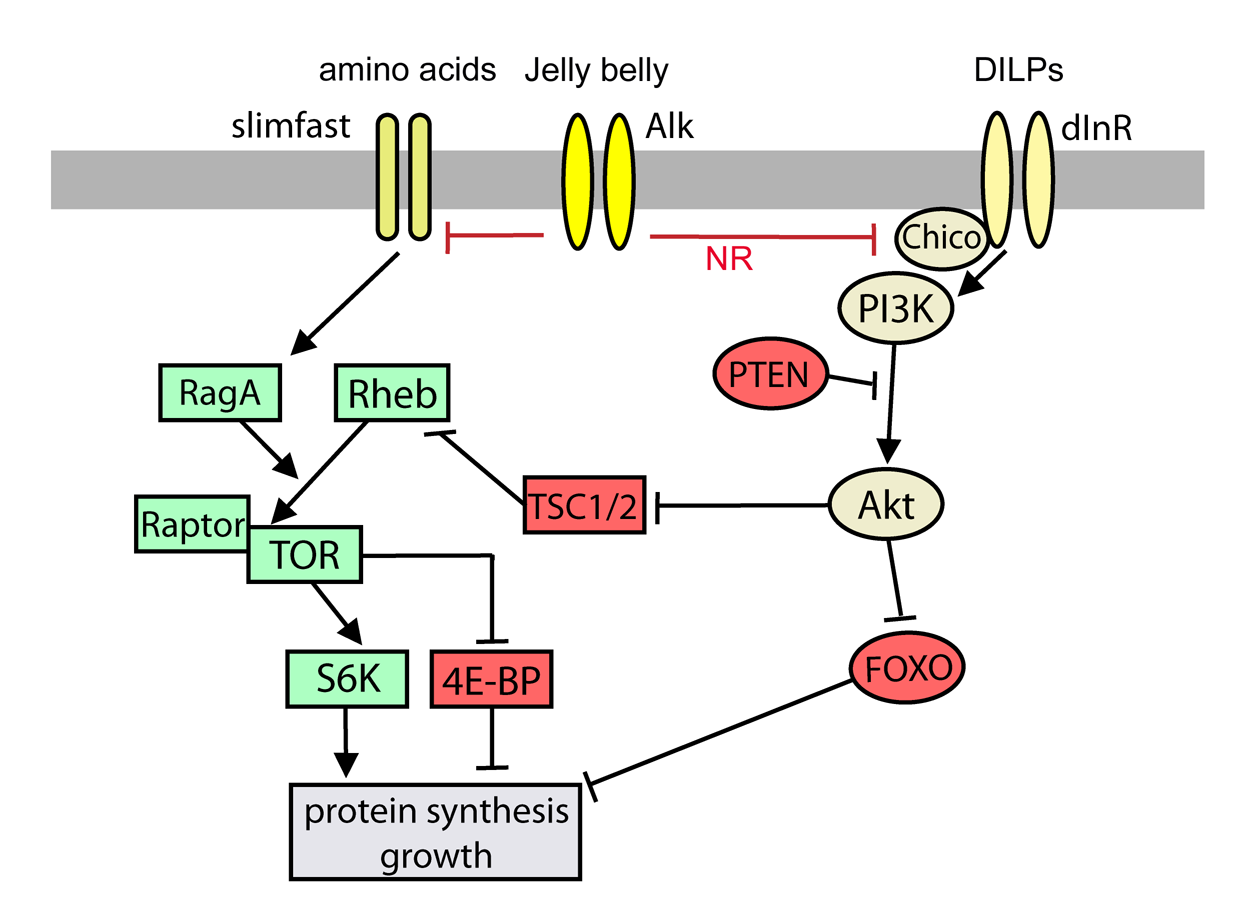

Supplement: Figure S16 — A scheme of the nutrient sensing TOR and insulin signaling pathways. This scheme is a compilation of several published ones (see [17], [47]) and display key features of interest in the present paper. The two pathways intersect at the level of Akt and TSC1/2 and converge on regulation of protein synthesis and growth (via ribosome biogenesis and translation apparatus). The Alk signaling pathway is activated only at nutritional restriction (NR) and inhibits the two other pathways to ensure CNS growth as a super sparing of this tissue at the cost of other tissues. However at low nutrients conditions the Alk signaling activates the pathway downstream the dInR (PI3K). Arrows depict activating signals and the T-shaped connectors inhibitory ones. (TIF) [file pgen.1004052.s016.tif]
