# Supplementary material for: Insulin/IGF-Regulated Size Scaling of Neuroendocrine Cells Expressing the bHLH Transcription Factor Dimmed in Drosophila
Source: PLoS Genet. 2013 Dec 26;9(12):e1004052. doi: 10.1371/journal.pgen.1004052 (PMC3873260; doi:10.1371/journal.pgen.1004052)
Supplement: Table S1 — Manipulations of dInR expression in different cell types: numerical data. This table provides numerical data for manipulations of genes in Dimm positive and Dimm negative peptidergic neuroendocrine cells. In this table only the PTTH neurons are DIMM negative. Values are given as means ± SEM. n = number of animals tested; *p<0.05, **p<0.01, ***p<0.001, ns not significant (Unpaired Student's T-test), data are presented as mean values ± SEM. L1 = 1st instar larva, L3 = 3rd instar larva, A 3 d = 3 d old adult flies, A 35 d = 35 d old adult flies, ant = anterior LK neurons, post = posterior LK neurons, R-neur = R-neurons of ellipsoid body. (DOCX) [file pgen.1004052.s017.docx]

| Gal4 | Cell type | Stage | Genetic manipulations and cell size (μm^2^) | | | |
| --- | --- | --- | --- | --- | --- | --- |
|  |  |  | *wildtype* | *dInR-Ri* | *dInR* | *dInR-CA* |
| *Lk* | **ABLKs** | L1 | 26.1±1.7  n=10 | 20.6±1.6  n=5, **ns**  p=0.0884 | 69.4±5.5  n=5, *******  p<0.0001 | - |
|  |  | L3 | 70.3±2.0  n=13 | 57.8±2.5  n=7, *******  p=0.0002 | 116.3±5.0  n=7, *******  p<0.0001 | 146.1±7.4  n=6, *******  p<0.0001 |
|  |  | A 3d  ant | 48.9±2.1 n=7 | 47.4±2.8 n=8, **ns**  p=0.6976 | 87.2± 6.5 n=9, *******  p<0.0001 | - |
|  |  | A 3d  post | 24.3±1.1 n=7 | 24.9±0.8 n=7, **ns**  p=0.6660 | 56.0±1.5 n=9, *******  p<0.0001 | - |
|  |  | A 35d ant | 52.2±2.6 n=10 | 38.6±2.3 n=6, ******  p=0.0039 | 105.5±5.8  n=7, *******  p<0.0001 | - |
|  |  | A 35d post | 39.6±2.2 n=10 | 24.4±1.0 n=6, *******  p=0.0002 | 84.8±12.6  n=7, *******  p=0.0008 | - |
| *Dilp2* | **IPCs** | L3 | 59.2±2.0 n=9 | 42.0±3.8  n=8, *****  p=0.011 | 83.0±5.2  n=6, *******  p=0.0003 | 85.1±5.8 n=6, *******  p=0.0003 |
|  |  | A | 84.0±5.4  n=10 | 40.7±4.3  n=9, *******  p<0.0001 | 101.5±5.9 n=7, *****  p=0.0497 | 132.7±22.4  n=7, *****  p=0.0256 |
| *ptth* | **PTTH** | L3 | 105.8±8.2 n=21 | 131.6±12.8n=15, **ns**  p=0.1942 | 99.02±6.9 n=10, **ns**  p=0.7191 | 101.2±5.1 n=6, **ns**  p=0.7708 |
|  |  | A  R-neur | 46.7±2.5 n=10 | 49.3±2.9 n=5, **ns**  p=0.5405 | 52.2±2.8 n=6, **ns**  p=0.7307 | - |
| *Dilp7* | **DILP7**  **(A6-9+DP)** | L3 | 66.1±1.4  n=18 | 57.7±2.4 n=10, ******  p=0.0036 | 120.3±4.6 n=12, *******  p<0.0001 | - |
| *c929* | **Tv1-3** | L3 | 62.1±5.1  n=8 | 48.2±2.9 n=7, *****  p=0.0416 | 97.3±4.1  n=6, *******  p=0.0003 | - |
| *pdf* | **Abd PDF** | L3 | 47.9±1.9  n=8 | 45.0±2.3  n=8, **ns**  p=0.3679 | 77.0±7.3  n=9, ******  p=0.0024 | - |
|  | **l-LNv PDF** | A | 86.9±12.2 n=6 | 97.7±10.2 n=6, **ns**  p=0.5151 | 185.6±10.7 n=9, *******  p<0.0001 | - |
| *ok6* | **Brain intern** | L3 | 61.9±5.1  n=6 | 57.1±1.9 n=6, **ns**  p=0.4359 | 64.3±6.1 n=6, **ns**  p=0.7783 | 70.6±4.5 n=6, **ns**  p=0.2746 |
|  | **Abd intern** | L3 | 39.8±3.5  n=6 | 43.1±4.3 n=6, **ns**  p=0.5860 | 39.1±2.5 n=6, **ns**  p=0.8760 | 38.8±1.8 n=6, **ns**  p=0.8141 |
|  | **Motoneuron** | L3 | 83.5±4.0  n=6 | 82.7±5.1 n=6, **ns**  p=0.8950 | 89.7±4.5 n=6, **ns**  p=0.3047 | 74.0±3.1 n=6, **ns**  p=0.1703 |
| *Trh* | **5-HT (A1-9)** | L3 | 46.1±2.2  n=11 | 45.9±1.6  n=12,  **ns**  p=0.9644 | 45.4±1.6  n=11, **ns**  p=0.8145 | - |

**Table S1** Manipulations of dInR expression in different cell types: numerical data
