# Supplementary material for: Insulin/IGF-Regulated Size Scaling of Neuroendocrine Cells Expressing the bHLH Transcription Factor Dimmed in Drosophila
Source: PLoS Genet. 2013 Dec 26;9(12):e1004052. doi: 10.1371/journal.pgen.1004052 (PMC3873260; doi:10.1371/journal.pgen.1004052)
Supplement: Table S2 — Manipulations of PI3K and Rheb expression in different cell types: numerical data. This table provides numerical data for manipulations of genes in Dimm positive peptidergic neuroendocrine cells. Values are given as means ± SEM. n = number of animals tested; *p<0.05, **p<0.01, ***p<0.001, ns not significant (Unpaired Student's T-test), data are presented as mean values ± SEM. L3 = 3rd instar larva, A 3 d = 3 d old adult flies, A 35 d = 35 d old adult flies, ant = anterior LK neurons, post = posterior LK neurons, R-neur = R-neurons of ellipsoid body. (DOCX) [file pgen.1004052.s018.docx]

**Table S 2** Manipulations of PI3K and Rheb expression in different cell types: numerical data

| Gal4 | Cell type | Stage | Genetic manipulations and cell size (μm^2^) | | | | |
| --- | --- | --- | --- | --- | --- | --- | --- |
|  |  |  | *wildtype* | *PI3K-DN* | *PI3K* | *Rheb-Ri* | *Rheb* |
| *Lk* | **ABLKs** | L3 | 70.3±2.0  n=13 | 46.2±1.5  n=8, *******  p<0.0001 | 152.4±6.5  n=7, *******  p<0.0001 | 65.4±2.7  n=8, **ns**  p=0.1554 | 135.7±7.1  n=7, *******  p<0.0001 |
|  |  | A 3d  ant | 60.1±2.3 n=9 | 49.4±2.2  n=6, ******  p=0.0077 | 132.8±9.1 n=9, *******  p<0.0001 | 48.3±1.3 n=5, ******  p=0.0038 | 133.3±12.1  n=9, *******  p<0.0001 |
|  |  | A 3d  post | 30.7±1.7 n=9 | 24.6±1.1 n=6, *****  p=0.0224 | 62.9±3.1 n=9, *******  p<0.0001 | 22.5±0.8 n=5, ******  p=0.0060 | 71.3±5.3  n=9, *******  p<0.0001 |
| *Dilp2* | **IPCs** | L3 | 59.2±2.0 n=9 | 37.7±1.6 n=8, *******  p<0.0001 | 80.6±11.3  n=7, ******  p=0.0094 | - | - |
|  |  | A | 84.0±5.4  n=10 | 52.1±1.8 n=7, *****  p=0.0295 | 86.1±4.7 n=5, ******  p=0.0080 | - | - |
| *ptth* | **PTTH** | L3 | 105.8±8.2 n=21 | 102.2±6.1n=5, **ns**  p=0.8349 | 147.1±5.8  n=10, ******  p=0.0029 | 85.9±11.8 n=8, **ns**  p=0.3565 | 197.9±22.4  n=8, *******  p<0.0001 |
|  |  | A  R-neur | 46.7±2.5 n=10 | - | - | 45.1±3.3 n=5, **ns**  p=0.1872 | 62.4±1.3 n=6, *******  p<0.0001 |
